# Ligand-Mediated Defects Unlock Fast and Regenerable CO_2_ Capture in NICS-24 Metal–Organic Framework

**DOI:** 10.1021/jacs.6c04820

**Published:** 2026-06-16

**Authors:** Klara Klemenčič, Petar Djinović, Miha Okorn, Jakob Höfferle, Andraž Krajnc, Durga Acharya, Cara M. Doherty, Dana Marinič, Blaž Likozar, Nataša Zabukovec Logar, Matjaž Mazaj

**Affiliations:** † 68913 National Institute of Chemistry, Hajdrihova ulica 19, 1000 Ljubljana, Slovenia; ‡ University of Nova Gorica, Vipavska cesta 13, 5000 Nova Gorica, Slovenia; § Faculty of Chemistry and Chemical Technology, University of Ljubljana, Večna pot 113, 1000 Ljubljana, Slovenia; ∥ CSIRO Manufacturing, Clayton, VIC 3168, Australia; ⊥ Faculty of Chemistry and Chemical Engineering, University of Maribor, Smetanova 17, 2000 Maribor, Slovenia

## Abstract

Indoor CO_2_ capture is an emerging strategy to improve air quality while reducing the energy cost of ventilation. Practical adsorbents must exhibit high affinity at low partial pressures, fast sorption kinetics, and energy-efficient regeneration. Here, we show that the Zn–oxalate guanazolate framework NICS-24, a promising platform for CO_2_ capture at 400–1000 ppm, can be transformed into a high-performance indoor sorbent via ligand-mediated defect engineering (LMDE). Postsynthetic treatment with CF_3_- and SCH_3_-functionalized azolate linkers (denoted as FMeTz and SMeTz) induces an equilibrium-limited partial Zn-leaching process that generates vacancy-type defects while preserving the long-range crystallinity and Zn–linker connectivity. Comprehensive characterization (PXRD, EDS, ICP, XPS, PALS, FTIR, and multinuclear SSNMR) reveals subtle unit-cell contraction, increased local disorder, and modest expansion of accessible ultramicropore free volume. These defect-related changes markedly enhance CO_2_ adsorption and transport. At 25 °C and 1000 ppm, the NICS-24-modified materials show more than a 2-fold increase in uptake (0.36 to 0.77 mmol/g), along with rapid kinetics and improved thermal-swing regeneration. The SMeTz-treated material retains high working capacity after regeneration at 70 °C and exhibits stable cycling. In situ DRIFTS and ex situ SSNMR confirm reversible physisorption dominated by interaction with defect-associated O–H/N–H environments, directly linking vacancy-type defect domains to enhanced low-pressure uptake, accelerated transport kinetics, and regenerability. Although water adsorption remains competitive, the frameworks are hydrolytically robust, highlighting LMDE-modified NICS-24 as a promising platform for indoor CO_2_ capture under dry conditions.

## Graphical abstract

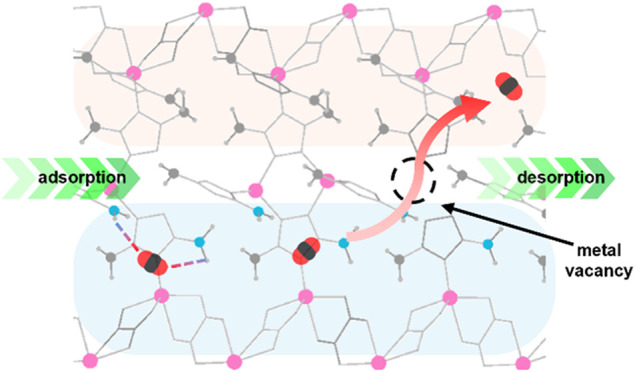

## Introduction

Metal–organic frameworks (MOFs) represent one of the most structurally versatile classes of porous materials, owing to the modular combination of metal nodes and organic linkers, which enables atomic-level control over pore architecture, surface chemistry, and framework topology.^1–6^ This structural tunability has made MOFs highly attractive for applications in catalysis, separations, sensing, and gas storage.^7,8^ In particular, their ability to tailor the pore environments provides a level of control over adsorption and diffusion that is difficult to achieve with other porous materials such as zeolites, activated carbons, or polymer networks.

This tunability is especially relevant in the context of rising anthropogenic CO_2_ emissions. In addition to long-term climate effects, elevated CO_2_ levels, which often exceed 1000 ppm, have been associated with immediate health concerns, including respiratory effects and cognitive impairment.^9^ Capturing CO_2_ from low-concentration sources, such as indoor environments (∼1000–2000 ppm) or ambient air (∼400 ppm), remains a significant challenge. At such low partial pressures, adsorbents must combine strong yet reversible binding with fast mass transport and tolerance to competing species such as water. MOFs have shown considerable promise under these conditions, particularly due to their tunable pore chemistry.^10–12^

A key strategy for improving the level of CO_2_ capture in MOFs is pore engineering. Ultramicropores can enhance CO_2_ affinity through confinement effects and electrostatic complementarity,^13^ while larger pores facilitate faster diffusion but, on the other hand, require additional functionalization to maintain high uptake at low pressures.^14^ High-performing systems therefore rely on a delicate balance between pore size, chemical functionality, and accessibility.^15,16^ However, increasing adsorption strength often comes at the expense of slower transport, which highlights a fundamental coupling between binding affinity and diffusivity in confined pore systems.^17^ Overcoming this trade-off remains a crucial challenge in the design of efficient sorbents for low-concentration CO_2_ capture.^18^

Defect engineering has emerged as a powerful approach to address this limitation.^19–23^ Structural defects, such as missing linkers or metal nodes, can modify pore connectivity, create new adsorption environments, and introduce chemical heterogeneity that is not accessible through ideal crystalline structures. These defects are commonly introduced ether during synthesis or through postsynthetic treatments, including postsynthetic ligand exchange (PSE).^24–26^ While PSE is typically employed to incorporate new functional groups into the framework, the exchange is often incomplete, generating heterogeneous structures.^25,27,28^ Such processes can lead to the formation of defect-rich environments, including under-coordinated metal sites and vacancy-type defects, which influence both adsorption thermodynamics and transport behavior.

Several studies have demonstrated that such defect structures can enhance the CO_2_ capture performance. For example, missing-linker, missing-cluster defects in UiO-66 have been shown to create polar adsorption sites and improve uptake at low pressures,^29–34^ while defect-mediated modifications in MOF-74,^35^ NU-125,^25^ and MIL-101­(Cr) have been linked to improved performance under diluted CO_2_ conditions.^24,36–38^ In many cases, however, these approaches focus primarily on modifying adsorption energetics through chemical functionalization, whereas the role of defects in reshaping transport pathways remains less explored.

In this work, we extend postsynthetic strategies to the Zn–oxalate guanazolate framework NICS-24, previously identified as a promising platform for CO_2_ capture at low concentrations. While its strong adsorption affinity is advantageous at low pressures, the intrinsic competitive water adsorption under humid conditions represents a fundamental limitation. Rather than targeting conventional pore functionalization, we explore postsynthetic treatments to perturb the local environment and influence transport characteristics within the framework. Therefore, NICS-24 was treated with thiomethylaminotriazole (SMeTz) and trifluoromethyltriazole (FMeTz), selected as coordinating modulators capable of interacting with metal nodes. By combining detailed structural characterization with equilibrium and dynamic CO_2_ sorption analysis, we examined how ligand-assisted treatments affect adsorption behavior beyond simple pore chemistry modification. This work examines whether azolate-assisted postsynthetic treatment can transform NICS-24 from a strongly binding and diffusion-limited ultramicroporous framework into a defect-engineered sorbent in which vacancy-type defects address the trade-off between low-pressure CO_2_ affinity and mass transport.

## Results and Discussion

### Structural Insights

To probe the effect of linker environment on CO_2_ capture performance, two triazolate-based modulators with distinct substituents were selected: FMeTz (−CF_3_) and SMeTz (−SCH_3_). While both retain the same coordinating triazolate core, their functional groups differ significantly in electronic character and polarity. The CF_3_ group is strongly electron-withdrawing and relatively rigid, whereas the −SCH_3_ group is more polarizable and a weaker electron donor. These modulators are intended to act as chemical perturbants that influence Zn–ligand bond stability during postsynthetic treatment.

A series of modified NICS-24 samples were prepared using varying loadings of linkers FMeTz and SMeTz (Figure 1a, Table S1) to identify the modification level that maximizes CO_2_ capture performance while preserving the structural integrity of the parent framework. PXRD patterns show that samples containing up to 40% FMeTz and 150% SMeTz (relative to the guanazole in pristine NICS-24) show high phase purity and no detectable shifts in Bragg peak positions compared to pristine NICS-24 (Figures 1b, S1, and S2), confirming that the ligand-assisted treatment process does not alter the long-range crystal structure or topology within this modification region. Only at higher loadings of FMeTz or SMeTz, additional weak-intensity reflections attributable to secondary phases emerge (Figures S3 and S4), which are consistent with Zn­(II)–azolate complexes as suggested from EDS analysis (Figure S5). Within the phase-pure regime, a systematic broadening of the most intense reflection corresponding to the (−1 2 0) plane of the NICS-24 structure is observed across the series, indicating that although the framework remains topologically intact, its local structural coherence is affected by the modification (Figures 1c and S6). Preliminary CO_2_ sorption screening up to 1000 ppm at 0 °C (Figures S7 and S8) was used to select the most promising materials for detailed analysis. For FMeTz-modified samples, a loading of 40% relative to the amount used for pristine NICS-24, yielded optimal uptake of 1.52 mmol/g, whereas SMeTz showed maximal performance at a loading of 60% (1.72 mmol/g). The corresponding samples, 40FMeTz and 60SMeTz, were therefore selected for further structural and capture performance evaluation. To assess the accuracy of their preparation, the repeatability of their CO_2_ capture performance was also tested (Figure S9). To elucidate subtle structural modifications not apparent from qualitative XRD observations, Le Bail refinements were performed (Figure 1d and Table S2). The unit-cell volumes of both FMeTz- and SMeTz-modified samples are consistently smaller than that of pristine NICS-24, even at the lowest modification level (10%). This contraction indicates that structural adjustments occur at the earliest stages of ligand treatment, which most likely arises either from partial incorporation of functionalized linkers, defect formation, or partial displacement of the native linker rather than full substitution. Increasing the amount of modulators in the ligand-treatment process does not induce further contraction, and the unit-cell volume becomes essentially invariant across the series. This suggests that the ligand exchange rate or density of defect generated sites within the NICS-24 is limited. After this “saturation of modification,” the framework reaches a structurally stable state that no longer responds to additional secondary ligand exposure. Previously mentioned peak broadening may originate from either reduced crystallite size or increased local structural disorder. However, SEM analysis shows that particle sizes and morphologies remain unchanged after modification (Figure S10), effectively excluding crystallite-size reduction as the cause. The broadening must therefore arise from local structural distortions or increased microstrain introduced during partial ligand exchange or defect formation. The similar contraction and broadening trends observed for both FMeTz- and SMeTz-modified series further support that these effects stem from the ligand-treatment process, rather than from differences in linker size, polarity, or electronic character.

**Figure 1:**
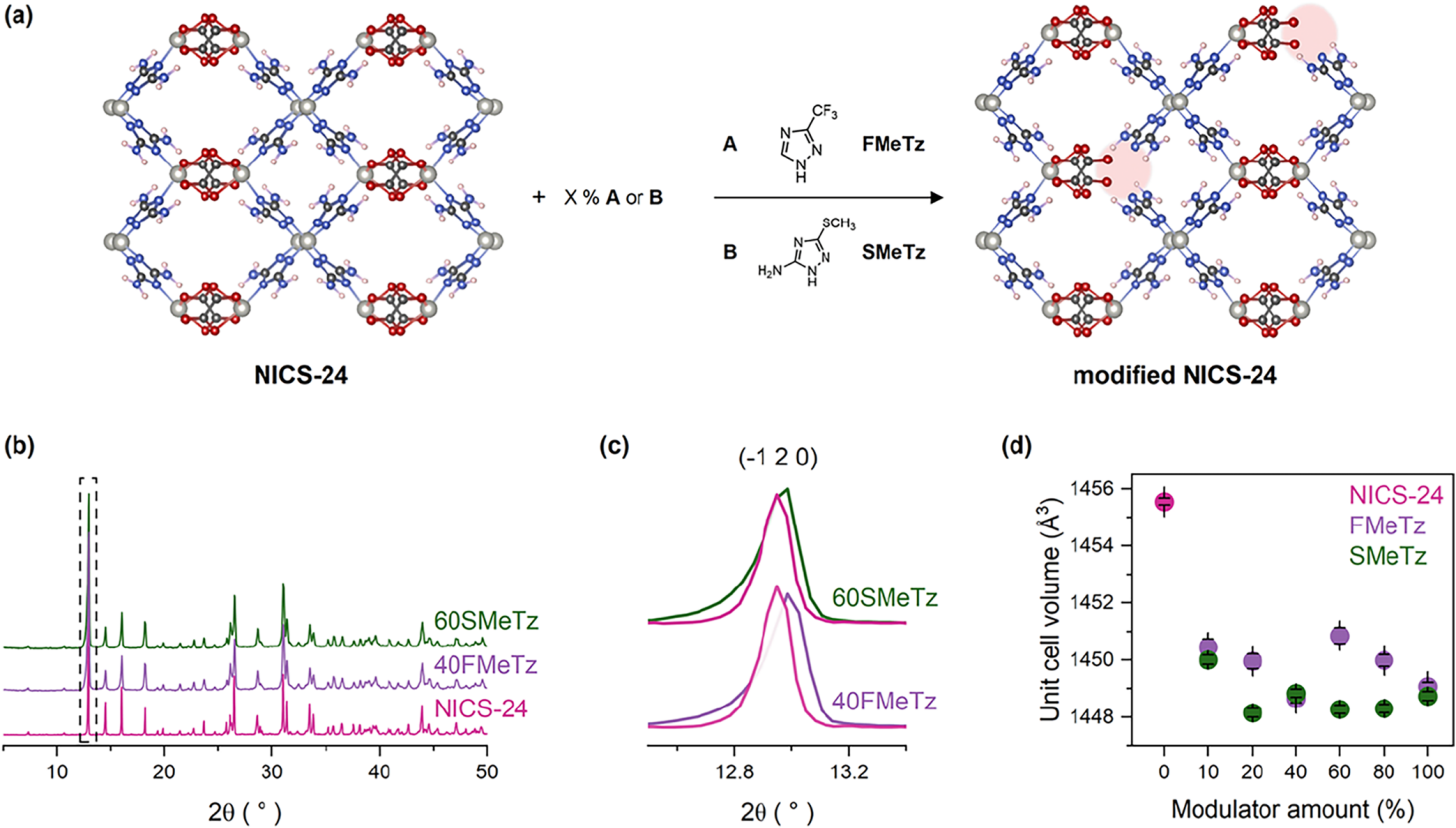
(a) Structural representation of NICS-24 showing Zn–oxalate–guanazolate connectivity with larger and smaller types of open pore channels. Schematic illustration of the ligand-treatment process with different loadings of functionalized linkers (FMeTz and SMeTz) (X = 10–100%). (b) Powder X-ray diffraction (PXRD) patterns of pristine NICS-24 and selected modified samples (40FMeTz and 60SMeTz), demonstrating the retention of the crystal structure upon ligand treatment. (c) Magnified view of the (−1 2 0) reflection region highlighting systematic peak broadening after modification. (d) Unit-cell volumes extracted from Le Bail refinements showing an initial contraction upon modulation that saturates at low modification levels, consistent with partial exchange and/or defect formation.

The next step was to determine whether the treatment resulted in incorporation of the triazolate modulators in the NICS-24 framework and, if present, to quantify their extent. TG analysis (Figure S11) shows that pristine NICS-24 yields a ZnO residue of 39.2 wt %, which is in excellent agreement with the theoretical value (39.2 wt %). In contrast, the modified samples exhibit slightly reduced residues (∼4 wt % lower for 40FMeTz and ∼1.5 wt % lower for 60SMeTz), indicating a higher relative organic content. The lower ZnO residue observed for the modified samples does not arise from Zn loss during thermal treatment but can be either due to the Zn leaching during the ligand-treatment process or substantial incorporation of the triazolate modulators having higher molar mass than the pristine guanazole in the case of modified materials. To confirm one of these scenarios, an EDS analysis was performed. The Zn/N molar ratios of 40FMeTz and 60SMeTz, determined from 20 randomly selected crystallites for each sample, fall below the ideal NICS-24 stoichiometry value of 0.2 (Figures 2a and S12 and Table S3) and align with the trend expected for Zn leaching rather than bulk modulator incorporation. Fluorine and sulfur are detectable at levels close to the detection limit (below 1 mol %) with no visible clustering on the crystallites (Figure S13). This is most likely related to the presence of trace surface-associated species or residual impurities rather than bulk ligand replacement. Thus, rather than producing a conventional mixed-linker framework through substantial incorporation of FMeTz or SMeTz, the postsynthetic treatment operates predominantly as ligand-mediated defect engineering (LMDE), in which the functionalized azolate ligands promote partial Zn leaching and vacancy-type defect formation, while direct linker substitution remains limited. Liquid NMR (Figure S14) and XPS (Figure S15) confirm the absence of modulators within the investigated modified samples. Zn leaching is further supported by ICP-OES and XPS analyses. ICP-OES reveals a decrease in Zn content from 32.0 wt % in pristine NICS-24 to 30.9 wt % in 40FMeTz and 30.6 wt % in 60SMeTz, corresponding to an apparent Zn-defect density of approximately 3.4% and 4.4%, respectively. XPS shows a consistent reduction of Zn 2p3 signal and increase of N 1s, C 1s, and O 1s signals at the surface (Figure S15). The agreement between bulk-sensitive ICP-OES and surface-sensitive XPS, together with the spatial heterogeneity in EDS mapping, indicates that the Zn deficiency is not confined to the external surface but is distributed throughout the crystallites.

**Figure 2:**
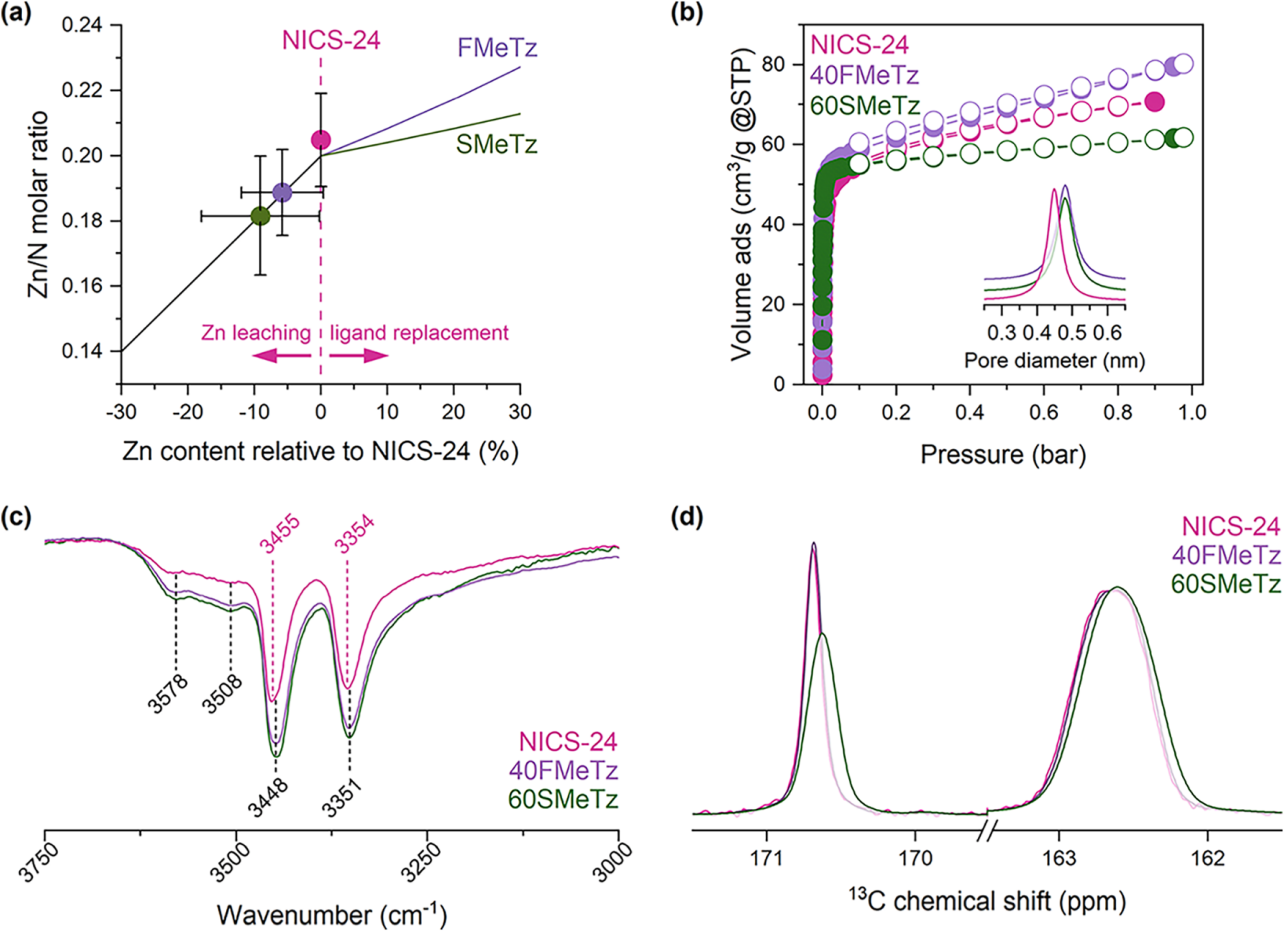
Structural and spectroscopic consequences of LMDE modification in NICS-24. (a) Zn/N molar ratios obtained from EDS analysis (averaged over 20 crystallites per sample) plotted as a function of either Zn leaching or ligand replacement, together with theoretical trends (full lines). The experimentally determined values for 40FMeTz and 60SMeTz samples fall on the Zn-deficiency side of the reference NICS-24 stoichiometry (Zn/*N* = 0.2), indicating that modification predominantly induces partial Zn leaching rather than extensive linker substitution. (b) CO_2_ adsorption isotherms measured at 273 K for pristine NICS-24, 40FMeTz, and 60SMeTz, demonstrating retained microporosity after modification. Inset: apparent pores size distributions derived from PALS, showing a slight shift toward larger ultramicropore sizes for the modified samples, consistent with defect-mediated local free-volume expansion. (c) FTIR spectra in the N–H/O–H stretching region highlighting changes induced by LMDE. While the main bands at ∼ 3455 and 3354 cm^–1^ are shifted only marginally to 3448 and 3351 cm^–1^ respectively, modified samples exhibit the emergence of weak bands at 3582 and 3507 cm^–1^, consistent with the formation of weakly hydrogen-bonded or defect-associated N–H environments following partial Zn–node disruption. (d) Detailed comparison of ^1^H–^13^C CP-MAS NMR spectra of individual as-prepared samples, highlighting the retention of the guanazolate environment, but a subtle upfield shift and broadening of the oxalate peak in 60SMeTz relative to NICS-24 and 40FMeTz samples.

Changes in metal-node integrity and defect density are expected to directly influence textural properties of NICS-24, making porosity analysis essential for understanding the CO_2_ capture behavior. Nitrogen physisorption at 77 K exhibits negligible N_2_ uptake across the entire pressure range for pristine NICS-24 and both the FMeTz- and SMeTz-modified materials (Figure S16). Nitrogen inaccessibility persists even though the diffusion is expected to be enhanced by defect formation. Low N_2_ uptake is a consequence of kinetic diffusion limitations at cryogenic temperature, arising from ultramicropores.^39^ Considering these limitations, porosity was alternatively evaluated using CO_2_ adsorption isotherms measured at 273 K (Figures 2b and S17), which are more suitable for probing ultramicropores. Nevertheless, CO_2_ sorption is used here primarily to assess relative uptake behavior and changes in accessible microporosity, rather than to define absolute textural parameters. This limitation arises from strong specific CO_2_–surface interactions.^40,41^ All samples exhibit Type I isotherms, confirming retention of a microporous framework. Relative to pristine NICS-24, the LMDE-treated materials display higher CO_2_ uptake across the full pressure range, consistent with increased pore accessibility and/or modified adsorbate–framework interactions. Apparent surface areas were estimated from the CO_2_ adsorption isotherms using a BET-type analysis (Figure S18, Table S4). The 40FMeTz-modified sample exhibits a gradual approach to saturation, reaching a CO_2_ uptake of 80 cm^3^/g (STP) at 1 bar, corresponding to a BET surface area of 322 m^2^/g. The SMeTz-modified sample shows a similarly shaped Type I isotherm, but with slightly lower overall uptake and surface area of 307 m^2^/g. Nevertheless, both samples show significant enhancement of *S*_BET_ values from pristine material (231 m^2^/g).

Quantitative pore-size distribution derived from CO_2_–NLDFT are inherently limited for polar, defect-containing MOFs, as available kernels are based on rigid carbon slit-pore models and cannot adequately describe the energetic heterogeneity, framework flexibility, and local disorder (Figure S19). As a result, the absolute pore sizes obtained from CO_2_–NLDFT should be regarded as qualitative indicators of ultramicroporosity rather than precise structural metrics.^42,43^ Positron annihilation lifetime spectroscopy (PALS) was therefore employed to obtain further information on the pore distribution (Figures 2b (inset) and S20, Table S5). PALS uses positrons to directly probe the free-volume elements independent of gas diffusion or adsorption energetics, making it suitable for ultramicroporous and diffusion-limited systems.^44,45^ The results indicate a unimodal pore distribution for all samples. Pristine NICS-24 exhibits an o-Ps lifetime (tau3) of 1.416 ± 0.005 ns, corresponding to an average pore diameter of 0.449 ± 0.001 nm. Upon modification, tau3 increases to 1.544 ± 0.011 ns for 40FMeTz and 1.533 ± 0.006 ns for 60SMeTz, yielding slightly larger average pore diameters of 0.479 ± 0.002 nm and 0.476 ± 0.001 nm, respectively. This slight but systematic increase indicates a modest expansion of the accessible ultramicropore free volume, consistent with local structural relaxation and/or defect formation induced during the ligand-mediated process. Notably, the corresponding o-Ps intensity (I3) decreases from 30.9 ± 0.2% (NICS-24) to 26.5 ± 0.3% (40FMeTz) and 26.0 ± 0.2% (60SMeTz), suggesting a reduced fraction of uniform free-volume sites, plausibly due to increased structural heterogeneity associated with defect generation.

FTIR spectroscopy provides complementary evidence for local defect formation in LMDE-modified NICS-24 that is consistent with partial disruption of the Zn–guanazolate coordination node (Figures 2c and S21). In the parent framework, Zn­(II) is coordinated by the N atoms of the guanazolate ring. Partial Zn leaching is therefore expected to generate uncoordinated N sites that can become protonated, increasing the population of the framework’s N–H groups. In agreement with this expectation, the two dominant N–H stretching bands of pristine NICS-24 at 3455 cm^–1^ and 3354 cm^–1^ remain essentially unchanged after LMDE, indicating the preservation of the overall coordination environment. At the same time, two weak bands at 3578 and 3508 cm^–1^ emerge or increase markedly in intensity for both 40FMeTz and 60SMeTz. These bands are consistent with free N–H/O–H environments, as expected upon partial leaching of Zn­(II) from the framework. In addition, the modified samples exhibit a broadened band extending from approximately 4000 to 3000 cm^–1^, which is commonly associated with strongly hydrogen-bonded O–H and/or N–H stretching vibrations^46^ suggesting the increased H-bonding heterogeneity and defect density within the modified structure.^46,47^ Notably, no distinct vibrational features attributable to −CF_3_ or −SCH_3_ groups are observed, indicating that incorporation of the functionalized ligands into the framework is negligible. This additionally supports the ligand-mediated defect formation rather than its structural incorporation during the postsynthetic treatment.

Multinuclear solid-state NMR (SSNMR) spectroscopy was employed to probe the local structural consequences of LMDE and to assess whether the structural integrity is preserved after postsynthetic modification. Comparison of the ^1^H MAS, ^1^H–^13^C CP-MAS, and ^1^H–^15^N CP-MAS spectra of pristine NICS-24 with those of the SMeTz- and FMeTz-modified samples (Figure S22) reveals that the chemical shifts of the framework-building guanazolate and oxalate units remain largely unchanged, indicating the preservation of the primary Zn–ligand connectivity and overall framework architecture, consistent with previous reports on NICS-24.^48^ No resonances characteristic of the exchanged triazolate linkers are observed (Figure S23). These observations confirm that the treatment does not lead to significant bulk incorporation of the functionalized triazolate linkers. Instead, associated defect formation via Zn leaching dominates over direct linker substitution. Despite the preservation of the guanazolate environment, subtle but systematic changes are observed in the oxalate ^13^C chemical shift region (δ ≈ 171–169 ppm) when comparing individual materials (Figure 2d). The slight upfield shift and broadening of the oxalate peak in 60SMeTz relative to both pristine NICS-24 and 40FMeTz indicates a partial loss of Zn coordination and the presence of uncoordinated or weakly coordinated oxalate species.^26,49^ Such species are more prone to forming strong hydrogen bonds with guest molecules or can even undergo partial protonation upon hydration, shifting the ^13^C resonance toward that of protonated oxalic acid.^50–52^ In addition to the oxalate perturbation, the guanazolate ^13^C resonance exhibits a small upfield shift, consistent with subtle changes in the local electronic environment (e.g., partial Zn–N decoordination and/or altered hydrogen bonding), although distinct protonated guanazolate species are not resolved in the present spectra.

The structural analyses collectively highlight a fundamental distinction between the LMDE process used to generate NICS-24 from CALF-20 and the present postsynthetic treatment of NICS-24 with functionalized triazoles (Figure S24). In the former case, extensive ligand replacement and framework reorganization yields NICS-24, which is likely to present a thermodynamically stable phase. Once formed, the robust Zn–guanazolate connectivity of NICS-24 strongly limits further large-scale restructuring or true linker substitution. As a result, LMDE favors partial metal-node disruption and defect formation rather than ligand exchange.

The observed Zn deficiency is best rationalized by a ligand-assisted metal-leaching process governed by competitive coordination equilibria. During treatment with excess functionalized azolate ligands, free FMeTz or SMeTz can compete with the framework linkers for Zn^2+^ coordination,^53^ leading to the partial extraction of Zn­(II) from NICS-24 nodes and the formation of vacancy-type defects. For both FMeTz and SMeTz series, this interpretation is supported by the emergence of additional PXRD reflections at higher ligand loadings (Figures S3–S5), which are consistent with the secondary Zn–azolate coordination phases. These species may act as thermodynamic sink for leached Zn­(II),^54^ shifting the equilibrium toward limited metal removal from the framework. The provided data support that only a minor fraction of framework Zn^2+^ is removed and stabilized by excess azolate ligands, while sufficient Zn–linker connectivity is retained to preserve the overall NICS-24 framework.

### CO_2_ Capture Performance

After establishing that LMDE primarily induces defect formation via partial Zn leaching, we next evaluated how these structural perturbations translate into CO_2_ adsorption. CO_2_ uptake at parts per million-level concentrations was therefore evaluated using a combination of equilibrium adsorption and dynamic breakthrough measurements. This combined approach enables the evaluation of both intrinsic adsorption capacity and transport-limited behavior under flow conditions. Moreover, uptake, breakthrough/TPD, and regeneration measurements are used to build a mechanistic picture of how ligand-mediated defects reshape CO_2_ adsorption and transport, rather than simply to benchmark improved capture performance.

As shown by the static adsorption curves and the bar plots (Figures 3a and S25), both modified samples adsorb substantially more CO_2_ than pristine NICS-24 at low concentrations. Specifically, despite the decrease of total capacity from 2.8 to ∼2.2 mmol/g, the equilibrium uptake at 1000 ppm increases from 0.37 mmol/g for NICS-24 to 0.76 mmol/g for 40FMeTz and 0.77 mmol/g for 60SMeTz, corresponding to more than 100% enhancement at ppm-level CO_2_. The overall isotherm shapes remain similar across all samples, indicating that LMDE does not introduce new adsorption mechanism or generate mesoporosity, but increases the fraction of accessible ultramicropore volume and/or the density of effective binding sites. Accordingly, the enhanced low-pressure CO_2_ uptake is attributed to more effective utilization of available adsorption sites enabled by defect formation, rather than to pore enlargement or intrinsically stronger binding interactions. This interpretation is consistent with the retained Type I isotherm shape, the modest pore-size changes observed by PALS, and the spectroscopic evidence for altered local CO_2_ environments.

**Figure 3:**
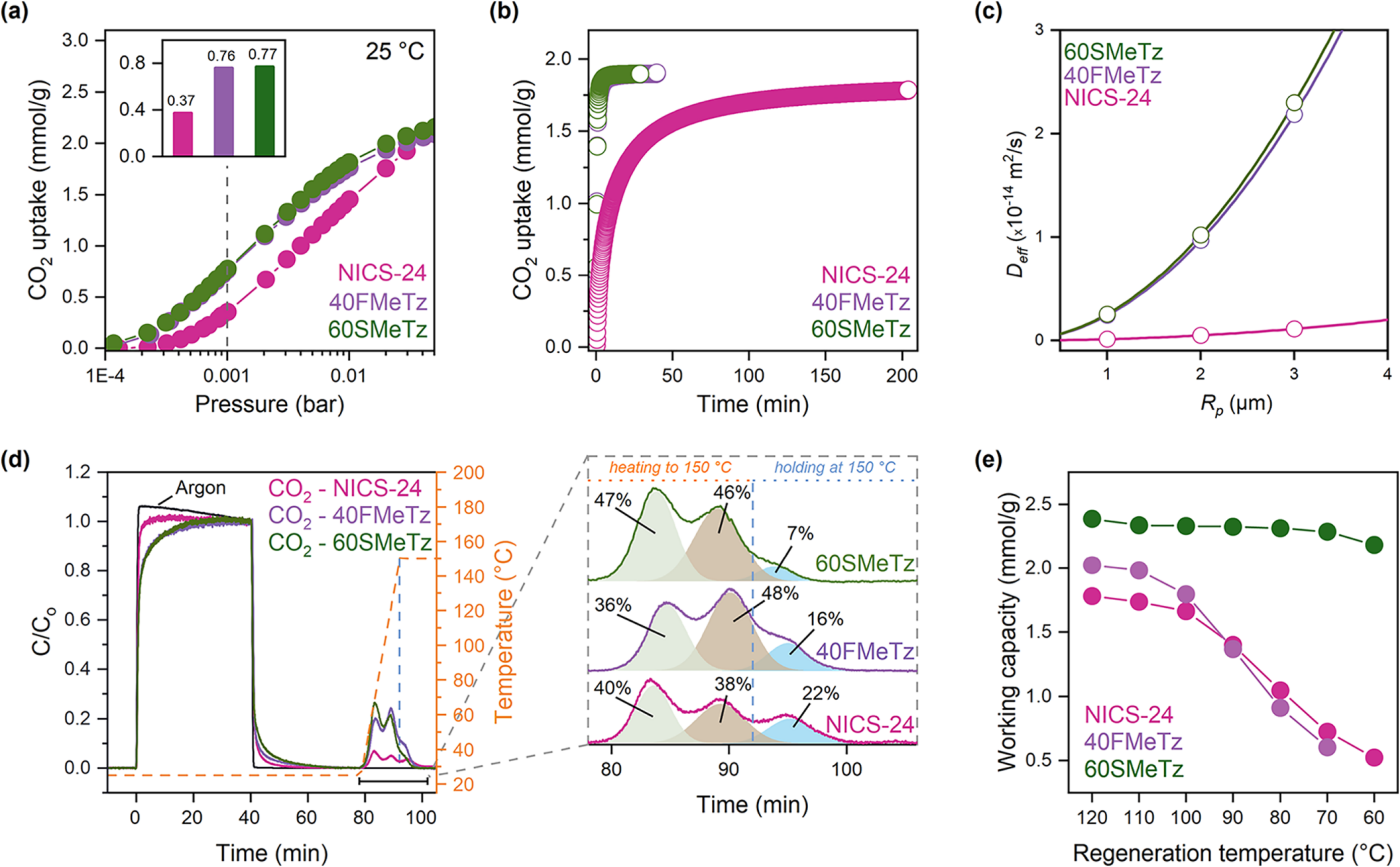
(a) CO_2_ adsorption isotherms at the low-concentration pressure region of pristine NICS-24, 40FMeTz, and 60SMeTz at 25 °C. Inset: comparison of CO_2_ uptakes between all three samples at indoor air capture-relevant concentration (1000 ppm). (b) Adsorption kinetics under dynamic conditions (N_2_ flow at 25 °C) with loading up to 1 bar of CO_2_ relative pressure. (c) Effective CO_2_ diffusivities (*D*_*eff*_) of the studied adsorbents at 0.95 bar and 25 °C, calculated using the LDF model for crystal sizes determined from SEM imaging. (d) Isothermal CO_2_ breakthrough curves of investigated materials followed with the temperature-programmed desorption experiment with temperature ramp (orange dashed line) reaching the plateau at 150 °C (vertical blue dashed line). TPD profiles with the deconvolution and corresponding areas are shown in an inset. (e) Effect of regeneration temperature (120–60 °C) on CO_2_ TSA working capacity.

To exclude solvent-induced activation as the origin of the enhanced CO_2_ uptake, pristine NICS-24 was subjected to identical methanol/water treatment in the absence of FMeTz and SMeTz linkers. Although the methanol-treated material initially shows elevated CO_2_ uptake comparable to 40FMeTz (1.5 mmol/g), this enhancement returns to the level of untreated NICS-24 after 1 week of ambient pressure exposure. In contrast, both 40FMeTz and 60SMeTz retained enhanced uptake after air exposure. This behavior indicates that the effect in pristine NICS-24 arises from a reversible activation process, such as temporary pore opening or removal of residual species, rather than permanent structural modification. In contrast, LMDE provides a durable enhancement by introducing permanent changes in pore connectivity and adsorption site distribution (Figure S26).

To evaluate the impact of LMDE on adsorption dynamics under flow, uptake profiles were monitored from pure N_2_ flow to pure CO_2_, generating an increase of relative pressure from 0 to 1 bar (Figures 3b and S27). The differences in the approach to equilibrium are apparent. Pristine NICS-24 exhibits gradual saturation, reflecting diffusion-limited transport within its one-dimensional ultramicroporous channels. In contrast, both LMDE-modified materials show markedly steeper initial uptake slopes and significantly shorter times to reach near-equilibrium loading. Quantitatively, the time required to reach 95% of equilibrium uptake (*t*_95_, Figure S27) decreases dramatically from 92 min for NICS-24 to 3.0 and 2.6 min for 40FMeTz and 60SMeTz, respectively. In spite of higher final uptakes, both modified materials reach saturation significantly faster than that of pristine NICS-24. Accelerated saturation under flow indicates that ligand-mediated defect formation significantly improves mass-transport pathways and reduces diffusion resistances that are evident in the parent framework.^55^ The pronounced difference in accessibility plays a dominant role in governing the adsorption kinetics under operationally relevant flow conditions.

To further quantify the impact of LMDE on mass transport, effective intraparticle diffusivities (*D*_eff_) were estimated from time-resolved adsorption data (Figure S28). Among the tested kinetic models, the Avrami model provided the best description of the uptake curves (Table S6). When fitted to the diffusion-relevant regime (*C*/*C*_o_ ≤ 0.8), pristine NICS-24 exhibits an Avrami exponent of *n* ∼ 0.62, characteristic of strongly heterogeneous, diffusion-limited transport. In contrast, LMDE-modified materials display significantly higher exponents (*n* ∼ 2.7–2.8), indicative of accelerated and more cooperative uptake behavior associated with improved pore connectivity.^56^ For quantitative comparison, apparent diffusivities were estimated using the linear driving force (LDF) model.^57^ The extracted rate constants (*k*_LDF_) were converted to effective intraparticle diffusivities using a cylindrical particle model (α ∼ 8) an particle radii of 1–3 μm obtained from SEM analysis.^58^ The resulting *D*_eff_ values (Figures 3c and S29) reveal an approximately 1 order of magnitude (10–20×) increase for both modified materials compared to pristine NICS-24. Although the LDF-derived should be regarded as apparent transport parameters, their approximately one-order-of-magnitude increase after LMDE strongly supports the proposed vacancy-mediated transport model, in which Zn-leaching-induced defects reduce diffusion resistance by creating additional pathways through the ultramicroporous framework.

Dynamics of CO_2_ adsorption were further examined by dynamic breakthrough experiments (Figure 3d). Upon switching from a pure Ar carrier to a 1% CO_2_/Ar stream (50 mL/min), all materials progressively approach a normalized equilibrium signal (*C*/*C*_o_ = 1, where *C*_o_ is defined from a steady-state plateau). Pristine NICS-24 reaches the apparent saturation level most rapidly, whereas both modified materials exhibit a more gradual increase, consistent with their higher overall CO_2_ uptake and short channeling (the presence of defect created shortcut routes). Subsequent thermal programmed desorption (TPD) was initiated by ramping the temperature to 150 °C under Ar flow. The total amount of CO_2_ released during TPD is substantially higher for the modified materials (0.69 mmol/g for 40FMeTz and 0.66 mmol/g for 60SMeTz) compared to pristine NICS-24 (0.22 mmol/g), which is in good agreement with equilibrium uptake trends. Deconvolution of TPD traces (Figure 3d (inset)) resolves three distinct desorption contributions which can be attributed to (i) a low-temperature, weakly bound or readily accessible CO_2_, (ii) a medium-temperature moderately strong or confinement-enhanced adsorption environment, and (iii) delayed, diffusion-limited desorption from constricted pathways.^59,60^ For 60SMeTz, the desorption profile is dominated by the first two contributions (47% and 46%, respectively), while the delayed component accounts for only ∼7% of the total release, indicating the presence of mainly unobstructed desorption pathways. In contrast, 40FMeTz and pristine NICS-24 exhibit substantially larger fractions of delayed desorption (16% and 22%, respectively), reflecting a higher population of kinetically hindered domains. These observations further suggest that adsorption in NICS-24 and its modified analogues is strongly influenced by transport phenomena. In this context, the extraction of isosteric heat of adsorption (*Q*_*s*t_) is limited due to the presence of diffusion-limited regimes, where equilibrium conditions are not fully established (Figure S30). As a result, apparent temperature-dependent uptake trends may reflect enhanced diffusivity rather than intrinsic adsorption energetics.

To assess the practical implications of the distinct adsorption kinetics and binding strength, we next examined how the differences translate into thermal-swing adsorption (TSA) regeneration behavior and working capacity as a function of desorption temperature (Figure 3e). The 60SMeTz exhibits the most regeneration-efficient response. Its working capacity decreases by only ∼3% (from 2.4 mmol/g to 2.3 mmol/g) when the regeneration temperature is lowered from 150 to 70 °C. Only at lower temperatures, the capacity starts to drop more notably. This behavior is fully consistent with the TPD analysis, where 60SMeTz shows dominating low- and medium-temperature release processes. Together, these findings indicate that CO_2_ is stored on sites that are both accessible and readily regenerable, enabling efficient cycling under mild thermal input. In contrast, both pristine NICS-24 and 40FMeTz display a strong dependence of working capacity on regeneration temperature. Although their capacities remain relatively high at 100–120 °C, they decrease steeply upon lowering the regeneration temperature below 100 °C, reaching ∼70% reduction at 60 °C. This trend mirrors the TPD results, where a significant fraction of CO_2_ (∼22% for NICS-24 and ∼16% for 40FMeTz) is associated with delayed, high-temperature desorption. The persistence of this slow-desorbing fraction indicates the presence of diffusion-limited or confinement-enhanced domains that require higher thermal input to fully regenerate. Thus, regeneration behavior is governed not only by the overall defect density but also by the spatial distribution and connectivity of defect-associated adsorption domains, which determine whether faster CO_2_ uptake is accompanied by equally efficient desorption.

In summary, the kinetic and regeneration data reveal three transport binding regimes across the sample series. Pristine NICS-24 exhibits relatively slow adsorption under flow and pronounced delayed desorption during TSA, consistent with diffusion limitations within ultramicroporous channels with no defect-mediated transport shortcuts. In contrast, 40FMeTz shows accelerated adsorption but incomplete regeneration at mild temperatures, indicating that defect formation enhances accessibility and improves mass transfer within the framework but at the same time simultaneously introduces stronger and more confined binding domains that require higher thermal input for full desorption. Such an asymmetric adsorption–desorption behavior has been reported for defect-engineered frameworks, where increased site heterogeneity broadens the energy landscape and slows release despite faster uptake kinetics.^46,61^ 60SMeTz achieves both rapid adsorption and efficient desorption, indicating a more optimal redistribution of adsorption sites in which accessibility is improved without generating a significant population of slow-desorbing domains. This balanced transport-interaction interplay explains the superior TSA operability of 60SMeTz and demonstrates how controlled defect engineering can decouple the kinetic acceleration from desorption hindrance.

The long-term cycling stability of the modified materials was evaluated under repeated TSA conditions using alternating adsorption and regeneration steps (Figures S31–S34). All samples exhibit highly reproducible uptake and release profiles over 10 consecutive cycles, with no observable drift in adsorption capacity or regeneration behavior, indicating stable and fully reversible CO_2_ capture. The almost complete retention over all cycles, together with unchanged positions and intensity of XRD peaks (Figure S35), highlights robustness of the modified frameworks.

Resistance of the material toward exposure to water and performance in humid conditions was also evaluated. Water adsorption isotherms of the modified NICS-24 samples were measured at 25 °C (Figure S36). Both modified materials showed decreased total water uptake compared to the parent NICS-24 (4.1 mmol/g), reaching 3.86 mmol/g for the 40FMeTz-modified sample and 3.98 mmol/g for the 60SMeTz modified sample. PXRD analysis performed after soaking the materials in liquid water at different temperatures (40, 70, and 100 °C) shows no visible changes in diffraction patterns (Figures S37, S38, and S39), indicating that the NICS-24 framework and its modified derivatives retain their crystallinity and structural integrity upon prolonged exposure to water. Despite this high hydrolytic stability, CO_2_ capture performance under humid conditions is strongly suppressed. At 50% RH, all samples retain only ∼10% of their dry CO_2_ uptake, confirming that competitive adsorption of water remains dominant in the pore environment (Figure S40). This behavior is consistent with the LMDE mechanism. The postsynthetic treatment does not lead to significant incorporation of CF_3_- or SCH_3_-functionalized ligands but instead predominantly generates metal-deficient defects via partial Zn leaching. As a result, the pore environment remains largely unchanged and retains its hydrophilic character, governed by polar NH_2_ functions. Even though modified materials are structurally robust toward moisture, their application to wet gas streams would require upstream drying or further hydrophobic modification to mitigate water interference.

### Atomistic Insights into CO_2_ Sorption Mechanism

While macroscopic capture experiments reveal clear differences in uptake capacity, kinetics, and regenerability, at this stage, the CO_2_ binding mechanism remains unresolved. Therefore, atomistic insight into the sorption mechanism was performed using in situ DRIFTS and ex situ solid-state NMR upon CO_2_ loading on investigated samples.

The broad IR bands at 2360 and 2340 cm^–1^ (Figure S41) correspond to asymmetric stretching (ν_3_) mode of gas-phase CO_2_. Upon exposure to 1000 ppm of CO_2_, all samples exhibit pronounced spectral changes, confirming adsorption within the framework even under dilute conditions. The spectra are dominated by a band centered at ∼2343 cm^–1^, assigned to monomeric CO_2_ interacting with adsorption sites in the framework. Notably, the intensity of this band follows the trend 60SMeTz > 40FMeTz ≫ NICS-24, in agreement with the enhanced CO_2_ uptake observed in isothermal measurements at 1000 ppm (Figures 4a and S41). The ν_3_ band exhibits clear asymmetry toward lower wavenumbers, with a pronounced shoulder at ∼2330 cm^–1^ that is significantly more developed in the modified samples. This feature is attributed to weakly interacting or confined CO_2_ species, including pore-confined or dimer-like configurations within ultramicropores.^62^ Deconvolution of the ν_3_ band reveals that the integrated area of the ∼2330 cm^–1^ component is significantly enhanced in the modified materials, being approximately 15-fold higher for 60SMeTz and 9-fold higher for 40FMeTz compared to pristine NICS-24 (Figures 4a (inset) and S42). This pronounced increase may reflect enhanced CO_2_–CO_2_ interactions within the pores, facilitated by increased accessible pore volume and improved diffusion pathways arising from defect formation.

**Figure 4:**
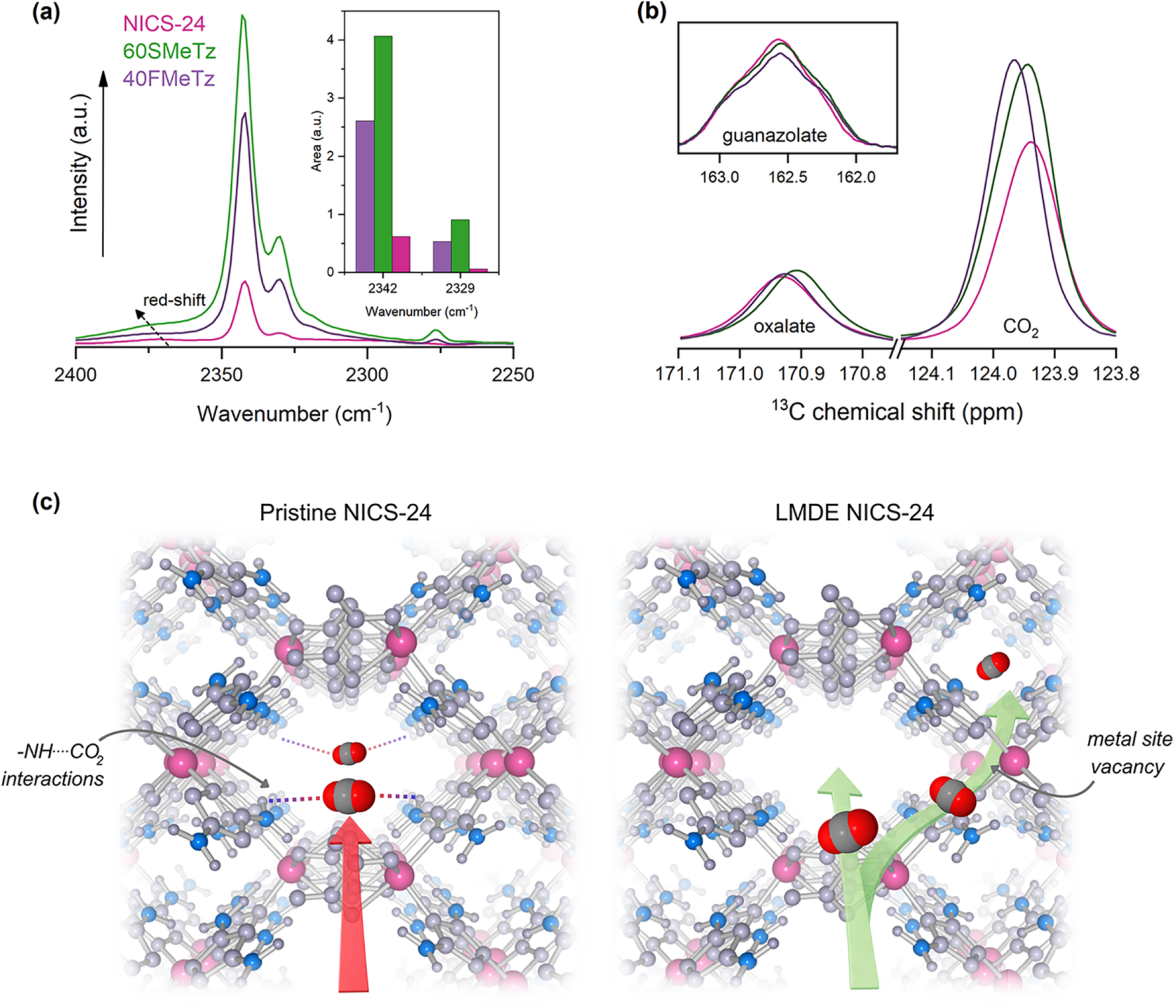
(a) DRIFTS spectra showing relevant IR bands of specific CO_2_ species adsorbed on investigated materials exposed to 1000 ppm of CO_2_ in N_2_ flow. Inset shows integrated values from deconvolution of the bands corresponding to adsorbed monomeric CO_2_ (∼2343 cm^–1^) and CO_2_–CO_2_ dimers (∼2330 cm^–1^). (b) ^1^H–^13^C CP-MAS spectra of individual CO_2_-loaded samples, revealing a systematic shift of the physisorbed CO_2_ resonance, consistent with different averaged physisorption environments for CO_2_ across the series. (c) Proposed dynamics of CO_2_ adsorption in NICS-24 (left) and LMDE-modified (right) frameworks. In pristine material, the CO_2_ diffusion is hindered by host–guest interactions combined with confinement within 1D ultramicropore channels, which leads to kinetic barriers to transport (red arrow). LMDE, on the other hand, introduces metal-node vacancies that establish alternative migration pathways, facilitating faster mass transport and improves sorption kinetics (green arrow).

In addition, a weak high-frequency contribution extending toward ∼2380 cm^–1^ is observed, particularly in the 60SMeTz sample. This blue-shifted component is assigned to CO_2_ interacting with polarized adsorption sites, most likely associated with metal-based centers, where electrostatic interactions (Stark effect) lead to an increase in the ν_3_ frequency.^63^ The relatively small magnitude of this shift and its low intensity suggest that these sites represent a minority population but contribute to broader distribution of adsorption sites within the modified samples.^64,65^

To elucidate the molecular-level interactions governing CO_2_ adsorption in the defect-engineered materials, SSNMR experiments were performed on samples loaded with ^13^CO_2_ using a laboratory-built gas-dosing manifold that enables evacuation, controlled dosing, and sealing of MAS rotors under inert conditions (Figure S43). Upon evacuation and subsequent CO_2_ loading, systematic changes are observed in the ^1^H MAS spectra of 60SMeTz (Figure S44a). In agreement with previous studies, the water resonance shifts from δ ≈ 2.68 ppm in the fully hydrated material to δ ≈ 1.91 ppm after evacuation and to δ ≈ 2.16 ppm after CO_2_ loading, reflecting changes in hydrogen-bonding interactions involving confined H_2_O molecules.^48^ Correspondingly, the oxalate ^13^C resonance exhibits a slight shift upon CO_2_ loading (Figure S44b), indicating modified local interactions involving residual H_2_O and CO_2_. In addition, the guanazolate ^13^C resonance of CO_2_-loaded 60SMeTz undergoes a small upfield shift accompanied by pronounced line broadening, which is attributed to the formation of O = C=O···H–N hydrogen bonds between CO_2_ and framework NH_2_ groups. Such interactions restrict local motion and give rise to increased inhomogeneous broadening of the resonance.

A distinct ^13^C resonance at δ ≈ 124 ppm accompanied by pronounced spinning sidebands appears in the ^1^H–^13^C CP-MAS spectra of CO_2_-loaded samples (Figures S44c and Figure 4b), confirming the presence of physisorbed CO_2_ confined within the micropores of NICS-24-based frameworks.^48^ No additional ^13^C resonances attributable to chemisorbed species are observed, indicating that CO_2_ uptake proceeds via reversible physisorption. Comparison of the ^1^H–^13^C CP-MAS spectra of individual CO_2_-loaded samples reveals a systematic shift of the physisorbed CO_2_ resonance across the series (Figure 4b): CO_2_ in NICS-24 appears at the lowest chemical shift, followed by 60SMeTz, while 40FMeTz exhibits the most downfield position. Recent NMR studies on charged and polar sorbents have shown that strongly physisorbed CO_2_ in pore environments resonates upfield relative to free CO_2_ gas (δ ≈ 125 ppm), whereas interactions with electropositive sites induce downfield shifts.^66^ In the present system, the observed shifts are upfield of free CO_2_ gas across all samples, consistent with electron-rich amine functionalities providing a net-shielding environment; the small differences between samples reflect variations in the distribution of CO_2_ adsorption sites and/or changes in their local environments.

Low-temperature SSNMR measurements further support the presence of multiple CO_2_ adsorption environments. At −25 °C, the physisorbed CO_2_ resonance resolves into two components, whereas a single averaged resonance is observed at room temperature, consistent with fast exchange between distinct binding sites on the NMR time scale at higher temperature.^68^ The low-temperature spectra reveal a dominant upfield-shifted component relative to the room-temperature resonance (Figure S45). This behavior reflects restricted molecular motion at lower temperature and increased residence time at stronger binding sites.^67^ A ^1^H–^13^C CP-HETCOR spectrum of CO_2_-loaded 60SMeTz recorded at −25 °C (Figure S46) shows correlations exclusively with framework NH_2_ protons, with no additional ^1^H resonances observed, indicating that the same guanazolate NH_2_ groups dominate CO_2_ binding across both dynamic and immobilized regimes. Collectively, the SSNMR results support vacancy-type defect formation and reveal material-dependent physisorbed CO_2_ environment.

Integration of spectroscopic and sorption analyses enables us to propose a diffusion-controlled model that rationalizes the performance evolution through the LMDE process (Figure 4c). In pristine NICS-24, CO_2_ migration through 1D channels is strongly coupled to host–guest interactions, resulting in confinement-enhanced residence times and diffusion-limiting domains. This manifests slower adsorption kinetics, pronounced delayed desorption in the TPD regime, and a reduced regeneration efficiency at mild temperatures. LMDE partially relaxes this diffusion-binding coupling by introducing Zn-deficient sites that modify the pore accessibility and connectivity. The resulting enhancement in mass transport is reflected in the faster adsorption kinetics, reduced delayed desorption, and improved regeneration behavior observed for the modified materials.

The performance improvements do not arise simply from defect generation, but rather from how these defects reshape the distribution and connectivity of the resulting adsorption environments. While both 40FMeTz and 60SMeTz exhibit comparable levels of Zn deficiency, their adsorption–desorption behaviors differ. The 60SMeTz sample displays a more favorable distribution of accessible adsorption environments that enables both efficient CO_2_ uptake and facile release, whereas 40FMeTz retains a larger fraction of kinetically hindered domains. Thus, the key parameter governing performance is not defect density alone but the connectivity and accessibility of the adsorption environments generated through LMDE.

Overall, LMDE enhances performance by coupling diffusion from adsorption; however, optimal behavior is achieved only when defect formation promotes continuous accessibility of sorption sites without generating a significant population of isolated or kinetically hindered domains, as exemplified by 60SMeTz.

## Conclusions

Ligand-mediated defect engineering (LMDE) of NICS-24 with functionalized azolate ligands induces partial Zn leaching while preserving long-range crystallinity and topology. The treated samples exhibit subtle unit-cell contraction accompanied by systematic XRD peak broadening, consistent with increased local disorder. PALS further reveals a notable expansion of the micropore free volume, indicating that defect formation perturbs the local pore environment. These structural modifications translate directly into improved CO_2_ capture performance under indoor-relevant conditions. The defect-engineered materials display more than 2-fold increase of uptake at 25 °C and 1000 ppm (from 0.37 mmol/g for pristine NICS-24 to 0.76 mmol/g and 0.77 mmol/g for 40FMeTz and 60SMeTz samples respectively), accompanied by substantially faster uptake dynamics under flow conditions.

The increased adsorption capacity is enhanced without compromising regeneration behavior. In particular, 60SMeTz combines high affinity at low CO_2_ partial pressures with rapid adsorption and efficient thermal release. Consequently, the material maintains nearly constant working capacity at regeneration temperatures as low as 70 °C and exhibits stable, fully reversible TSA cycling. The combination of higher uptake, shorter equilibrium time, and mild-temperature regenerability results in a markedly improved operational working capacity under realistic thermal-swing conditions compared with the pristine framework.

Spectroscopic analysis using DRIFTS and SSNMR further confirms that CO_2_ adsorption remains reversible with LMDE primarily modifying the local hydrogen-bonding environment and distribution of adsorption domains. These defect-induced perturbations introduce additional diffusion pathways. Although competitive water adsorption suppresses the performance under humid conditions, the framework remains hydrolytically robust, providing a stable platform for further hydrophobic tuning.

Overall, this study demonstrates that LMDE can convert a strongly adsorbing but diffusion-limited ultramicroporous MOF into a kinetically efficient adsorbent for dry, low-concentration CO_2_ capture. The combination of enhanced ppm-level CO_2_ uptake, rapid adsorption kinetics, and low-temperature regenerability positions LMDE-modified NICS-24 as a highly efficient physisorptive MOF platform for low-concentration CO_2_ capture, comparable to benchmark materials (Figure S47 and Table S7). The results point up how controlled defect formation can reshape the diffusion behavior in confined porous solids, offering a general pathway to overcome kinetic and energetic limitations that often constrain the real-world deployment of adsorbents for ambient carbon capture applications.

## Supplementary Material



## Notes

Experimental details and synthesis, structural and spectroscopic characterization (PXRD, SEM-EDS, XPS, solution-state and solid-state NMR, DRIFTS); gas sorption measurements (CO_2_, N_2_, and H_2_O isotherms), BET and NLDFT pore-size analysis, TGA-DTG data; CO_2_ adsorption kinetics and diffusivity analysis; evaluation of working capacities, thermal-swing adsorption (TSA) cycling stability, and heat of adsorption; breakthrough measurements under humid conditions (50% RH, 1000 ppm of CO_2_); and comparison with reported MOFs for low-concentration CO_2_ capture (PDF)

## References

[1] Powell J. A., Yang Y., Zhou H. C. (2025). Design, Analysis, and Application
of Metal–Organic Framework Derived Carbons. Inorg. Chem. Front..

[2] Zhang J. P., Zhou H. L., Zhou D. D., Liao P. Q., Chen X. M. (2018). Controlling
Flexibility of Metal–Organic Frameworks. Natl. Sci. Rev..

[3] Zeggai F. Z., Ait-Touchente Z., Bachari K., Elaissari A. (2025). Investigation
of Metal-Organic Frameworks (MOFs): Synthesis, Properties, and Applications
- An In-Depth Review. Chem. Phys. Impact.

[4] Wang Y. M., Lin J. T., Ning G. H., Li D. (2025). Recent Advances in
Metal–Organic Frameworks for Catalysing Organic Transformation. Chem. Commun..

[5] Li Z., Yao B., Cheng C., Song M., Qin Y., Wan Y., Du J., Zheng C., Xiao L., Li S., Yin P. F., Guo J., Liu Z., Zhao M., Huang W. (2024). Versatile Structural
Engineering of Metal–Organic Frameworks Enabling Switchable
Catalytic Selectivity. Adv. Mater..

[6] Furukawa H., Cordova K. E., O’Keeffe M., Yaghi O. M. (2013). The Chemistry and
Applications of Metal-Organic Frameworks. Science.

[7] Li D., Yadav A., Zhou H., Roy K., Thanasekaran P., Lee C. (2024). Advances and Applications of Metal-Organic
Frameworks (MOFs) in Emerging
Technologies: A Comprehensive Review. Glob.
Chall..

[8] Yusuf V. F., Malek N. I., Kailasa S. K. (2022). Review
on Metal–Organic Framework
Classification, Synthetic Approaches, and Influencing Factors: Applications
in Energy, Drug Delivery, and Wastewater Treatment. ACS Omega.

[9] Abdul-Wahab S. A., Wahab S. A., En S. C. F., Elkamel A., Ahmadi L., Yetilmezsoy K. (2015). A Review of
Standards and Guidelines Set by International
Bodies for the Parameters of Indoor Air Quality. Atmos. Pollut. Res..

[10] Li W. L., Shuai Q., Yu J. (2024). Recent Advances of Carbon Capture
in Metal–Organic Frameworks: A Comprehensive Review. Small.

[11] Sher F., Hayward A., El Guerraf A., Wang B., Ziani I., Hrnjić H., Boškailo E., Chupin A., Nemtanu M. R. (2024). Advanced
Metal–Organic Frameworks for Superior Carbon Capture, High-Performance
Energy Storage and Environmental Photocatalysis – a Critical
Review. J. Mater. Chem. A.

[12] Sumida K., Rogow D. L., Mason J. A., McDonald T. M., Bloch E. D., Herm Z. R., Bae T. H., Long J. R. (2012). Carbon Dioxide Capture
in Metal–Organic Frameworks. Chem. Rev..

[13] Xie Y., Cui H., Wu H., Lin R. B., Zhou W., Chen B. (2021). Electrostatically
Driven Selective Adsorption of Carbon Dioxide over Acetylene in an
Ultramicroporous Material. Angew. Chem., Int.
Ed..

[14] Ding M., Rong W., Wang Y., Kong S., Yao J. (2023). Pore Engineering
of Metal–Organic Frameworks for Boosting Low-Pressure CO_2_ Capture. J. Mater. Chem. A.

[15] Cai S., Yu L., Huo E., Ren Y., Liu X., Chen Y. (2024). Adsorption
and Diffusion Properties of Functionalized MOFs for CO_2_ Capture: A Combination of Molecular Dynamics Simulation and Density
Functional Theory Calculation. Langmuir.

[16] Yadav A. K., Gładysiak A., Song A. Y., Gan L., Simons C. R., Alghoraibi N. M., Alahmed A. H., Younes M., Reimer J. A., Huang H., Planas J. G., Stylianou K. C. (2024). Sequential
Pore Functionalization in MOFs for Enhanced Carbon Dioxide Capture. JACS Au.

[17] Zhang G., Zhao P., Xu Y., Yang Z., Cheng H., Zhang Y. (2018). Structure Property–CO_2_ Capture Performance Relations
of Amine-Functionalized Porous Silica Composite Adsorbents. ACS Appl. Mater. Interfaces.

[18] Hui Q., Fu J., Xu H., Wang C., Ding Q., Zhu W., Sun J., Zhou Z., Wu Y., Hu X., Zhang Z. (2025). Metal–Organic
Frameworks for CO_2_ Capture: Challenges and Efforts from
Laboratory Research to Industrial Applications. Ind. Eng. Chem. Res..

[19] Qiu X., Wang R. (2025). From Construction Strategies
to Applications: Multifunctional Defective
Metal-Organic Frameworks. Coord. Chem. Rev..

[20] Xiang W., Zhang Y., Chen Y., Liu C. J., Tu X. (2020). Synthesis,
Characterization and Application of Defective Metal–Organic
Frameworks: Current Status and Perspectives. J. Mater. Chem. A.

[21] Tu B., Pang Q., Wu D., Song Y., Weng L., Li Q. (2014). Ordered Vacancies and
Their Chemistry in Metal-Organic Frameworks. J. Am. Chem. Soc..

[22] Fang Z., Bueken B., De Vos D. E., Fischer R. A. (2015). Defect-Engineered
Metal–Organic Frameworks. Angew. Chem.,
Int. Ed..

[23] Hou X., Wang J., Mousavi B., Klomkliang N., Chaemchuen S. (2022). Strategies for Induced Defects in Metal–Organic
Frameworks for Enhancing Adsorption and Catalytic Performance. Dalton Trans..

[24] Szilágyi P. Á., Serra-Crespo P., Gascon J., Geerlings H., Dam B. (2016). The Impact of Post-Synthetic
Linker Functionalization of MOFs on
Methane Storage: The Role of Defects. Front.
Energy Res..

[25] Karagiaridi O., Vermeulen N. A., Klet R. C., Wang T. C., Moghadam P. Z., Al-Juaid S. S., Stoddart J. F., Hupp J. T., Farha O. K. (2015). Functionalized
Defects through Solvent-Assisted Linker Exchange: Synthesis, Characterization,
and Partial Postsynthesis Elaboration of a Metal-Organic Framework
Containing Free Carboxylic Acid Moieties. Inorg.
Chem..

[26] Fu Y., Yao Y., Forse A. C., Li J., Mochizuki K., Long J. R., Reimer J. A., De Paëpe G., Kong X. (2023). Solvent-Derived Defects Suppress Adsorption in MOF-74. Nat. Commun..

[27] Zhang L., Wang J., Wang H., Zhang W., Zhu W., Du T., Ni Y., Xie X., Sun J., Wang J. (2021). Rational Design
of Smart Adsorbent Equipped with a Sensitive Indicator via Ligand
Exchange: A Hierarchical Porous Mixed-Ligand MOF for Simultaneous
Removal and Detection of Hg^2+^. Nano
Res..

[28] Schulz M., Marquardt N., Schäfer M., Heinemeyer T., Schaate A. (2020). Solvent-Assisted Linker Exchange
as a Tool for the
Design of Mixed-Linker MIL-140D Structured MOFs for Highly Selective
Detection of Gaseous H_2_S. RSC Adv..

[29] Taddei M., Wakeham R. J., Koutsianos A., Andreoli E., Barron A. R. (2018). Post-Synthetic
Ligand Exchange in Zirconium-Based Metal–Organic Frameworks:
Beware of The Defects!. Angew. Chem..

[30] Marreiros J., Caratelli C., Hajek J., Krajnc A., Fleury G., Bueken B., De Vos D. E., Mali G., Roeffaers M. B. J., Van Speybroeck V., Ameloot R. (2019). Active Role of Methanol
in Post-Synthetic Linker Exchange in the Metal-Organic Framework UiO-66. Chem. Mater..

[31] Feng X., Jena H. S., Krishnaraj C., Arenas-Esteban D., Leus K., Wang G., Sun J., Rüscher M., Timoshenko J., Roldan Cuenya B., Bals S., Van Der
Voort P. (2021). Creation of Exclusive Artificial Cluster Defects by Selective Metal
Removal in the (Zn, Zr) Mixed-Metal UiO-66. J. Am. Chem. Soc..

[32] Sannes D. K., Øien-ØDegaard S., Aunan E., Nova A., Olsbye U. (2023). Quantification of Linker Defects in UiO-Type Metal-Organic
Frameworks. Chem. Mater..

[33] Jajko G., Calero S., Kozyra P., Makowski W., Sławek A., Gil B., Gutiérrez-Sevillano J. J. (2022). Defect-Induced
Tuning of Polarity-Dependent
Adsorption in Hydrophobic–Hydrophilic UiO-66. Commun. Chem..

[34] Denny M. S., Parent L. R., Patterson J. P., Meena S. K., Pham H., Abellan P., Ramasse Q. M., Paesani F., Gianneschi N. C., Cohen S. M. (2018). Transmission Electron Microscopy Reveals Deposition
of Metal Oxide Coatings onto Metal-Organic Frameworks. J. Am. Chem. Soc..

[35] Niu J., Li H., Tao L., Fan Q., Liu W., Tan M. C. (2023). Defect
Engineering of Low-Coordinated Metal-Organic Frameworks (MOFs) for
Improved CO_2_ Access and Capture. ACS Appl. Mater. Interfaces.

[36] Yan W., Hou J., Yan T., Liu Z., Kang P. (2025). Amine-Functionalized
Defective MOFs for Direct Air Capture by Postsynthetic Modification. ACS Appl. Mater. Interfaces.

[37] Dong H., Li L. H., Feng Z., Wang Q. N., Luan P., Li J., Li C. (2023). Amine-Functionalized
Quasi-MOF for Direct Air Capture
of CO_2_. ACS Mater. Lett..

[38] Wang B., Ying P., Zhang J. (2023). Effects of Missing Linker Defects
on the Elastic Properties and Mechanical Stability of the Metal-Organic
Framework HKUST-1. J. Phys. Chem. C.

[39] Evangelou D. A., Makri E. C., Pliatsios N., Vamvasakis I., Buchsteiner E., Oikonomopoulos P., Armatas G. S., Papaefstathiou G. S., Lazarides T., Manos M. J. (2025). Ultramicroporous Al­(III) MOFs with
Selective CO_2_ Adsorption, Acid Resistance, and Efficient
Cr­(VI) Sorption Properties. Dalton Transact..

[40] Kim K. C., Yoon T. U., Bae Y. S. (2016). Applicability
of Using CO_2_ Adsorption Isotherms to Determine BET Surface
Areas of Microporous
Materials. Micropor. Mesopor. Mater..

[41] Ambroz F., Macdonald T. J., Martis V., Parkin I. P. (2018). Evaluation of the
BET Theory for the Characterization of Meso and Microporous MOFs. Small Methods.

[42] Becker T. M., Lin L. C., Dubbeldam D., Vlugt T. J. H. (2018). Polarizable Force
Field for CO_2_ in M-MOF-74 Derived from Quantum Mechanics. J. Phys. Chem. C.

[43] Islamoglu T., Idrees K. B., Son F. A., Chen Z., Lee S. J., Li P., Farha O. K. (2021). Are You
Using the Right Probe Molecules for Assessing
the Textural Properties of Metal–Organic Frameworks?. J. Mater. Chem. A.

[44] Attallah A. G., Bon V., Maity K., Zaleski R., Hirschmann E., Kaskel S., Wagner A. (2024). Revisiting
Metal-Organic Frameworks
Porosimetry by Positron Annihilation: Metal Ion States and Positronium
Parameters. J. Phys. Chem. Lett..

[45] Vaidhyanathan, R. ; Nandi, S. ; De Luna, P. ; Daff, T. D. ; Rother, J. ; Liu, M. ; Buchanan, W. ; Woo, T. K. ; Hawari, A. I. A Single-Ligand Ultra-Microporous MOF for Precombustion CO_2_Capture and Hydrogen Purification; American Association for the Advancement of Science, 2015; Vol. 1(11). 10.1126/sciadv.1500421 PMC4730842 26824055

[46] Chetry S., Lukman M. F., Bon V., Warias R., Fuhrmann D., Möllmer J., Belder D., Gopinath C. S., Kaskel S., Pöppl A., Krautscheid H. (2024). Exploring Defect-Engineered Metal-Organic
Frameworks with 1,2,4-Triazolyl Isophthalate and Benzoate Linkers. Inorg. Chem..

[47] Fang Z., Dürholt J. P., Kauer M., Zhang W., Lochenie C., Jee B., Albada B., Metzler-Nolte N., Pöppl A., Weber B., Muhler M., Wang Y., Schmid R., Fischer R. A. (2014). Structural Complexity in Metal-Organic Frameworks:
Simultaneous Modification of Open Metal Sites and Hierarchical Porosity
by Systematic Doping with Defective Linkers. J. Am. Chem. Soc..

[48] Klemenčič K., Krajnc A., Puškarić A., Huš M., Marinič D., Likozar B., Logar N. Z., Mazaj M. (2025). Amine-Functionalized
Triazolate-Based Metal–Organic Frameworks for Enhanced Diluted
CO_2_ Capture Performance. Angew. Chem.,
Int. Ed..

[49] Xu J., Liu X., Liu X., Yan T., Wan H., Cao Z., Reimer J. A. (2022). Deconvolution
of Metal Apportionment in Bulk Metal-Organic
Frameworks. Sci. Adv..

[50] Cistola D. P., Small D. M., Hamilton J. A. (1982). Ionization Behavior of Aqueous Short-Chain
Carboxylic Acids: A Carbon-13 NMR Study. J.
Lipid Res..

[51] Al-Ani A. J., Szell P. M. J., Rehman Z., Blade H., Wheatcroft H. P., Hughes L. P., Brown S. P., Wilson C. C. (2022). Combining X-Ray
and NMR Crystallography to Explore the Crystallographic Disorder in
Salbutamol Oxalate. Cryst. Growth Des..

[52] Jiang J., Yaghi O. M. (2015). Brønsted Acidity in Metal-Organic Frameworks. Chem. Rev..

[53] Yu D., Shao Q., Song Q., Cui J., Zhang Y., Wu B., Ge L., Wang Y., Zhang Y., Qin Y., Vajtai R., Ajayan P. M., Wang H., Xu T., Wu Y. (2020). A Solvent-Assisted
Ligand Exchange Approach Enables Metal-Organic
Frameworks with Diverse and Complex Architectures. Nat. Commun..

[54] Daliran S., Oveisi A. R., Kung C. W., Sen U., Dhakshinamoorthy A., Chuang C. H., Khajeh M., Erkartal M., Hupp J. T. (2024). Defect-Enabling
Zirconium-Based Metal–Organic Frameworks for Energy and Environmental
Remediation Applications. Chem. Soc. Rev..

[55] Marti R. M., Howe J. D., Morelock C. R., Conradi M. S., Walton K. S., Sholl D. S., Hayes S. E. (2017). CO_2_ Dynamics in Pure and
Mixed-Metal MOFs with Open Metal Sites. J. Phys.
Chem. C.

[56] Shirzad K., Viney C. (2025). Revisiting Time-Dependent Growth
and Nucleation Rates in the Johnson-Mehl-Avrami-Kolmogorov
Equation. R. Soc. Open Sci..

[57] Yu Z., Deschamps J., Hamon L., Karikkethu Prabhakaran P., Pré P. (2017). Hydrogen Adsorption
and Kinetics in MIL-101­(Cr) and
Hybrid Activated Carbon-MIL-101­(Cr) Materials. Int. J. Hydrogen Energy.

[58] Tovar T. M., Zhao J., Nunn W. T., Barton H. F., Peterson G. W., Parsons G. N., LeVan M. D. (2016). Diffusion
of CO_2_ in Large
Crystals of Cu-BTC MOF. J. Am. Chem. Soc..

[59] Maia R. A., Louis B., Gao W., Wang Q. (2021). CO_2_ Adsorption
Mechanisms on MOFs: A Case Study of Open Metal Sites, Ultra-Microporosity
and Flexible Framework. React. Chem. Eng..

[60] Krishna R. (2015). Methodologies
for Evaluation of Metal–Organic Frameworks in Separation Applications. RSC Adv..

[61] Nguyen K. D., Vo N. T., Le K. T. M., Ho K. V., Phan N. T. S., Ho P. H., Le H. V. (2023). Defect-Engineered
Metal–Organic
Frameworks (MOF-808) towards the Improved Adsorptive Removal of Organic
Dyes and Chromium (VI) Species from Water. New
J. Chem..

[62] Mihaylov M., Chakarova K., Andonova S., Drenchev N., Ivanova E., Sabetghadam A., Seoane B., Gascon J., Kapteijn F., Hadjiivanov K. (2016). Adsorption Forms of CO_2_ on MIL-53­(Al) and
NH2-MIL-53­(Al) As Revealed by FTIR Spectroscopy. J. Phys. Chem. C.

[63] Montanari T., Busca G. (2008). On the Mechanism of
Adsorption and Separation of CO_2_ on
LTA Zeolites: An IR Investigation. Vib. Spectrosc..

[64] Sathyanarayana, D. N. Vibrational Spectroscopy: Theory and Applications; New Age International Publishers: New Delhi, 2015.

[65] Bordiga S., Regli L., Bonino F., Groppo E., Lamberti C., Xiao B., Wheatley P. S., Morris R. E., Zecchina A. (2007). Adsorption
Properties of HKUST-1 toward Hydrogen and Other Small Molecules Monitored
by IR. Phys. Chem. Chem. Phys..

[66] Li H., Zick M. E., Trisukhon T., Signorile M., Liu X., Eastmond H., Sharma S., Spreng T. L., Taylor J., Gittins J. W., Farrow C., Lim S. A., Crocellà V., Milner P. J., Forse A. C. (2024). Capturing
Carbon Dioxide from Air
with Charged-Sorbents. Nature.

[67] Masala A., Grifasi F., Atzori C., Vitillo J. G., Mino L., Bonino F., Chierotti M. R., Bordiga S. (2016). CO_2_ Adsorption
Sites in UTSA-16: Multitechnique Approach. J.
Phys. Chem. C.

[68] Inukai M., Kurihara T., Noda Y., Jiang W., Takegoshi K., Ogiwara N., Kitagawa H., Nakamura K. (2020). Probing Dynamics of
Carbon Dioxide in a Metal–Organic Framework under High Pressure
by High-Resolution Solid-State NMR. Phys. Chem.
Chem. Phys..

