# Supplementary material for: Ligand-Mediated Defects Unlock Fast and Regenerable CO2 Capture in NICS-24 Metal–Organic Framework
Source: J Am Chem Soc. 2026 Jun 16;148(25):26005–16. doi: 10.1021/jacs.6c04820 (PMC13339166; doi:10.1021/jacs.6c04820)
Supplement: Supplementary file 1 [file ja6c04820_si_001.pdf]

Supporting information

Ligand-Mediated Defects Unlock Fast and Regenerable CO_2 capture in NICS-24 Metal-Organic Framework

Klara Klemenčič[1,2], Petar Djinović[1,2], Miha Okorn[1,2], Jakob Höfferle[1,3], Andraž Krajnc[1], Durga Acharya[4], Cara M. Doherty[4], Dana Marinič[1,5], Blaž Likozar[1], Nataša Zabukovec Logar[1,2], Matjaž Mazaj[1,]*

[1] National Institute of Chemistry, Hajdrihova ulica 19, 1000, Ljubljana, Slovenia
[2] University of Nova Gorica, Vipavska cesta 13, 5000 Nova Gorica, Slovenia
[3] Faculty of Chemistry and Chemical Technology, University of Ljubljana, Večna pot 113, 1000 Ljubljana, Slovenia
[4] CSIRO, Manufacturing, Clayton, VIC 3168, Australia
[5] Faculty of Chemistry and Chemical Engineering, University of Maribor, Smetanova 17, 2000 Maribor, Slovenia

Table of Contents

EXPERIMENTAL SECTION 1
Synthesis 1
Methods 3
Table S1 10
Figure S1 10
Figure S2 11
Figure S3 11
Figure S4 12
Figure S5 12
Figure S6 13
Figure S7 13
Figure S8 14
Figure S9 14
Table S2 15
Figure S10 15
Figure S11 16
Figure S12 17
Table S3 18
Figure S13 19
Figure S14 19
Figure S15 20
Figure S16 21
Figure S17 22
Figure S18 23
Table S4 23
Figure S19 24
Figure S20 25
Table S5 25
Figure S21 26
Figure S22 27
Figure S23 27
Figure S24 28
Figure S25 28
Figure S26 29

Figure S27 29
Figure S28 30
Table S6 30
Figure S29 31
Figure S30 32
Figure S31 33
Figure S32 33
Figure S33 34
Figure S34 34
Figure S35 35
Figure S36 35
Figure S37 36
Figure S38 36
Figure S39 37
Figure S40 38
Figure S41 39
Figure S42 39
Figure S43 40
Figure S44 40
Figure S45 41
Figure S46 41
Figure S47 42
Table S7 43
REFERENCES 44

EXPERIMENTAL SECTION

Synthesis

NICS-24 (Zn-3,5-diamine-1,2,4-triazolate oxalate) parent framework. NICS-24 was synthesized according to the published data,[1] with slight modifications. The synthesis was conducted via a solvent-assisted ligand exchange (SALE) protocol, selected based on empirical observations indicating that SALE procedure afforded both the highest yield, phase purity and the most rapid formation of the targeted MOF structure in comparison to direct synthesis either from Zn-oxalate or ZnO precursors. Typically, 1.00 g (5.11 mmol) of prepared CALF-20 powder was dispersed in a mixture of 10 ml of methanol (100% Fluka) and 10 ml of demineralized water by stirring at room temperature for 15 minutes. Then 1.40 g (14.13 mmol) of guanazole (98% Aaron Chem) was added to the mixture. After an additional 15 minutes of stirring, the mixture was then transferred to a 50 ml Duran glass bottle and heated in a convection oven at 120 ˚C for 48 h. The obtained white powder was washed with ethanol and then dried in ambient conditions. The synthesis yield was 85% in respect to CALF-20. The crystallinity of the product was confirmed by powder XRD measurement (**Figure S24**).

CALF-20 (Zn-1,2,4-triazolate oxalate). Synthesis of CALF-20 was prepared according to published data.[2] Typically, mixture of 1.32 g (6.97 mmol) of zinc oxalate dihydrate (99% Aaron Chem), 1.00 g (14.48 mmol) of 1,2,4-triazole (97 % Fluorochem) and 13 ml of methanol (100%, Fluka) was heated in 23 ml Parr stainless steel Teflon-lined autoclave at 180 ˚C for 48 h. The product in the form of white powder was recovered by filtration, rinsed with ethanol and dried

in ambient conditions. The synthesis achieved a 91% yield based on the metal precursor, and the quality of the final product was verified using powder XRD analysis. (**Figure S24**).

General procedure for modified NICS-24. The modification was performed stepwise, based on the molar ratio between the desired linker and the parent framework. The approach, schematically presented in **Figure 1a,** was performed using a solvent-assisted ligand exchange (SALE) method. Initially, 0.10 g (0.24 mmol) of the synthesized NICS-24 were dispersed in a mixture of 3.5 ml of methanol (100% Fluka) and 3.5 ml of demineralized water by stirring at room temperature for 5 minutes. Subsequently, the appropriate amount of the selected linker was added to the mixture. After 15 minutes of stirring, the reaction mixture was then transferred in a 23 ml Parr stainless steel Teflon-lined autoclave and then heated at 120 ˚C for 72 hours. The obtained white powder was washed with ethanol and then dried at ambient conditions. The preparation of the materials is reproducible showing comparable CO_2 isotherms (**Figure S25)**.

Ligand-assisted treatment with 3-(trifluoromethyl)-1*H*-1,2,4-triazole (**FMeTz**): Firstly, 0.10 g (0.24 mmol) of NICS-24 was dispersed in a mixture of 3.5 ml of methanol (100% Fluka) and 3.5 ml of demineralized water by stirring at room temperature for 5 minutes. Then, 3-(trifluoromethyl)-1*H*-1,2,4-triazole (FMeTz) (95% BLDpharm) (at different molar ratios, **Table S1**) was added to the mixture. After 15 minutes of stirring, the reaction mixture was transferred to a 23 ml Parr stainless steel Teflon-lined autoclave and then heated at 120 ˚C for 72 hours. XRD patterns of the products with different loadings of FMeTz are provided in **Figure S1**. Samples are denoted as *n*FMeTz, where *n* represents molar % of added FMeTz, relative to the guanazole amount used for the synthesis of pristine NICS-24.

Ligand-assisted treatment with 3-amino-5-methylthio-1*H*-1,2,4-triazole (**SMeTz**): Firstly, 0.10 g (0.24 mmol) of NICS-24 was dispersed in a mixture of 3.5 ml of methanol (100% Fluka) and 3.5 ml of demineralized water by stirring at room temperature for 5 minutes. Then, 3-amino-5-methylthio-1*H*-1,2,4-triazole (SMeTz) (97% Aaron Chem) (at different molar ratios, **Table S1**) was added to the mixture. After 15 minutes of stirring, the reaction mixture was transferred to a 23 ml Parr stainless steel Teflon-lined autoclave and then heated at 120 °C for 72 hours. XRD patterns of the products with different loadings of SMeTz are provided in **Figure S2**. Samples are denoted as *nS*MeTz, where *n* represents molar % of added SMeTz, relative to the guanazole amount used for the synthesis of pristine NICS-24.

Methods

Powder XRD data were collected on a PANalytical X'Pert PRO diffractometer using CuKα radiation (l = 1.5418 Å) at room temperature in an angular range of 5 – 50° 2θ with a step size of 0.034° and 100 s per step using fully-opened 100 channel X'Celerator detector. Unit cell parameters for pristine and SALE-modified NICS-24 samples were obtained by Le Bail profile refinement of powder X-ray diffraction data. Prior to refinement, background contribution was modeled using a 6th order Chebyshev polynomial. Peak shapes were described using a Pearson VII function, which allows the refinement of both peak width and shape parameters taking into account microstrain and crystallinity variations. Le Bail refinements were carried out using Topas Academic v6, with the parent NICS-24 unit cell used as the initial metric. During refinement, only background, zero shift, scale factor, peak-shape parameters, and lattice constants were varied.

Morphology, phase purity, and size of the crystals were examined using a Thermo Fisher Scientific Apreo 2S Thermo field-emission gun (FEG) scanning electron microscope equipped with an AZtec Live Ultim Max SDD detector for energy-dispersive X-ray spectroscopy (Oxford Instruments). EDS mapping and point analyses were employed to evaluate elemental spatial distributions and to perform semi-quantitative compositional analyses. For determination of Zn-to-ligand molar ratios, Zn and N elemental quantifications were used, as these elements provide the most reliable basis for stoichiometric comparison. Quantification of oxygen and carbon was not considered reliable due to the potential presence of residual or adsorbed water within the materials and the elevated carbon background arising from sample mounting on carbon tape. XPS analysis was performed on a PHI VersaProbe III spectrometer equipped with a hemispherical analyzer and a monochromatic Al $K\alpha$ X-ray source. Quantitative Zn bulk analysis was performed on a Varian 715-ES ICP Optical Emission Spectrometer.

Thermogravimetric measurements were performed on TA Instruments Q5000 apparatus in air flow of 10 ml/min at the heating rate of 5 ˚C/min. Sorption data for CO_2 and N_2 were collected on IQ3 Anton Paar adsorber. Prior to the measurements, the samples were outgassed at 150 ˚C for 4h at a heating rate of 2 ˚C/min. Specific surface area was determined by BET theory based on CO_2 isothermal data measured at 273 K (**Table S4** and **Figure S18**).

Pore size distributions (PSD) were estimated from CO_2 adsorption isotherms using a non-local density functional theory (NLDFT) approach as implemented in ASiQwin software package (**Figure S19**). The fitting was performed using a carbon slit-pore kernel, which is commonly applied for the analysis of ultramicroporous materials when CO_2 is used as the probe molecule at 273 K. The fitting was performed over the whole pressure region.

Positron annihilation lifetime spectroscopy (PALS) was used to evaluate the free pore volume size and the relative concentration of free volume sites in the samples. Prior to measurement, the samples were activated under vacuum at 150 °C for 4 hours. All measurements were carried out under vacuum at 20 °C. The powdered samples were packed 1.5 mm thick on either side of the 3.5 MBq ^{22}NaCl positron source sealed in Mylar. Data was acquired on an EG&G Ortec fast–fast coincidence system. For each sample, at least five spectra were collected, each containing 1×10^6 total counts, and a timing resolution of 230 ns. The resulting spectra were analyzed with LT software (version 9.0),[3] applying a source correction of 1.415 ns and 3.6%. Three lifetime components were resolved for each sample, where the third lifetime (tau3) arises from ortho-positronium (o-Ps) annihilation and provides information about the size and number of pores. The average free volume diameter is calculated from Tau3 using the Tao–Eldrup model.[4,5]

CO_2 kinetic analysis, cycling and water isotherms were performed using a gravimetric sorption analyzer (DVS Carbon, Surface Measurements Systems) at 25 °C in the range of 10–95% using a flow of 200 ccm with N_2 carrier gas. Prior to the measurements, samples were degassed using identical protocols – 150 °C for 4h at a heating rate of 2 °C/min. For kinetic studies initial sample masses were 22 mg, 25 mg, and 16 mg for NICS-24, 40FMeTz, and 60SMeTz respectively.

Isosteric heat of adsorption were determinated using the Clausius-Clapeyron equation based on adsorption isotherms collected at 273 K and 283 K. The experimental isotherms were fitted with the Dual-Site Langmuir (DSL) model:

$$q_e = \frac{q_{m1}K_1P}{1+K_1P} + \frac{q_{m2}K_2P}{1+K_2P}$$

where q_e represents the equilibrium adsorbed capacity at a given partial pressure P, q_{m1} and q_{m2} correspond to the maximum adsorption capacities of sites 1 and 2 respectively, K_1 and K_2 are Langmuir constants for sites 1 and 2 respectively.

The kinetic profiles were fitted using multiple models, including the pseudo first order (PFO), pseudo second order (PSO), and Avrami equations (**Figure S28** and **Table S6**). The respective rate expressions are given as:

PFO: $q_t = q_e(1 - e^{-k_1 t})$

PSO: $q_t = \frac{q_e^2 k^2 t}{1+q_e k_2 t}$

Avrami model: $q_t = q_e(1 - e^{-k_A t^n})$

Were q_t and q_e are the adsorbed amounts at time t and at equilibrium, respectively, k_1, k_2, and k_A are rate constants, and n is the Avrami exponent. The quality of each fit was assessed using the coefficient of determination (R^2).

The linear driving force (LDF) model is expressed as:

$$\frac{dq_t}{dt} = k_{LDF}(q_e - q_t)$$

And its integrated form: $q_t = q_e(1 - e^{-k_{LDF} t})$

The LDF rate constant was subsequently used to estimate approximately effective diffusivities[6] (Figure S27) according to:

$$Deff = \frac{k_{LDF} R_p^2}{\alpha}$$

Were Rp is the partical radius and α is the shape factor. While α=15 is typically used for spherical diffusion, a value of α=8, corresponding to cylindrical geometry[7], was adopted here to better approximate the morphology of the crystallites observed by SEM imaging (**Figure S10**).

Temperature swing regeneration experiments were performed in DVS Vacuum gravimetric analyzer (Surface Measurements System) in static mode dosing CO_2 to 1 bar from vacuum. Prior the measurements, samples were degassed using identical protocols – 150 °C for 4h at a heating rate of 2 °C/min. Regeneration temperatures varied from 60 °C to 120 °C.

The CO_2 breakthrough experiments were performed using a quartz tubular reactor (10 mm I.D.), positioned inside the Carbolite VST 12/300 vertical furnace. The powdered sample (75 mg) was positioned between two flocks of quartz wool and activated in helium flow (50 ml/min, Messer, purity 5.0) at 150 °C for 4 h (ramp = 2°C/min). Sample temperature was measured using a K-type thermocouple positioned inside the sample layer. Operated 4-way valve (by DK-Lok) was actuated to make an instantaneous step change from He to 1% CO_2 99% Ar flow (50 ml/min). Gases leaving the reactor were continuously analyzed by MS (Hiden analytical, model DSMS): Ar: m/z= 40 and CO_2: m/z= 44. After 40 minutes of sample saturation, a switch back to helium was made. After 40 min degassing at 28 °C in helium flow, sample temperature was increased with a ramp of 9 °C/min to 150 °C and maintained at 150 °C for 30 minutes to complete CO_2 desorption.

FTIR spectra were collected from ATR FT-IR spectrometer Spectrum Two (PerkinElmer) with $LiTaO_3$ detector equipped. The measurements were conducted in the range between 4000 and 500 cm^{-1}.

^1H solution-state nuclear magnetic resonance experiments were performed on a 600 MHz Bruker Avance NEO spectrometer equipped with a 5 mm BBFO SmartProbe. All samples were dissolved in DMSO-d_6 containing 10% DCl. Frequency axes were referenced to tetramethylsilane (TMS). All spectra were processed using the TopSpin software package and externally referenced to residual DMSO resonance (δ = 2.50 ppm). A single-pulse experiment with a 30° excitation pulse of 6 μs was used with 16 scans per spectrum and a recycle delay of 1 s.

Solid-state nuclear magnetic resonance (SSNMR) experiments were performed on a 600 MHz Bruker Avance NEO spectrometer equipped with a 3.2 mm H/X double-resonance MAS iProbe. All spectra were recorded at a magic angle spinning (MAS) frequency of 20 kHz. The Larmor frequencies for ^{1}H, ^{13}C, ^{15}N, and ^{19}F nuclei were 600.23 MHz, 150.93 MHz, 60.82 MHz, and 564.78 MHz, respectively. Frequency axes for ^{1}H and ^{13}C spectra were referenced to tetramethylsilane (TMS), while those for ^{15}N were referenced to nitromethane, and for ^{19}F to $CFCl_3$. All spectra were processed using the TopSpin software package and externally referenced using the ^{13}C adamantane methylene resonance (δ = 37.78 ppm).[8] For ^{1}H MAS measurements, a Hahn-echo pulse sequence (90°–τ–180°–τ–acq) was used with a 90° pulse of 2.3 μs, a 180° pulse of 4.6 μs, an echo delay τ of 50 μs, and 64 scans per spectrum, with a recycle delay of 3 s. Likewise, for ^{19}F MAS measurements, a Hahn-echo pulse sequence was used with a 90° pulse of 2.3 μs, a 180° pulse of 4.6 μs, an echo delay τ of 50 μs, and 128 scans per spectrum, with a recycle delay of 10 s. For the ^{1}H–^{13}C and ^{1}H–^{15}N cross-polarization (CP)-MAS NMR experiments, ^{1}H magnetization was prepared using a 2.3 μs 90° pulse, followed by tangent-ramped CP blocks of 6.8 ms and 6.5 ms, respectively. High-power ^{1}H decoupling was applied during acquisition. The numbers of scans were 256–2048 and 5120–51200, respectively, with recycle delays of 2.5 s and 1.3 s, respectively. The 2D ^{1}H–^{13}C CP-HETCOR spectrum was acquired with 300 increments along the indirectly detected dimension (indirect spectral width of 10 kHz), 64 scans per increment, and a 2.5 s recycle delay. 2D NMR spectrum was plotted using the ssNake software.[9]

Sample-packed zirconia NMR rotors were put in a laboratory-built gas-dosing manifold, adapted from literature reports on analogous materials (**Figure S43**).[10–13] The sample-containing rotors were first degassed and subsequently heated under vacuum. The heating program consisted of a temperature ramp of 1 ˚C/min followed by 9 h at 80 ˚C, and then a ramp of 0.5 ˚C/min followed by 4 h at 120 ˚C. Afterwards, the samples were cooled to room temperature under vacuum and then dosed with 1 atm of $^{13}CO_2$ (Sigma-Aldrich, 99 atom % ^{13}C; <3 atom % ^{18}O) for 1 h. The rotors were then sealed inside the manifold before subjected to ambient conditions prior to use in MAS NMR measurements.

The pulse CO_2 adsorption and desorption dynamics were analyzed by DRIFTS spectroscopy. Perkin Elmer Frontier spectrometer with an LN_2 cooled MCT detector was used to record the spectra. The wavenumber range between 2000 and 2500 cm^{-1} was analyzed with 4 cm^{-1} spectral resolution and 16 accumulations per scan. Samples were held in a vacuum drier overn night at room temperature before analysis. The powdered sample 5±0.1 mg was positioned into the ceramic cup inside the reaction chamber (Pike Scientific) and degassed for 60 min at 150 ˚C in vacuum (Pfeiffer vacuum HiCube membrane and turbomolecular pump). After degassing, the samples were cooled to room temperature (25 ± 1 ˚C) and brought to ambient pressure in helium (Messer, purity 5.0). A flow of 15 ml/min of He was established and background spectrum was recorded. A manually operated 4-port valve by DK-Lok was used to switch from the He flow to 1000 ppm CO_2/N_2 flow (10 ml/min) and saturate the samples with CO_2.

Dynamic breakthrough experiments were performed under ambient-relevant conditions using a Catalyst Analyzer Belcat II (Microtrac, Montgomeryville and York, Pennsylvania, USA). Approximately 50 mg of sorbent was packed into a fixed-bed column (18 cm length, 1 cm

internal diameter), resulting in a bed height of about 1 cm. All experiments were conducted at atmospheric pressure. Before each adsorption cycle, the sorbents were regenerated under a nitrogen flow of 50 mL min^{-1} at 150 °C for 4 h with a heating rate of 1 °C min^{-1} to remove pre-adsorbed moisture and carbon dioxide. After cooling to 25 °C, adsorption was initiated by feeding the column with a 1000 ppm CO_2/N_2 mixture at a constant flow rate of 50 mL min^{-1} in both dry and humid environments (50% relative humidity). The adsorption phase continued until complete saturation of the sorbent was achieved. Desorption was subsequently performed under pure nitrogen (50 mL min^{-1}) at 150 °C until full regeneration of the material was confirmed. The outlet gas composition was continuously monitored using mass spectrometry (MS), generating mass chromatograms that recorded signal intensity as a function of time for selected ionic fragments. From these measurements, breakthrough times, total amounts of adsorbed CO_2 and H_2O, and the mutual influence of water and carbon dioxide on adsorption kinetics were determined. To account for system dead volume, blank experiments were conducted using a column packed with quartz wool in the absence of adsorbent. The adsorption capacities were calculated by integrating the breakthrough curves and normalizing the total adsorbed amount to the mass of the sorbent.

Table S1. Calculated reagent masses corresponding to each molar fraction used in the synthesis.

ratio (n_L/n_{N24})<br>linker	10 %	20 %	40 %	60 %	80 %	100 %
(A) m(FMeTz) [g]	0.003	0.007	0.013	0.020	0.026	0.033
(B) m(SMeTz) [g]	0.003	0.006	0.013	0.019	0.025	0.031

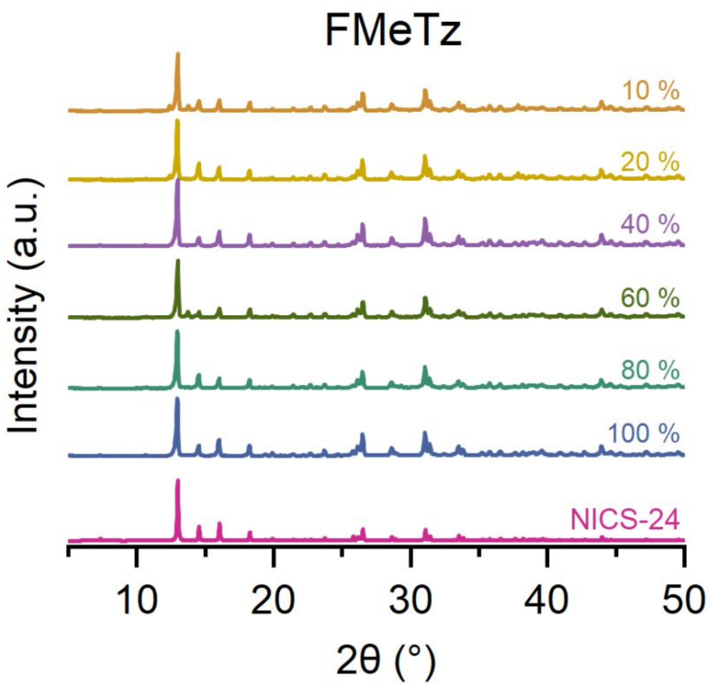

Figure S1. XRD powder patterns of modified NICS-24 products with the indicated FMeTz loadings.

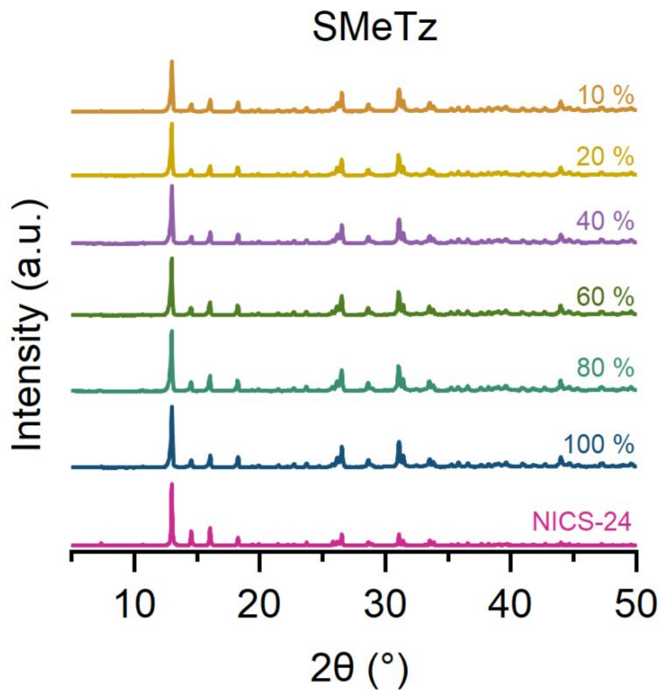

Figure S2. XRD powder patterns of modified NICS-24 products with the indicated SMeTz loadings.

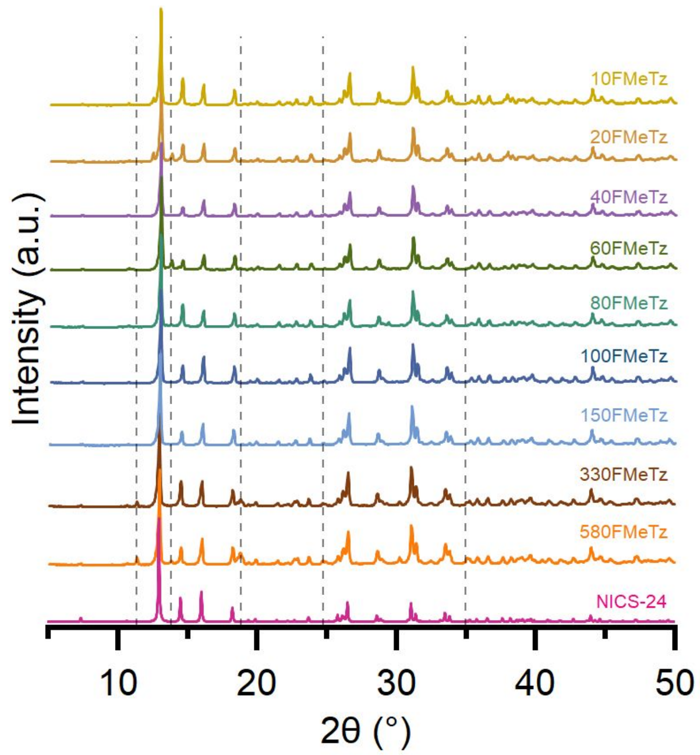

Figure S3. XRD powder patterns of samples with varying FMeTz loadings, showing the emergence of a secondary phase at ≥60% added linker.

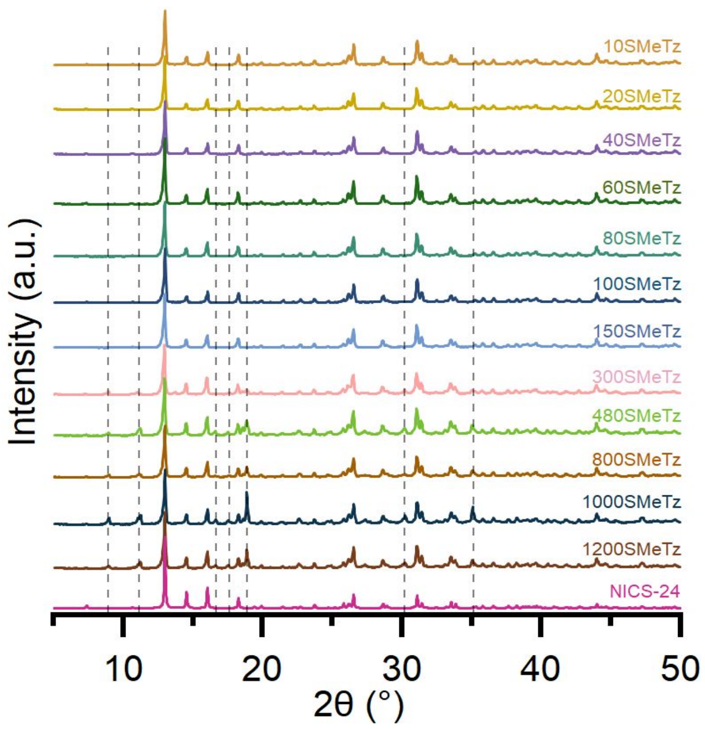

Figure S4. PXRD patterns of samples varying the SMeTz loadings, showing that a secondary phase emerges only at 30-fold excess or higher of SMeTz relative to the guanazolate linker in the parent framework.

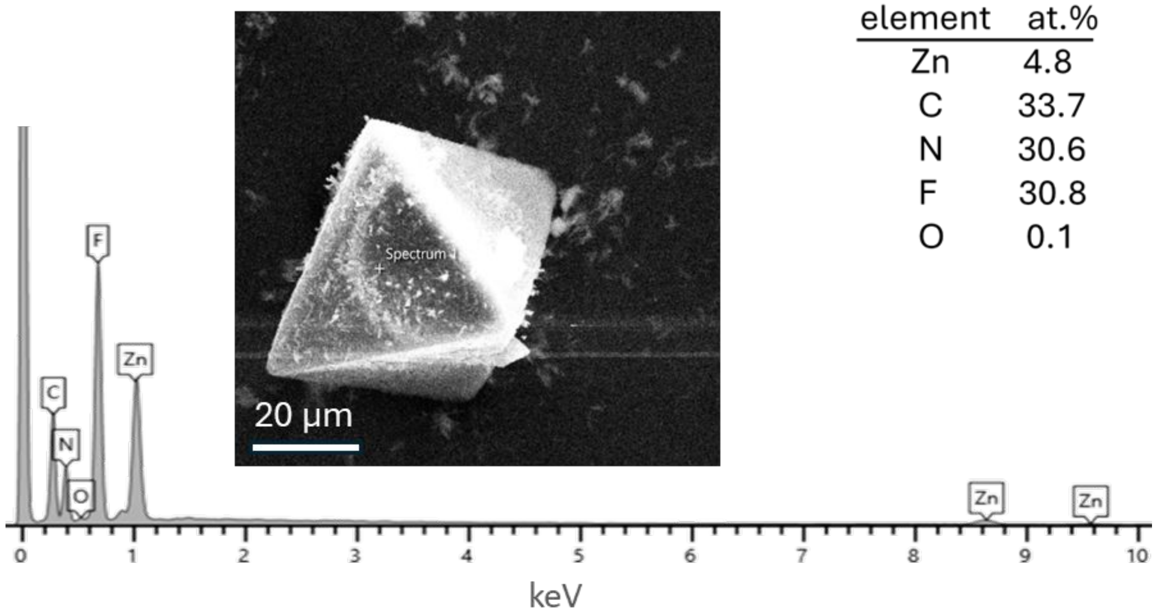

Figure S5. SEM image and corresponding EDS spectrum of an individual crystalline impurity observed in the 40FMeTz sample. Element quantification indicates a F-containing Zn–organic phase, that can be assigned to Zn–FMeTz complex.

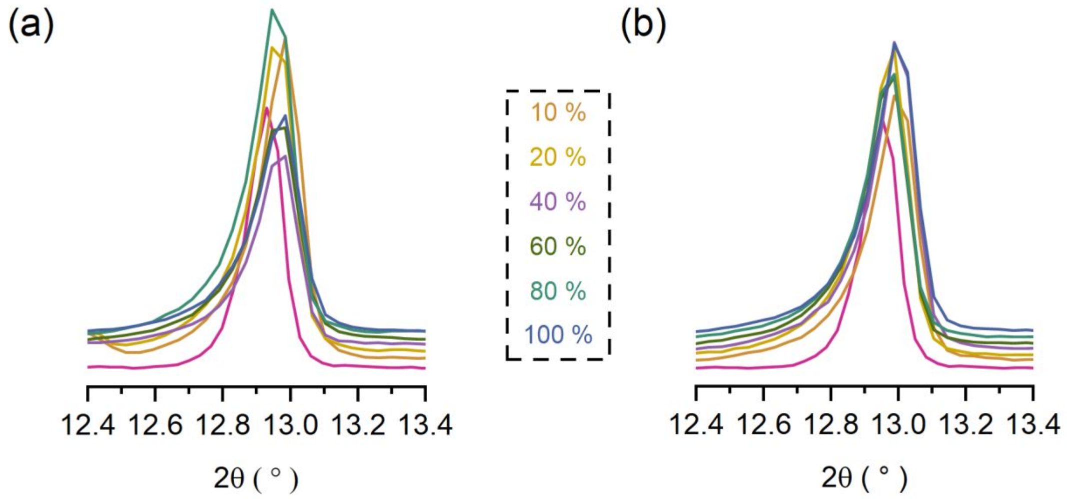

Figure S6. XRD powder patterns focused on the most intensive peak correponding to (-1 2 0) crystal plane with indicated loadings of (a) FMeTz and (b) SMeTz.

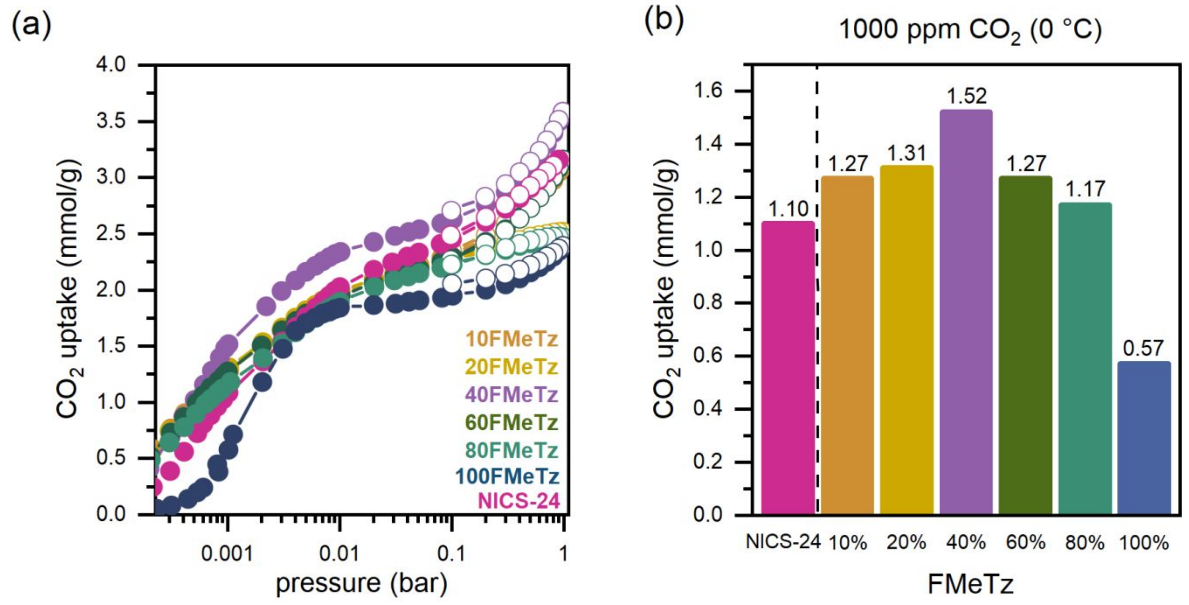

Figure S7. (a) CO_2 isotherms of FMeTz-modified materials with the indicated modulator loadings compared to the pristine NICS-24 measured at 0 ˚C. Adsorption points – full symbols, desorption points – empty symbols; (b) equilibrium CO_2 uptake at 1000 ppm of partial pressure.

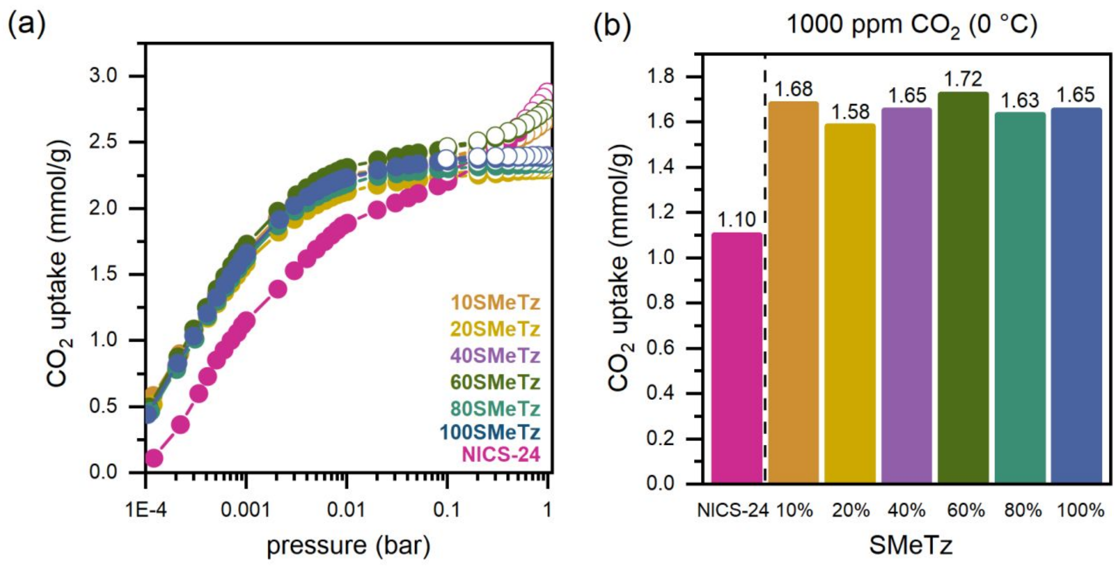

Figure S8. (a) CO_2 isotherms of SMeTz-modified materials with the indicated modulator loadings compared to the pristine NICS-24 measured at 0 ˚C. Adsorption points – full symbols, desorption points – empty symbols; (b) equilibrium CO_2 uptake at 1000 ppm of partial pressure.

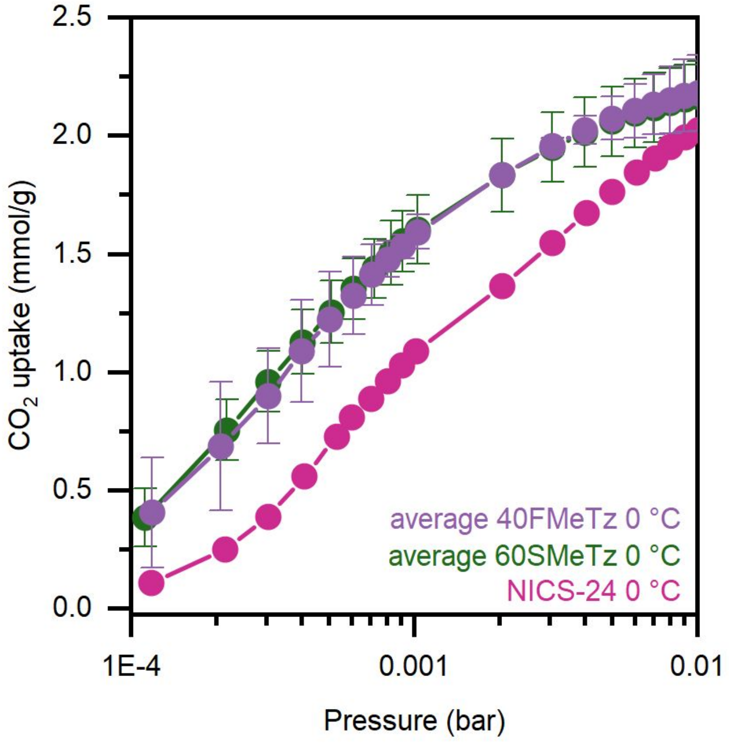

Figure S9. CO_2 adsorption isotherms measured at 0 ˚C for pristine NICS-24 and SALE-modified (40FMeTz and 60SMeTz), comparing individual batch measurements with averaged isotherms obtained from 20 independent synthesis bathces. Symbols represent mean CO_2 uptake values, while error bars indicate the standard deviation across batches.

Table S2. Unit cell volume values determined by Le Bail analysis for the samples with different FMeTz and SMeTz loadings.

loading	0%	10%	20%	40%	60%	80%	100%
**FMeTz (Å)**	1455.6(1)	1450.5(3)	1450.0(3)	1448.7(2)	1450.9(3)	1450.0(2)	1449.1(2)
**SMeTz (Å)**	1455.6(1)	1450.0(2)	1448.2(2)	1448.8(2)	1448.3(2)	1448.3(2)	1448.7(2)

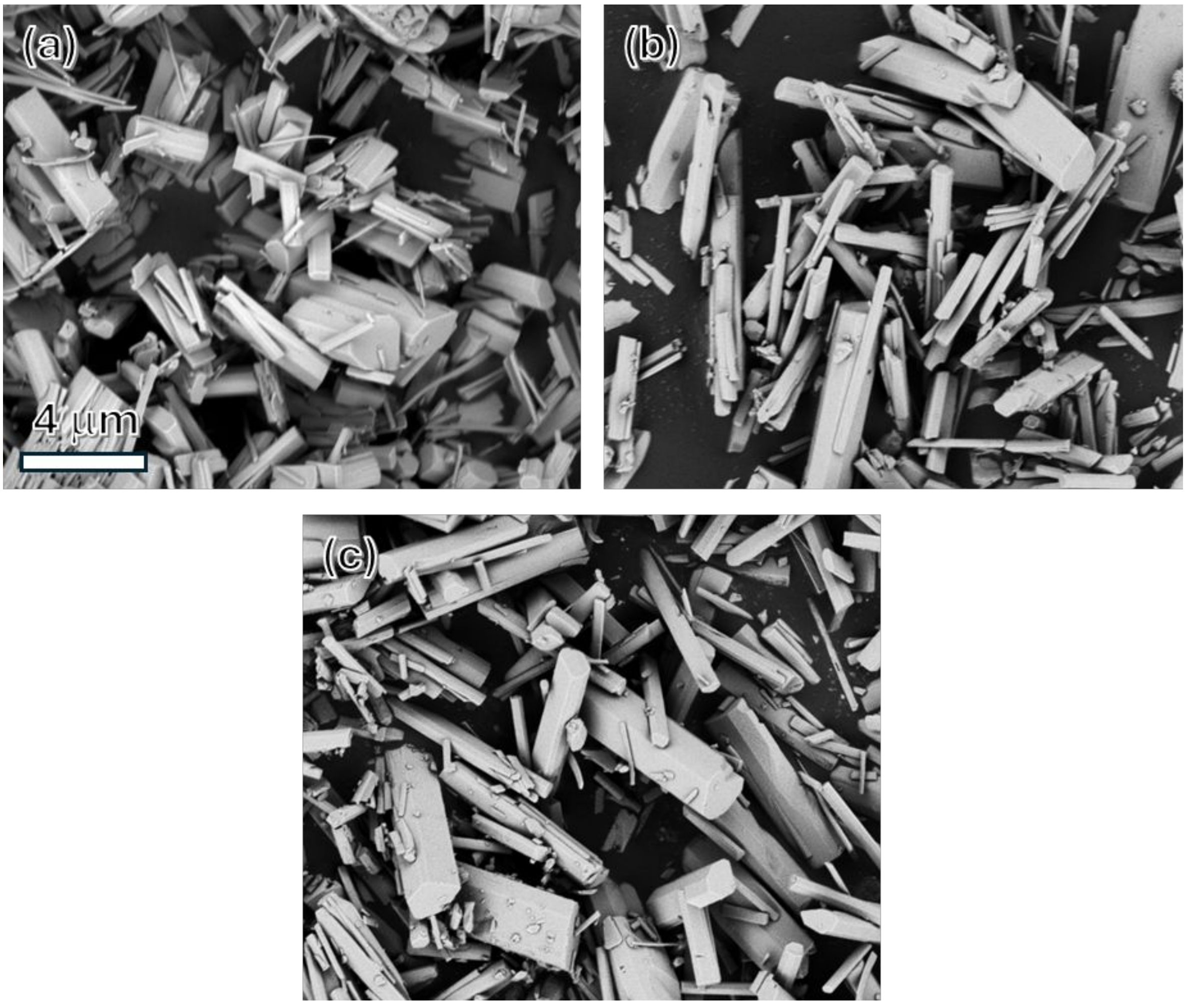

Figure S10. SEM micrographs of (a) pristine NICS-24, (b) 40FMeTz and (c) 60SMeTz. All samples display the characteristic elongated prismatic crystal morphology typically ranging from 2–6 μm in length and 0.3–1 μm in width, with well-defined facets and smooth external surfaces. The particle-size distribution and overall crystal shape remain essentially unchanged after ligand-assisted modification, indicating that incorporation of FMeTz or SMeTz does not affect crystal growth habits or induce fragmentation.

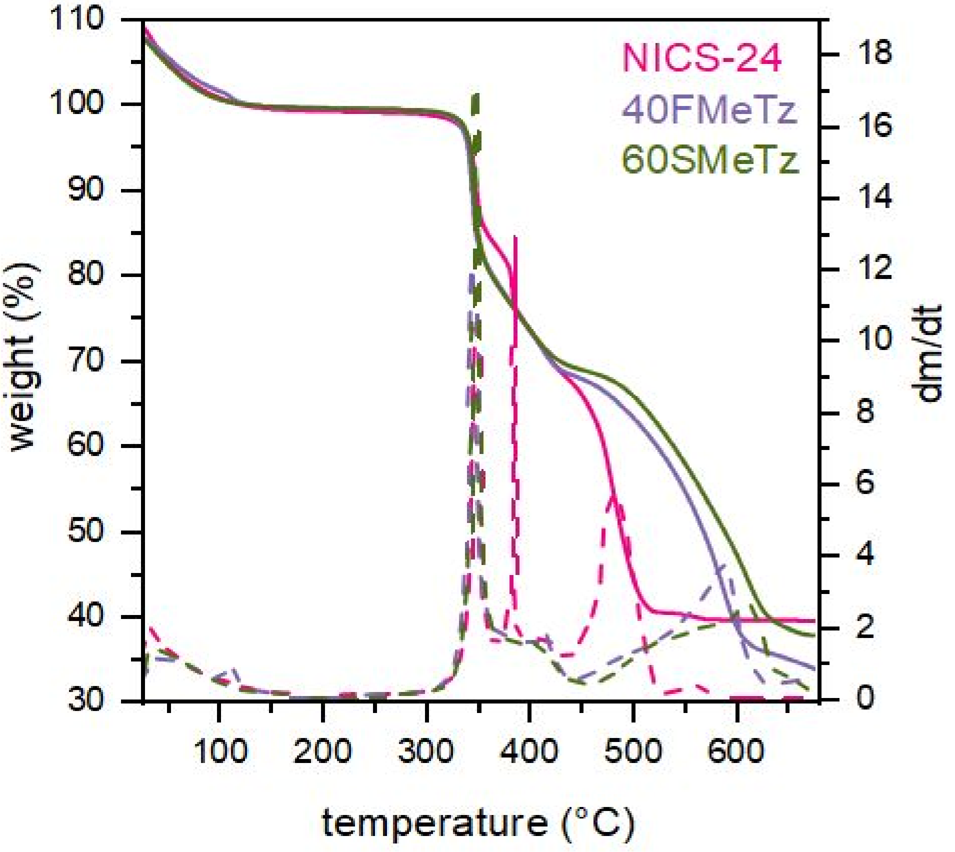

Figure S11. TGA (solid lines) and DTG (dashed lines) curves of pristine NICS-24, 40FMeTz and 60SMeTz, normalized to dry mass selected at 200 ˚C. Normalization removes contributions from residual solvent and highlights differences in intrinsic organic/inorganic content. The dominant mass-loss event corresponds to framework decomposition, followed by the formation of ZnO as the final residue.

After normalization to the dry mass at 200 ˚C, pristine NICS-24 exhibits a final ZnO residue of 39.2, in perfect agreement with theoretical value. This confirms full structural integrity and complete decomposition to ZnO. In contrast, the modified samples show lower final residues, approximately 35% for 40FMeTz (~4% reduction) and 37.7% for 60SMeTz (~1.5% reduction). A reduced inorganic residue indicates a proportionally higher organic content, consistent with two possible contributions: (1) Partial incorporation of heavier triazolate linkers, increasing the organic fraction per unit mass; (2) Zn leaching during SALE, decreasing the inorganic Zn content. The modified samples also show distinct changes in their decomposition behavior, particularly in the second major mass-loss stage (~400 – 600 ˚C). Compared to pristine NICS-24, both 40FMeTz and 60SMeTz exhibit broader DTG peaks, a delayed onset of the second decomposition step and a slower, more gradual mass-loss profile. These features indicate that decomposition of the organic components in the modified materials proceeds through more gradual process, requiring higher temperatures for completion.

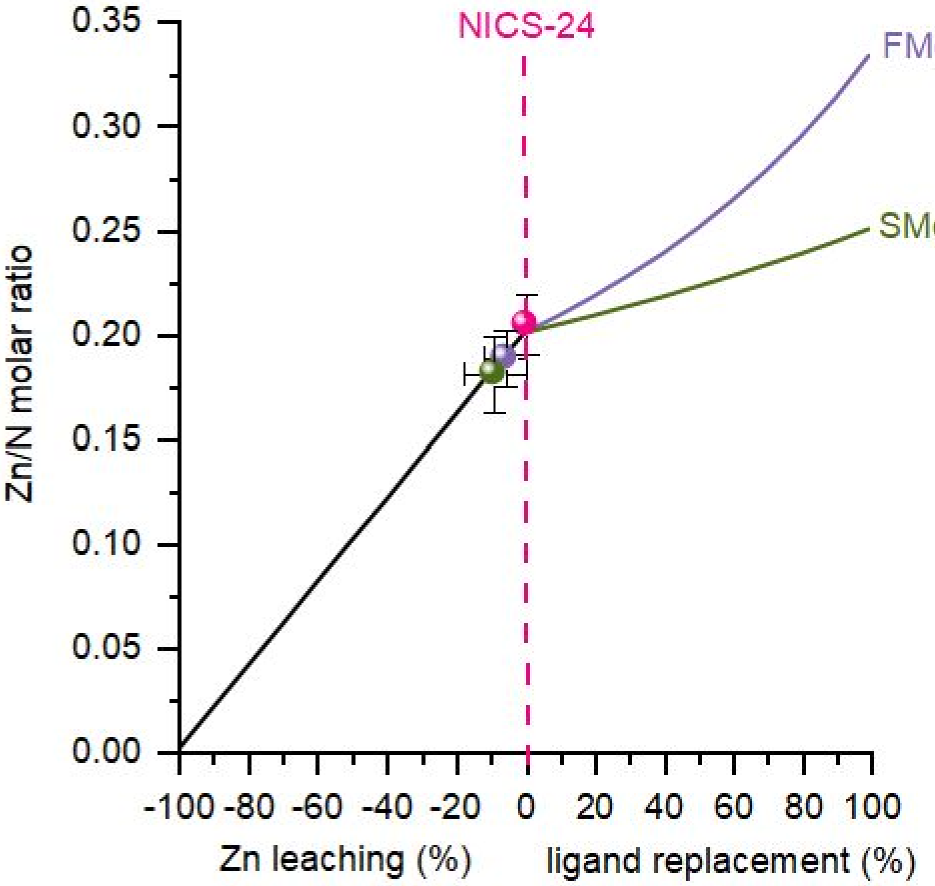

Figure S12. Zn/N molar ratios obtained from EDS analysis of 20 randomly selected crystallites for NICS-24, 40FMeTz, and 60SMeTz. Solid lines represent the theoretical Zn/N ratios expected for pure Zn deficiency (black line) or increasing degrees of corresponding ligand replacement (purple line for 40FMeTz, green line for 60SMeTz). Pink dashed intercepting the full lines determines stoichiometry Zn/N value of 0.2 for non-modified NICS-24. Values below a Zn of 0.2 indicate preferential Zn(II) loss, whereas values above 0.2 correspond to incorporation of triazolate linkers at the expense of the native organic ligand.

Table S3. Elemental composition of pristine NICS-24 and modified samples determined by EDS analysis. Values represent averages obtained from 20 randomly selected crystallites per sample, with associated standard deviations.

		Theoretical molar ratio[1]	Experimental molar ratio[2]	ligand exchange rate (%)[3]
NICS-24 ($Zn_2C_6N_{10}O_4$)	Zn/N	0.2	0.21 ± 0.01	-
60SMeTz ($Zn_2C_8N_8O_4S_2$)	Zn/N	0.25	0.18 ± 0.02	0.5 – 0.8
	Zn/S	1	157 ± 33	
40FMeTz ($Zn_2C_8N_6O_4F_6$)	Zn/N	0.33	0.19 ± 0.02	0.3 - 0.5
	Zn/F	0.33	246 ± 63	

[1]Theoretical molar ratio values considered for 100% ligand exchange; [2]Experimental molar ratios for 40FMeTZ and 60SMeTz samples; [3] Estimated ligand exchange fractions from trace S and F signals. Quantification of S and F required manual peak selection during analysis acquisition. Values therefore represent upper-bound estimates.

The EDS-derived Zn-N molar ratios reveal clear trends that distinguish between Zn leaching and true ligand exchange during the SALE treatment. Pristine NICS-24 exhibits a Zn ratio of 0.21 ± 0.01, matching the theoretical value of the ideal stoichiometry. Both modified samples display lower Zn/N ratios: 0.19 ± 0.01 for 40FMeTz and 0.18 ± 0.02 for 60SMeTz. The experimental data cluster clearly along the Zn-leaching trajectory, with no shift toward the region indicative of ligand exchange. The spread points (20 per sample) show that Zn depletion is consistent across crystallites, suggesting that leaching is a systematic defect of the ligand-assisted process rather than an isolated surface phenomenon.

The presence of fluorine and sulfur in the corresponding modified samples confirms exposure to the triazolate modulators during the process. However, their relatively low atomic percentages combined with the Zn/N ratio suggest that these elements are present only in minor amounts, likely associated with surface-bound species or impurities rather than bulk linker substitution. This is also supported by relatively high uncertainties.

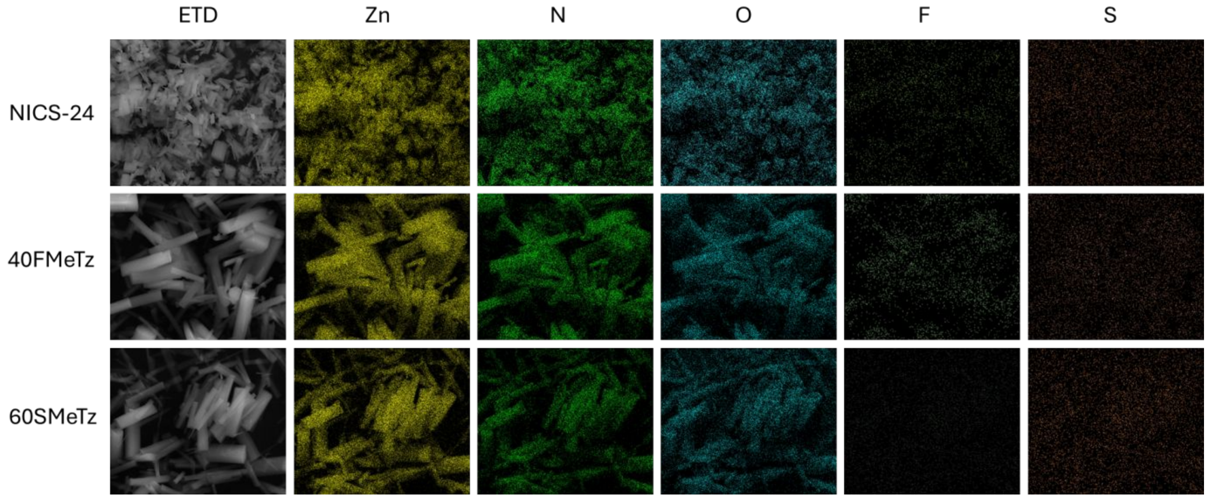

Figure S13. Elemental mapping of the areas indicated on ETD images for NICS-24, 40FMeTz and 60SMeTz samples showing spatial distribution of individual elements.

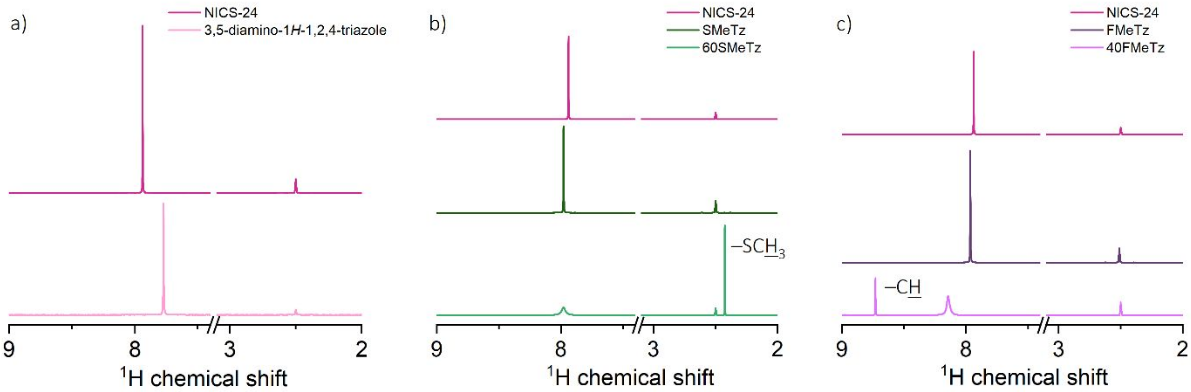

Figure S14. Comparison of ^{1}H solution-state NMR spectra of investigated MOFs and their starting materials. a) NICS-24 and 3,5-diamino-1*H*-1,2,4-triazole, b) NICS-24, SMeTz, and 60SMeTz, and c) NICS-24, FMeTz, and 40FMeTz.

Comparison of ^{1}H solution-state NMR spectra additionally confirm negligible content of both 40FMeTz and 60SMeTz linkers in the final materials. While the chemical shift of exchangeable NH_2 protons expectedly varies between the samples (8.14 to 7.77 ppm), neither SCH_3 (2.43 ppm) nor CH (8.73 ppm) resonances were observed in 60SMeTz and 40FMeTz samples, respectively.

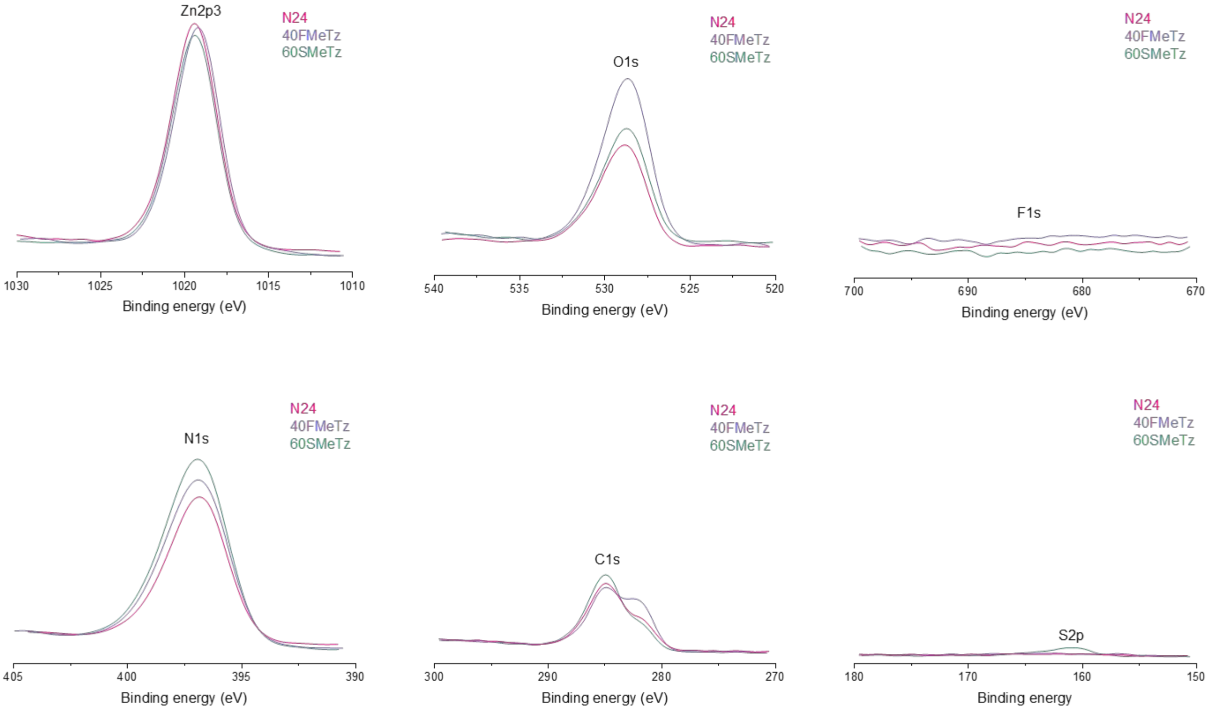

Figure S15. XPS spectra at binding energy regions relevant to Zn, O, N, C, F and S signals. Intensities are normalized to most intense (Zn2p3) peak.

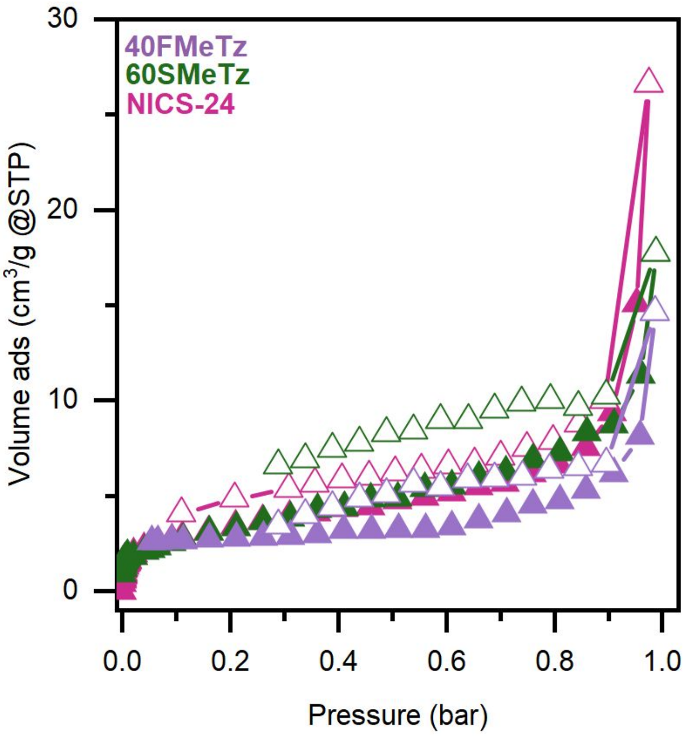

Figure S16. N_2 sorption isotherms at 77K for pristine NICS-24, 40FMeTz and 60SMeTz. Full symbols – adsorption points, empty symbols – desorption points.

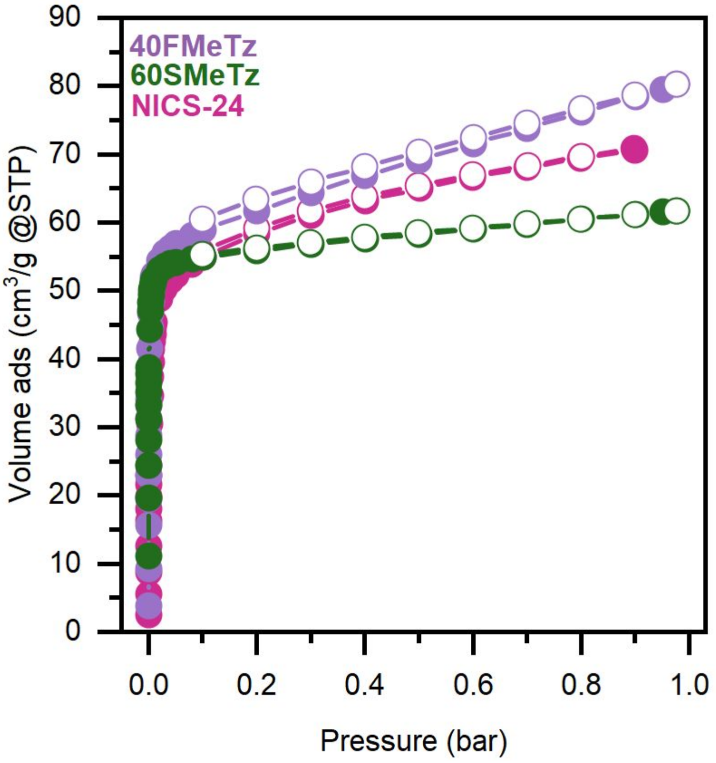

Figure S17. CO_2 sorption isotherms at 273K for pristine NICS-24, 40FMeTz and 60SMeTz. Full symbols – adsorption points, empty symbols – desorption points.

The N_2 adsorption isotherms measured at 77 K show negligible uptake for pristine NICS-24 as well as for the 40FMeTz- and 60SMeTz-modified samples (**Figure S13**). The pore system remains largely inaccessible to N_2 under cryogenic conditions despite the presence of crystallographically accessible pore volume and is further observed even after LMDE-induced defect formation. The lack of N_2 uptake reflects kinetic limitations rather than the absence of permanent porosity. Intrinsic pore apertures in NICS-24 fall within the ultramicroporous regime, approaching the kinetic diameter of N_2, which significantly hinders diffusion at 77 K. On the other hand, the strong quadropole interactions of CO_2 with polar sites within the framework enable CO_2 molecules to overcome diffusion barriers at higher temperatures, allowing effective probing of the pore network. This is indicated by CO_2 sorption isotherms

measured at 273 K that reveal well-defined Type I isotherms for all samples (**Figure S14**). The modified materials show enhanced CO_2 uptake compared to pristine NICS-24, consistent with defect-mediated increases in accessible adsorption sites and possible stronger host-guest interactions. The discrepancy between N_2 and CO_2 highlights the importance of selecting an appropriate probe molecule when characterizing ultramicroporous and defect-rich MOFs.

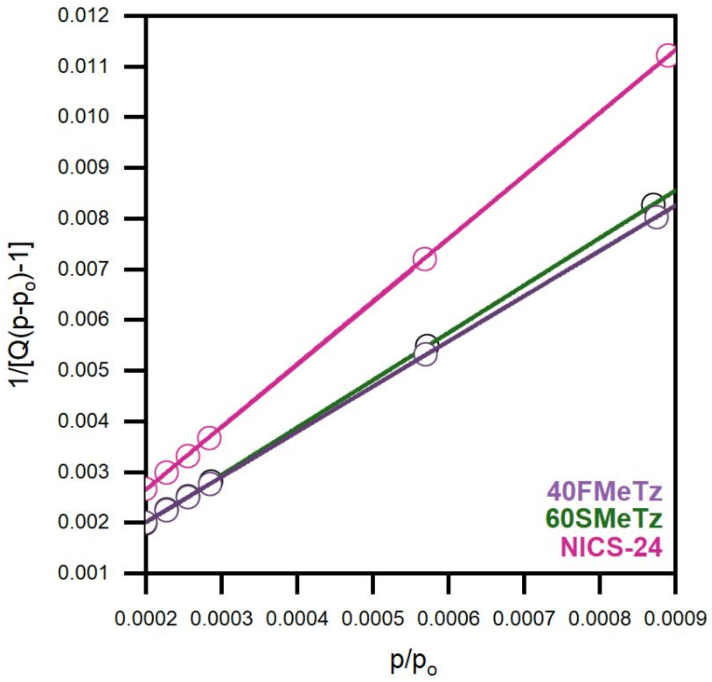

Figure S18. BET plots for NICS-24, 40FMeTz and 60SMeTz.

Table S4. BET parameters derived from CO_2 isotherms measured at 273 K for NICS-24 and all FMeTz- and SMeTz-modified batches.

sample	slope (g^{-1})	intercept (g^{-1})	correlation coeficient	C constant	S_{BET} (m^2/g)
NICS-24	12.4	0.0001772	0.999989	69946	231
10FMeTz	10.1	0.0003814	0.999987	26572	284
20FMeTz	10.2	0.0003403	0.999989	30057	281
40FMeTz	8.9	0.0002273	0.999990	39267	322
60FMeTz	10.3	0.0003709	0.999978	27867	278
80FMeTz	10.4	0.0004455	0.999993	23391	276
100FMeTz	12.0	0.0001304	0.999988	91639	240

**10SMeTz**	9.6	0.0001388	0.999999	69445	298
**20SMeTz**	10.1	0.0002465	1.0	40979	285
**40SMeTz**	9.7	0.0001362	1.0	71538	295
**60SMeTz**	9.4	0.000183	0.999999	67620	307
**80SMeTz**	9.9	0.0001406	1.0	70401	290
**100SMeTz**	9.7	0.0001439	1.0	67076	298

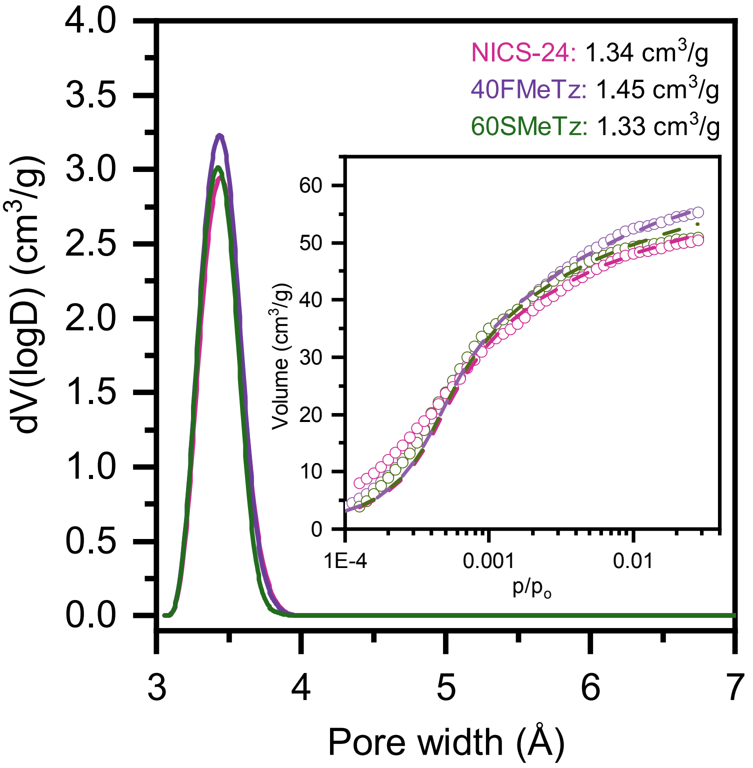

Figure S19. Pore size distributions and corresponding NLDFT fit plots derived from CO_2 isotherms measured at 273K for the investigated materials. Experimental data are shown as circles and fitted curves as dashed lines.

It should be noted that NLDFT models are based on idealized pore geometries and surface chemistries and are not parameterized for MOF systems with polar functionalities and heterogenous adsorption sites. As a result, deviations between experimental and fitted isotherms are observed, particularly in the low-pressure regime.

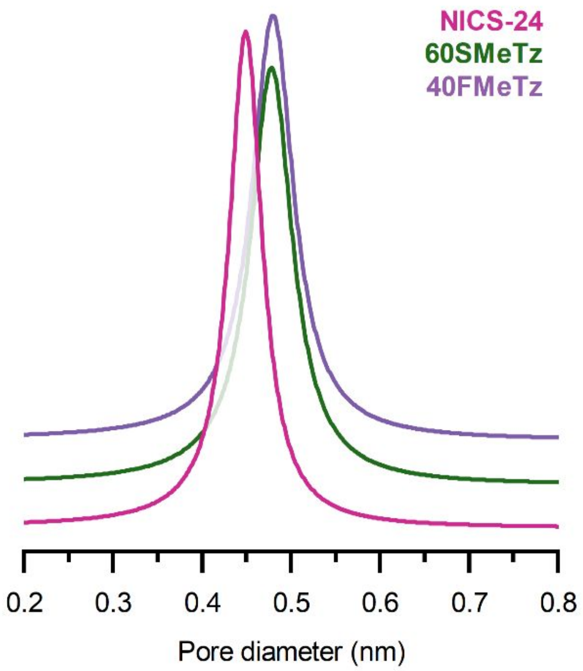

Figure S20. Pore size distribution from the PALS analysis and adapted using the PAScual v1.4 software.[14]

Table S5. PALS data for pristine NICS-24, 40FMeTz, and 60SMeTz samples.

Sample ID	Intensity *I3* (%)	±	*tau3* (ns)	±	Average pore diameter (nm)	±
**NICS-24**	30.9	0.2	1.416	0.005	**0.449**	0.001
**40FMeTz**	26.5	0.3	1.544	0.011	**0.479**	0.002
**60SMeTz**	26.0	0.2	1.533	0.006	**0.476**	0.001

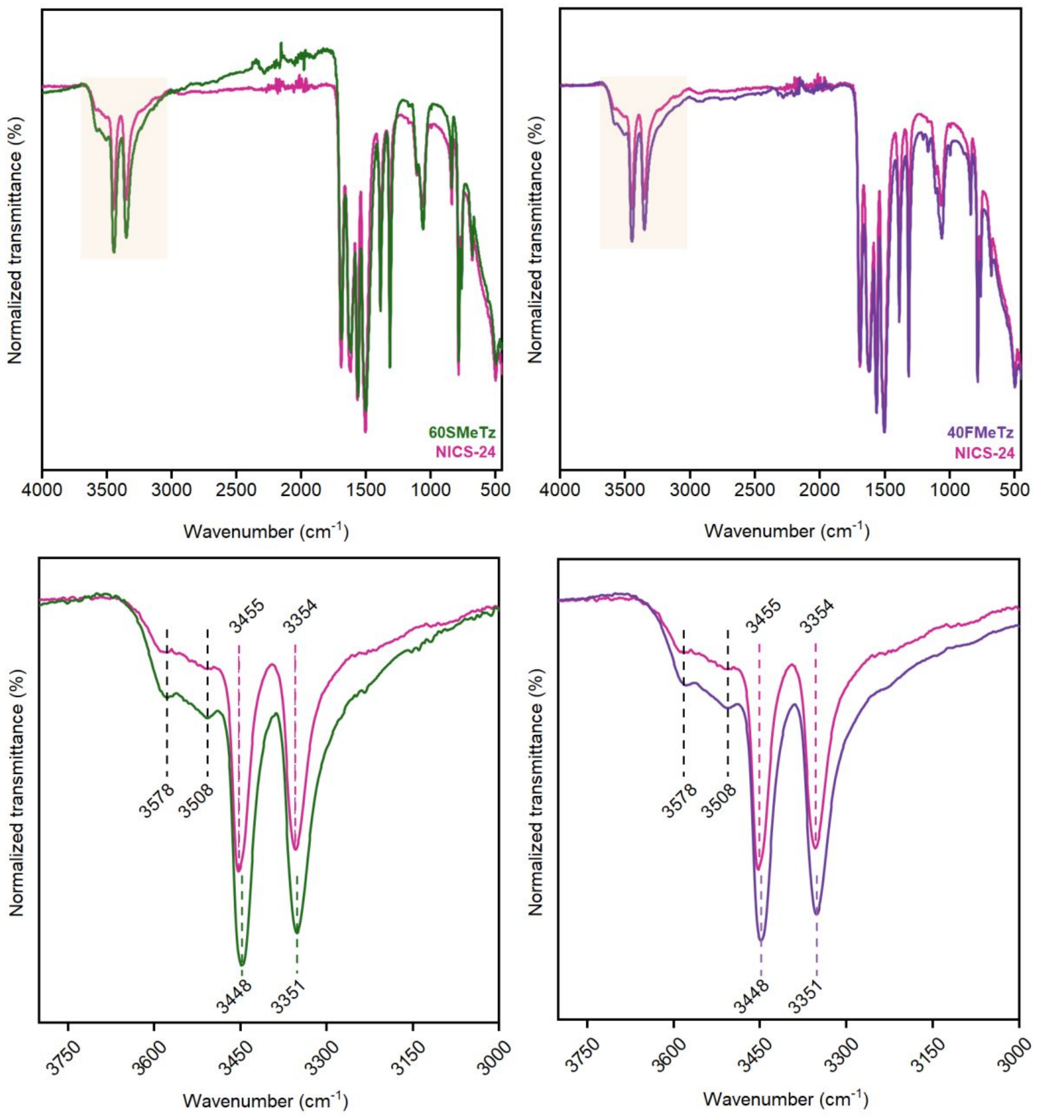

Figure S21. FTIR spectra of 60SMeTz (left) and 40FMeTz (right) modified NICS-24 samples, compared with pristine NICS-24.

FTIR spectroscopy was used to probe local chemical changes induced by LMDE in NICS-24. Pristine NICS-24 shows two broad dominant bands at 3455 and 3354 cm^{-1}, assigned to hydrogen-bonded X–H stretching modes dominated by framework N–H environments (guanazolate-derived). After the modification, both 40FMeTz and 60SMeTz main bands display minor red-shifts to 3448 and 3351 cm^{-1}, indicating that a substantial fraction of the original H-bonding/coordination environment remains intact on the bulk scale. In contrast, the modified samples show a pronounced increase in intensity of two additional bands at 3578 and 3508 cm^{-1}, which are weak in pristine NICS-24 but become clearly visible after the ligand-assisted process. The emergence of these bands is consistent with defect-associated N–H formation and aligns with the compositional evidence for Zn depletion and the structural heterogeneity observed from PXRD peak broadening.

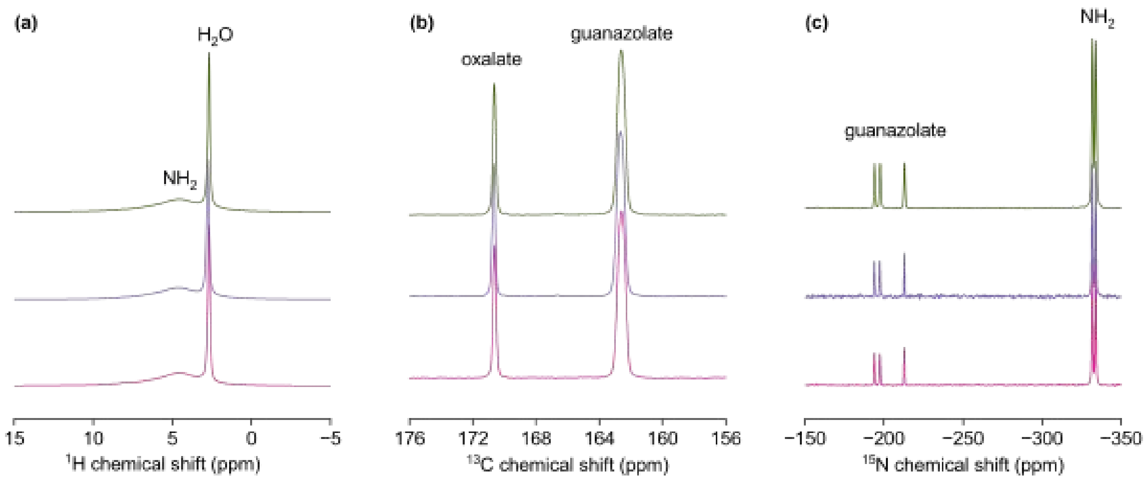

Figure S22. (a) ^{1}H MAS NMR spectra, (b) ^{1}H–^{13}C CP-MAS NMR spectra, and (c) ^{1}H–^{15}N CP-MAS NMR spectra of NICS-24 (pink), 60SMeTz (green), and 40FMeTz (purple). Spinning sidebands are denoted with asterisks.

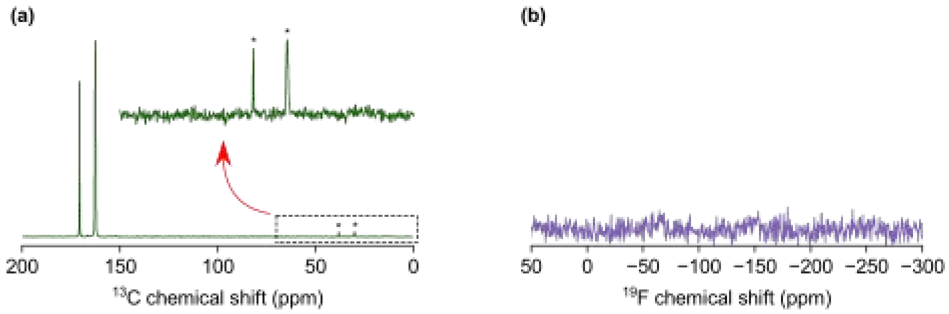

Figure S23. (a) ^{1}H–^{13}C CP-MAS NMR spectrum of 60SMeTz. No signal was observed in the aliphatic region, where the signal of -SCH_3 moiety would be expected.[15] Spinning sidebands are denoted with asterisks. (b) ^{19}F MAS NMR spectrum of 40FMeTz after probe background subtraction.

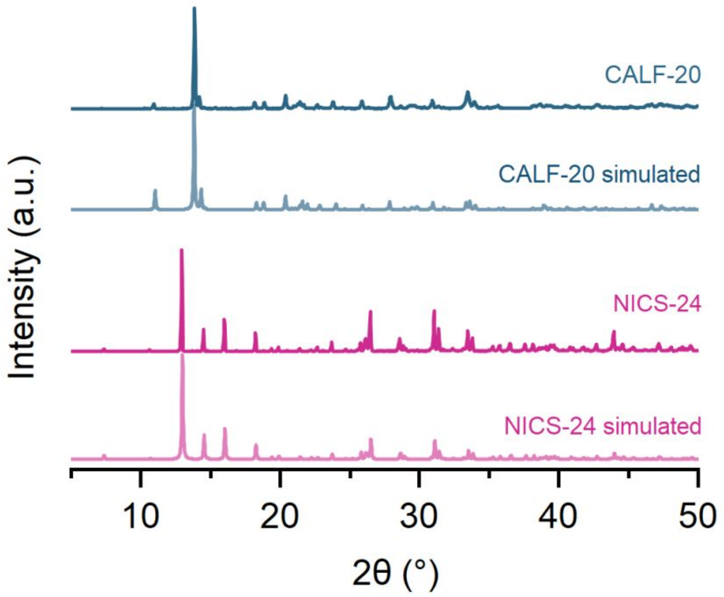

Figure S24. XRD powder pattern of CALF-20 material used as a precursor for the synthesis of NICS-24. Both products are highly crystalline and phase pure as indicated by the comparison with the calculated patterns.

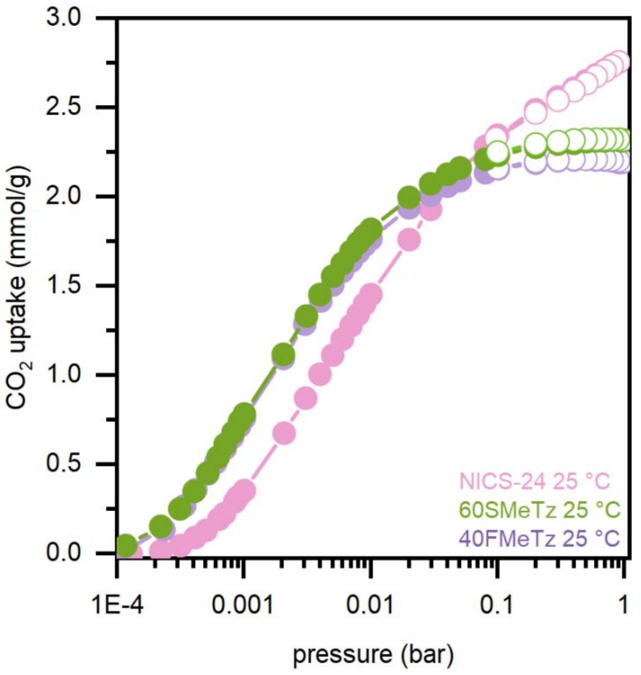

Figure S25. CO_2 isotherms of pristine NICS-24 and both modified samples (40FMeTz and 60SMeTz) measured at 25 ˚C (full circles – adsorption points, empty circles – desorption points).

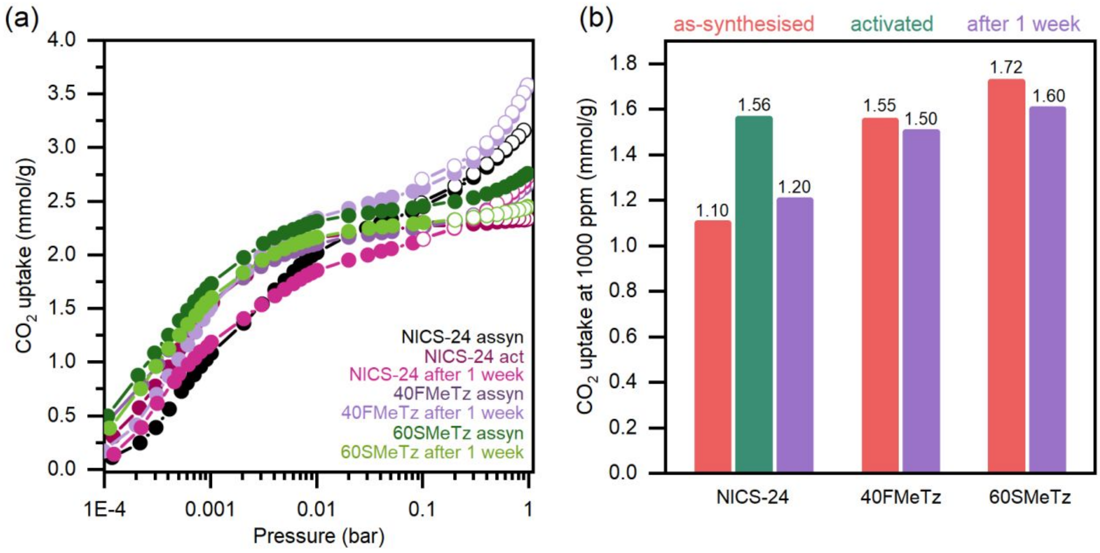

Figure S26. CO_2 sorption isotherms at 0 ˚C (0-1 bar) were measured for as-synthesized, activated, and ambient-exposed pristine NICS-24 and both modified samples.

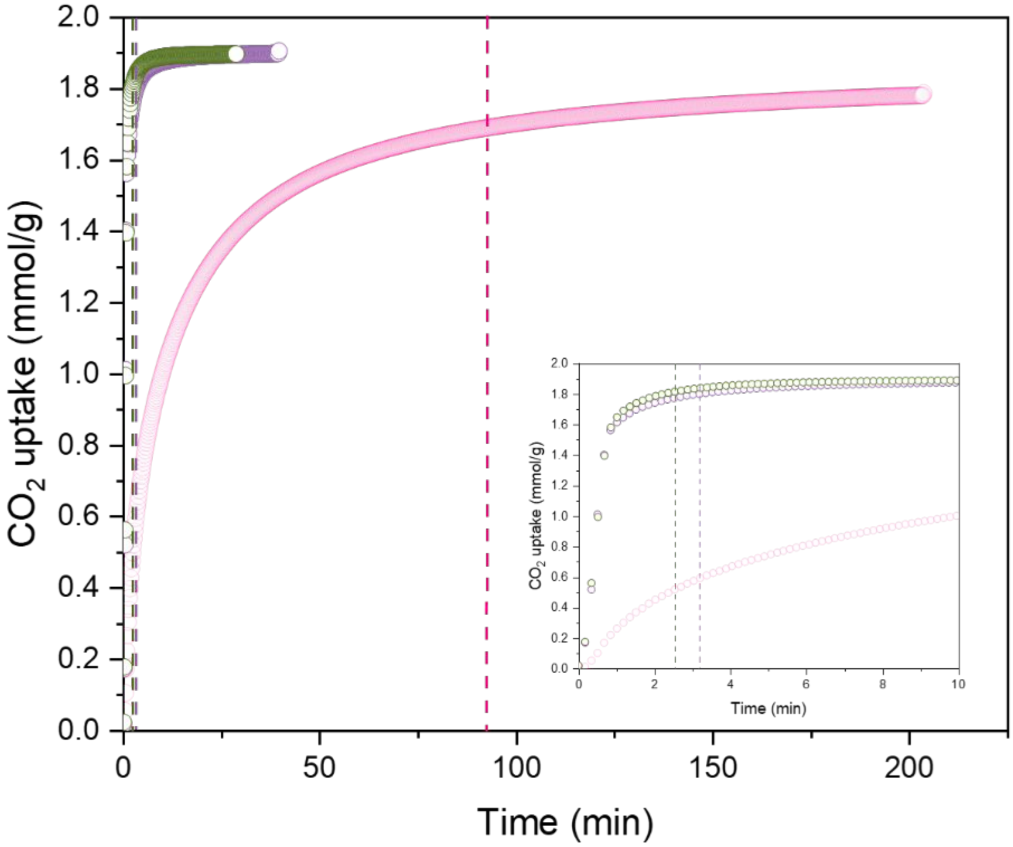

Figure S27. Kinetic profiles of CO_2 dynamic adsorption from 0 to 1 bar of relative pressure for NICS-24 (pink), 40FMeTz (violet) and 60SMeTz (green). Vertical dashed lines represent time corresponding to 95% of equilibrium uptake (t_{95}). Inset shows zoomed kinetic profiles of the time region 0–10 min to visualize the differences in t_{95} for 40FMeTz and 60SMeTz.

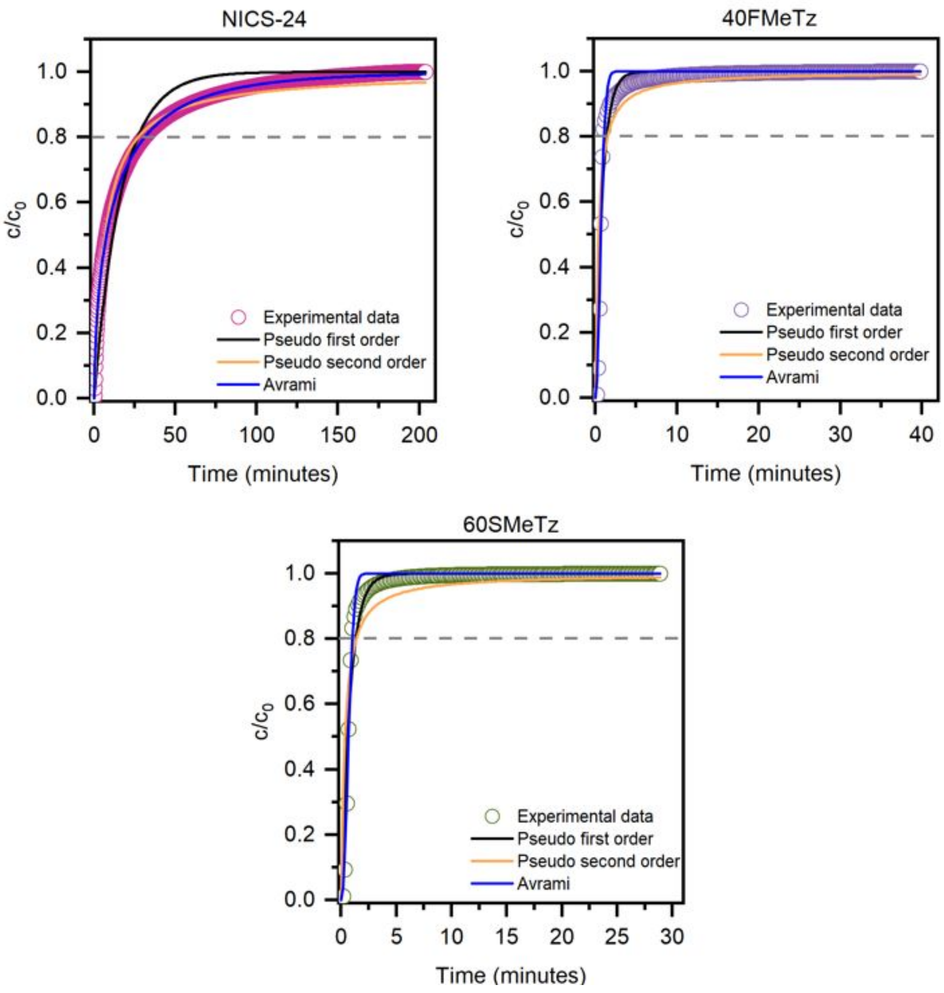

Figure S28. Kinetic profiles (Pseudo first order, Pseudo second order, and Avrami models) fitted to the experimental data points for pristine NICS-24, 40FMeTz, and 60SMeTz.

Table S6. Corresponding parameters for the fitted kinetic models of the studied samples.

	Model	R^2	k (min^{-1})	n	R^2 ($c/c_0 \leq 0.8$)	n ($c/c_0 \leq 0.8$)
NICS-24	Pseudo first order	0.8893	0.0599			
	Pseudo second order	0.9736	0.1480			
	Avrami	0.9984	0.0712	0.60	0.9967	0.62
40FMeTz	Pseudo first order	0.9455	1.1662			
	Pseudo second order	0.8627	2.6902			
	Avrami	0.9662	1.2121	1.85	0.9989	2.82
60SMeTz	Pseudo first order	0.9498	1.2264			
	Pseudo second order	0.8353	2.9198			
	Avrami	0.9841	1.2410	2.07	0.9993	2.71

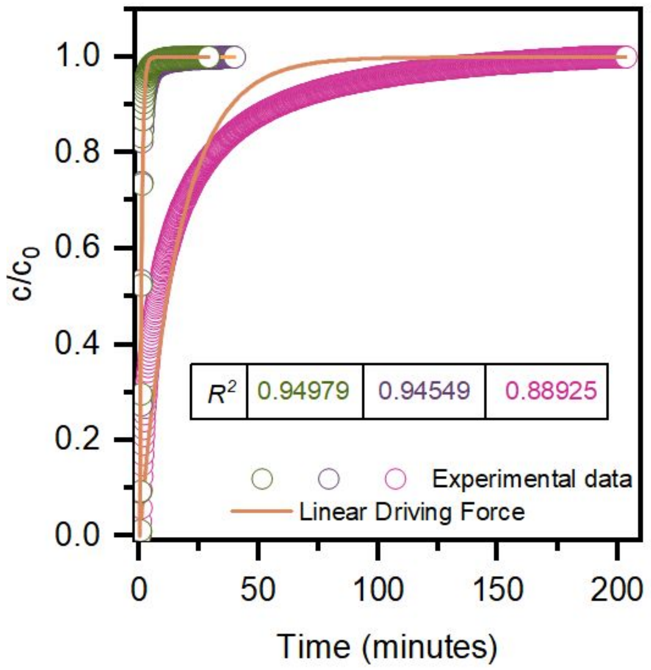

	D_{eff} (x10^{-14} m^2/s)		
**Radii (μm)**	**NICS-24**	**40FMeTz**	**60SMeTz**
1	0.01248	0.24296	0.2555
2	0.0499	0.97185	1.02198
3	0.11228	2.18667	2.29946

Figure S29. LDF model fit for all three investigated samples with effective CO_2 diffusivities (D_{eff}) of the studied adsorbents at 0.95bar and 298K, calculated using the LDF model considering crystal sizes between 1 and 3 μm which are observed from SEM imaging.

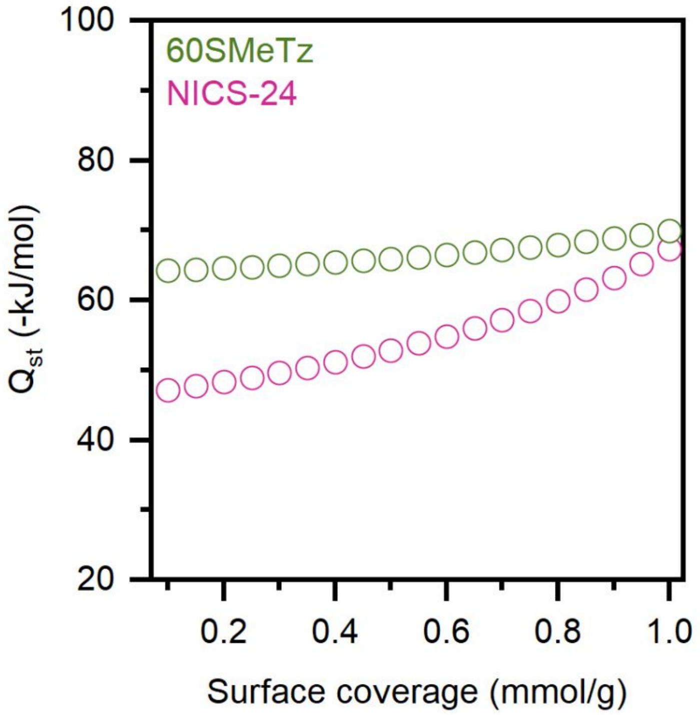

Figure S30. Isosteric heat of adsorption of CO_2 adsorption for pristine NICS-24 and 60SMeTz-modified sample, calculated via the Clausius-Clapeyron equation over a surface coverage range of 0.1-1 mmol/g.

Application of this equation was possible only for pristine NICS-24 and 60SMeTz using isothermal data from 273K and 283 K where the data at higher temperatures (293 K and 303 K) showed positive uptake trends. 40FMeTz exhibits such endothermal behavior with higher CO_2 uptakes with increased temperatures even at 273 and 283 K. Such behavior has been observed in ultramicroporous materials, where adsorption is influenced by diffusion limitation.[16] At low temperatures, restricted pore apertures and high confinement lead to slow transport of CO_2 molecules, resulting in underestimation of equilibrium uptake. Increasing temperature enhances molecular diffusivity and facilitates access to ultramicroporoes, leading to higher apparent uptake despite the intrinsically exothermic process.

Isosteric heat of adsorption is therefore not used as a descriptor of adsorption energetics. Instead, the adsorption be interpreted as arising from a trade-off between thermodynamics and transport, where defect formation primarily enhances diffusion and pore accessibility rather than significantly altering adsorption enthalpy.[17]

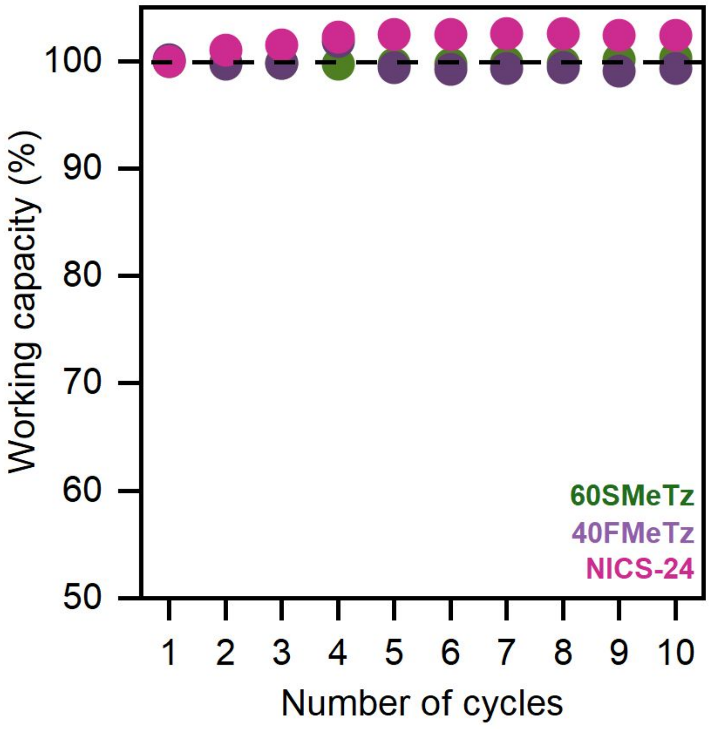

Figure S31. Loss of working adsorption capacity during 10 TSA regeneration cycles for all three samples.

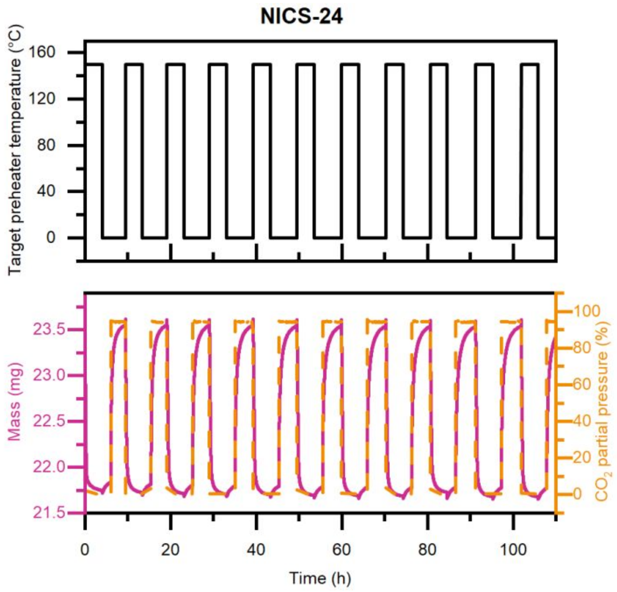

Figure S32. CO_2 adsorption cycling with temperature swing adsorption (TSA) regeneration for pristine NICS-24.

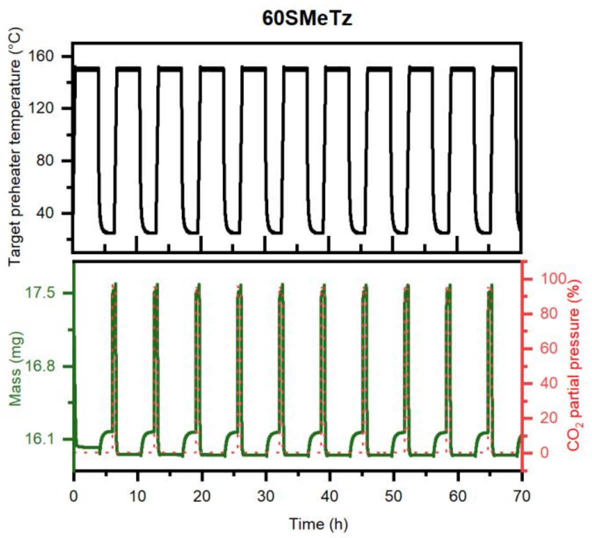

Figure S33. CO_2 adsorption cycling with temperature swing adsorption (TSA) regeneration for 60SMeTz-modified NICS-24 sample.

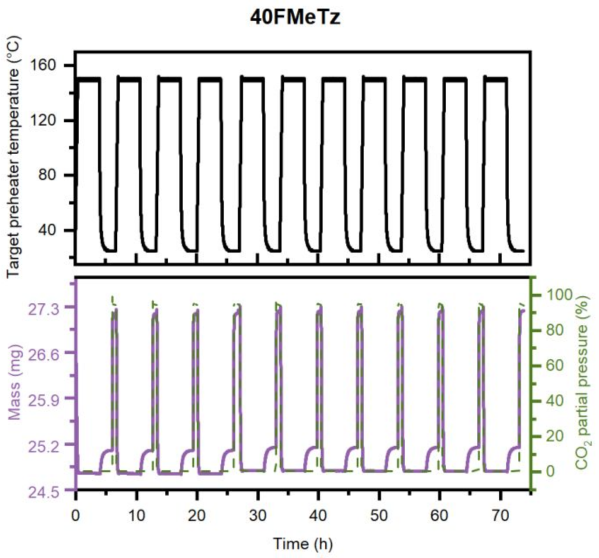

Figure S34. CO_2 adsorption cycling with temperature swing adsorption (TSA) regeneration for 40FMeTz-modified NICS-24 sample.

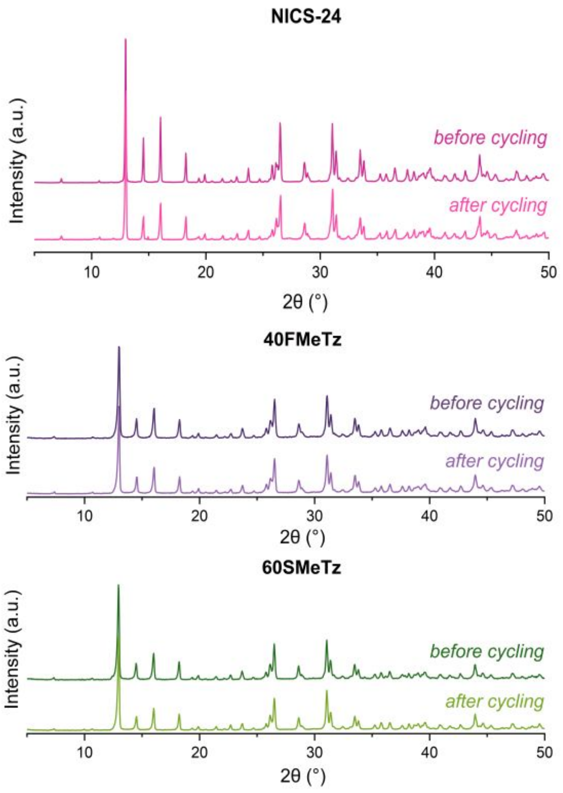

Figure S35. XRD patterns of the pristine NICS-24 and both two modified materials before and after 10 CO_2 sorption cycles.

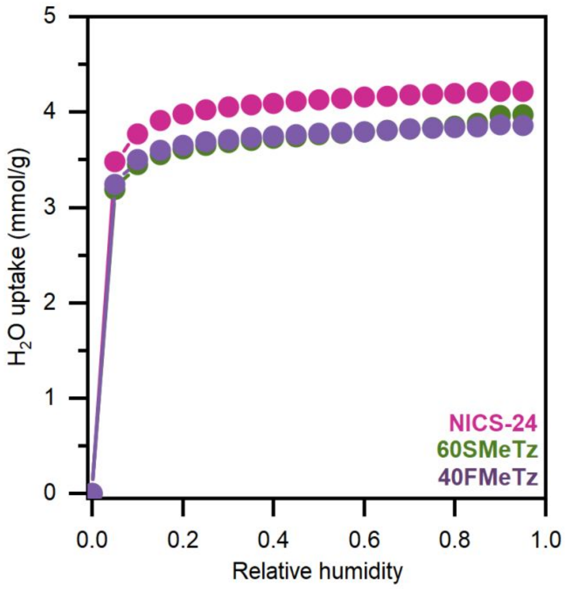

Figure S36. Water adsorption isotherms of pristine NICS-24 and both modified samples at 25 ˚C.

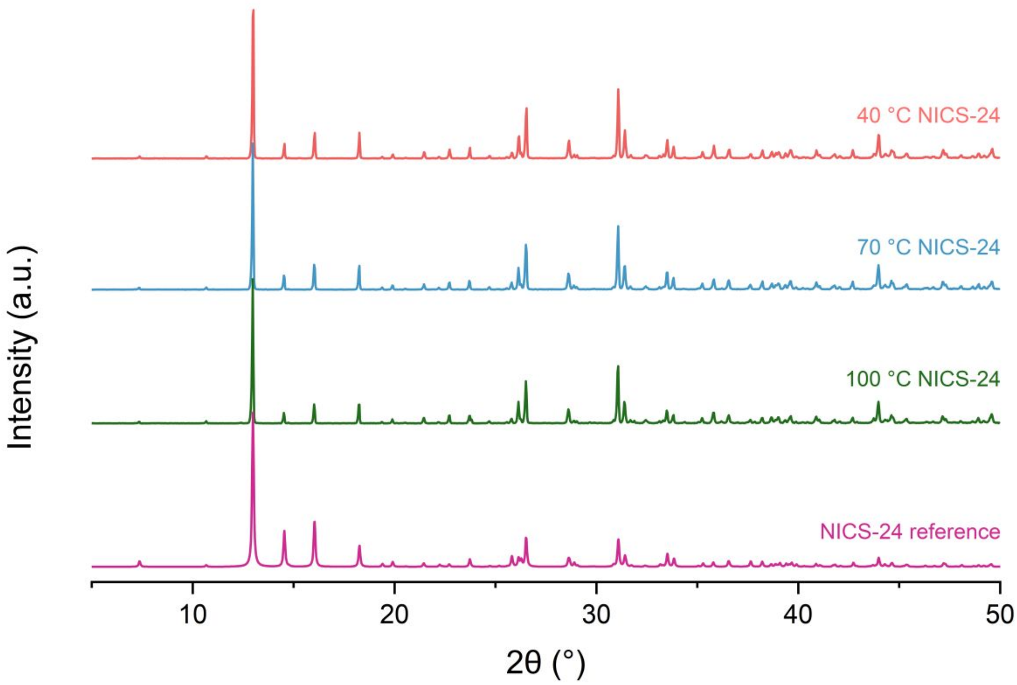

Figure S37. PXRD patterns of pristine NICS-24 after water exposure and thermal treatment at 40, 70, and 100 ˚C.

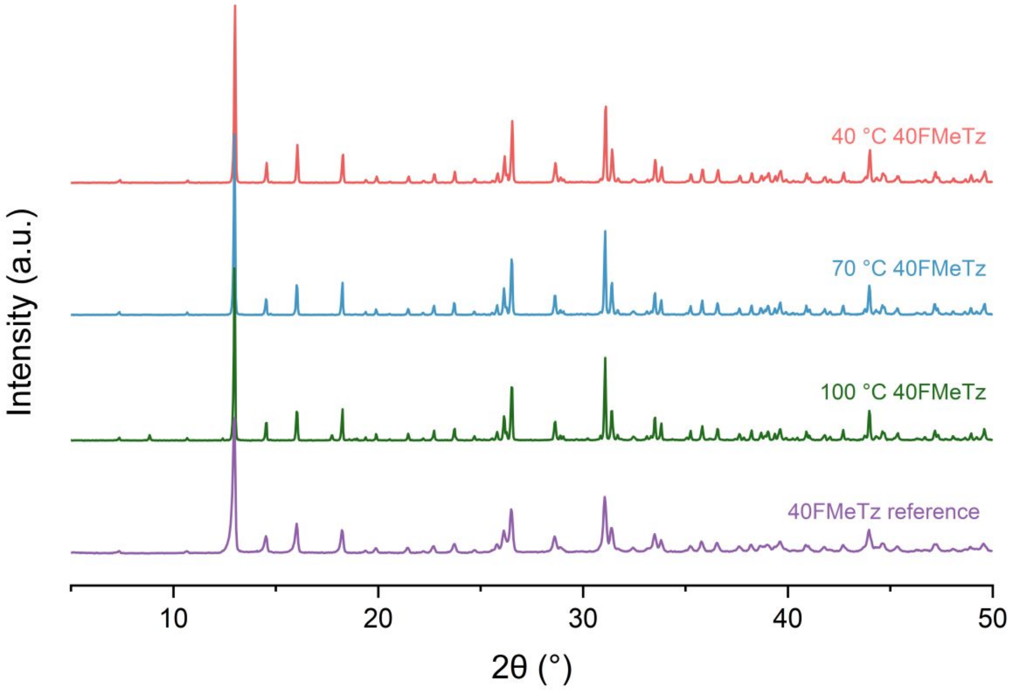

Figure S38. PXRD patterns of 40FMeTz-modofied NICS-24 after water exposure and thermal treatment at 40, 70, and 100 ˚C.

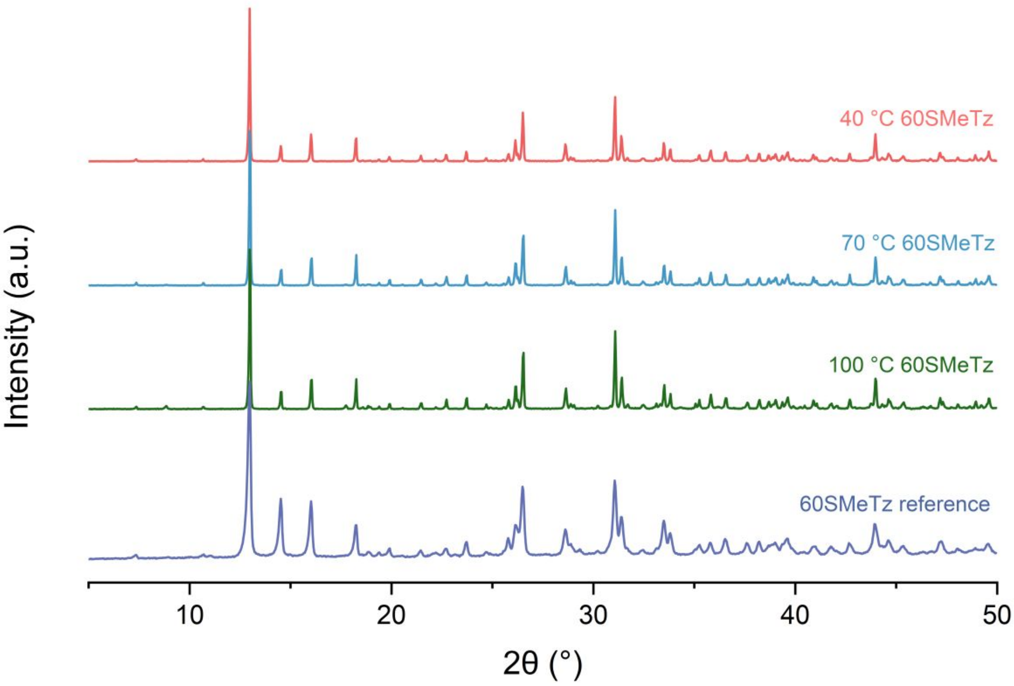

Figure S39. PXRD patterns of 60SMeTz-modified NICS-24 after water exposure and thermal treatment at 40, 70, and 100 ˚C.

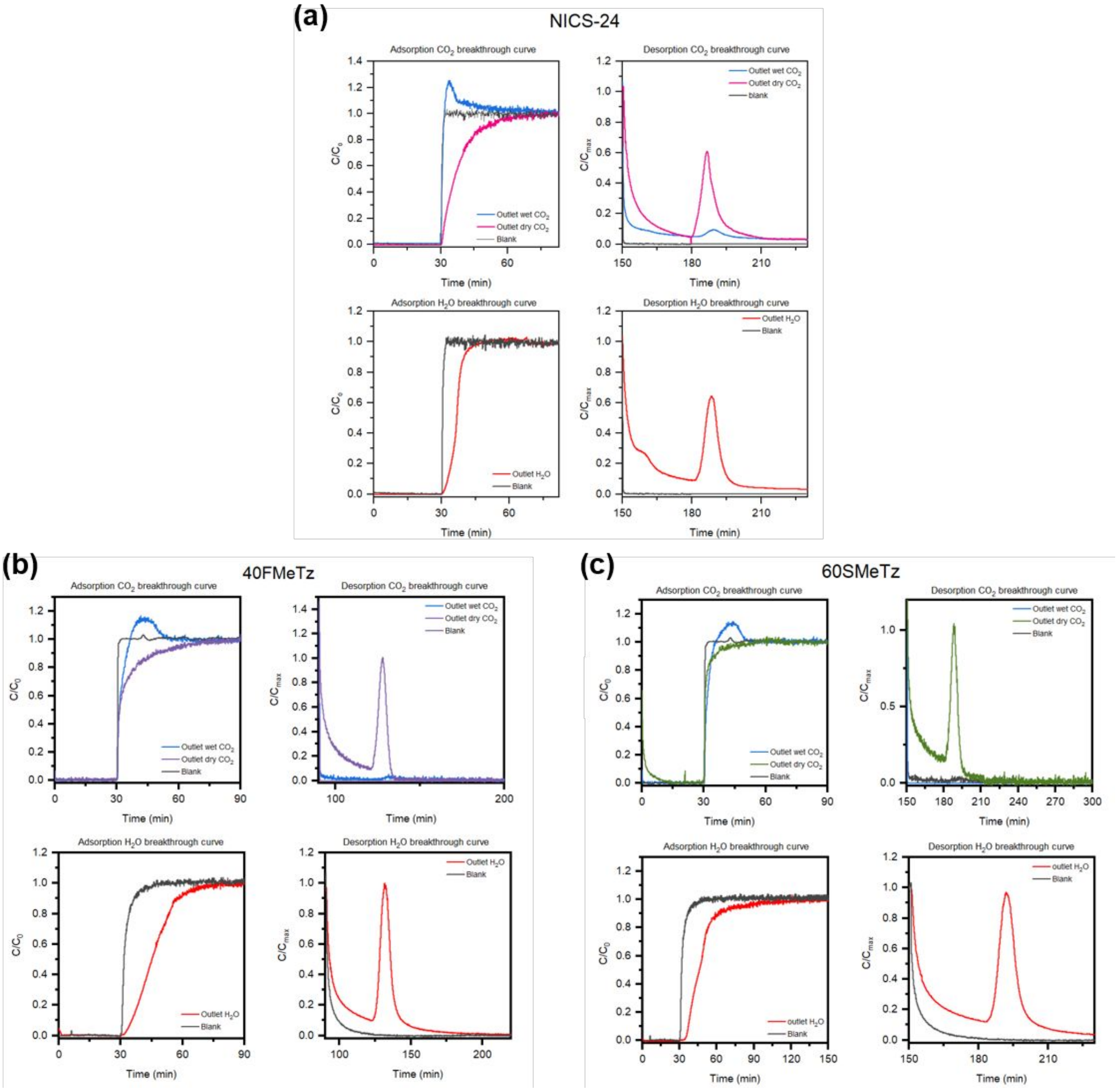

Figure S40. The dynamic breakthrough curve measurement for (a) NICS-24, (b) 40FMeTz and (c) 60SMeTz at 25˚C under humid conditions (50% RH, 1000 ppm CO_2). The 'Inlet' and 'Outlet' refer to the inlet and outlet CO_2 concentration of the fixed-bed column.

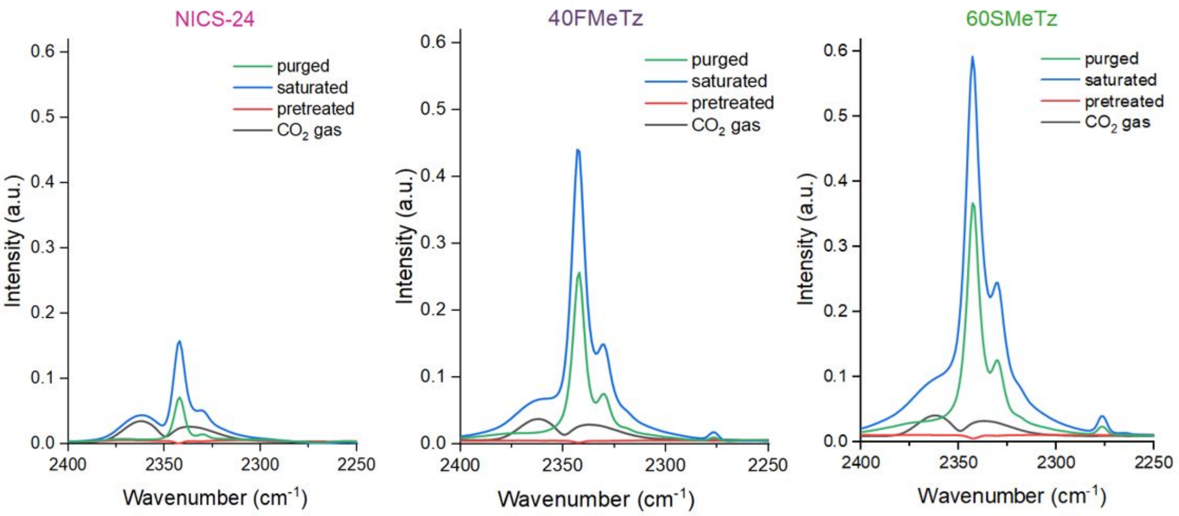

Figure S41. DRIFTS spectra at CO_2-relevant frequency region.

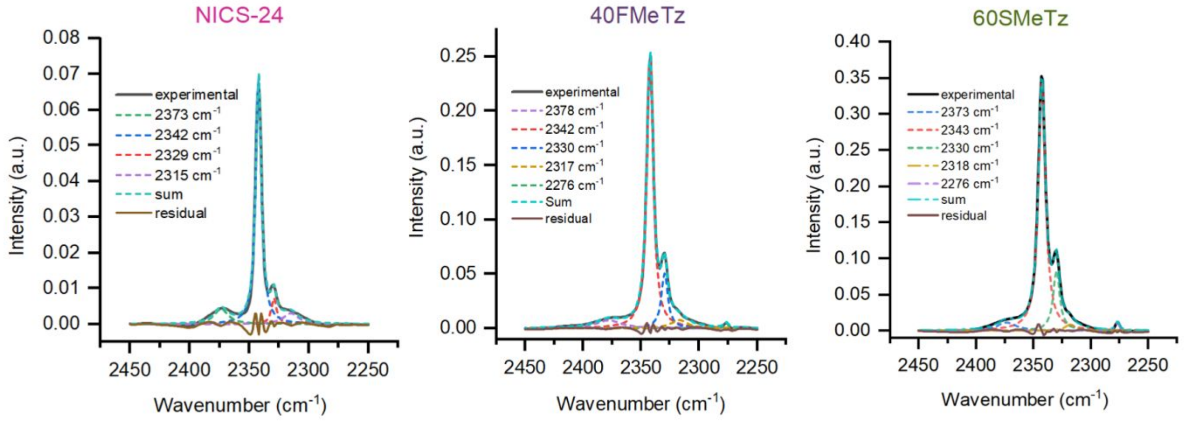

NICS-24		40FMeTz		60SMeTz	
Wavenumber(cm^{-1})	Area	Wavenumber(cm^{-1})	Area	Wavenumber(cm^{-1})	Area
2373	0.09	2378	0.31	2373	0.36
2342	0.62	2342	2.61	2343	4.06
2329	0.056	2330	0.53	2330	0.9
2315	0.08	2317	0.21	2318	0.14
		2276	0.04	2276	0.07

Figure S42. Deconvolution fits of IR bands attributed to specific CO_2 species with the integrated values provided in the table.

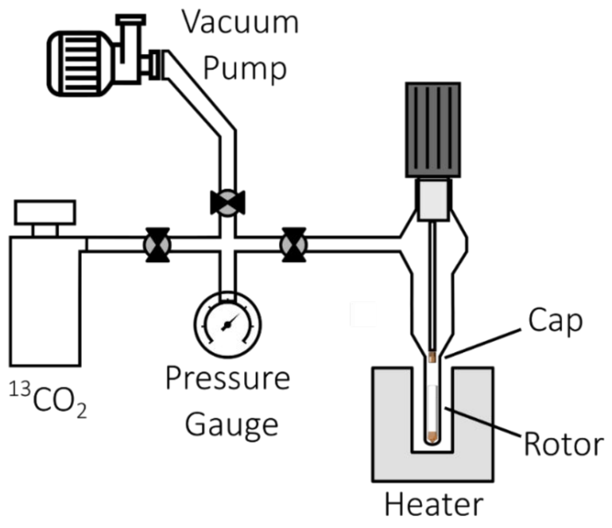

Figure S43. Schematic representation of the laboratory-built gas-dosing manifold used for the preparation of CO_2-loaded samples.

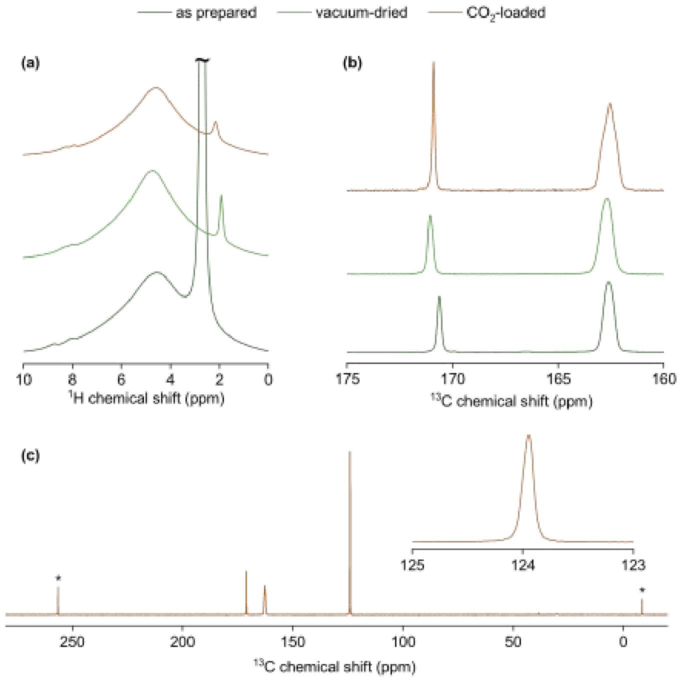

Figure S44. (a) ^{1}H MAS NMR spectra and (b) aromatic region of ^{1}H–^{13}C CP-MAS NMR spectra of as prepared, vacuum-dried and CO_2-loaded 60SMeTz. (c) ^{1}H–^{13}C CP-MAS NMR spectrum of CO_2-loaded 60SMeTz. Spinning sidebands are denoted by asterisks (*).

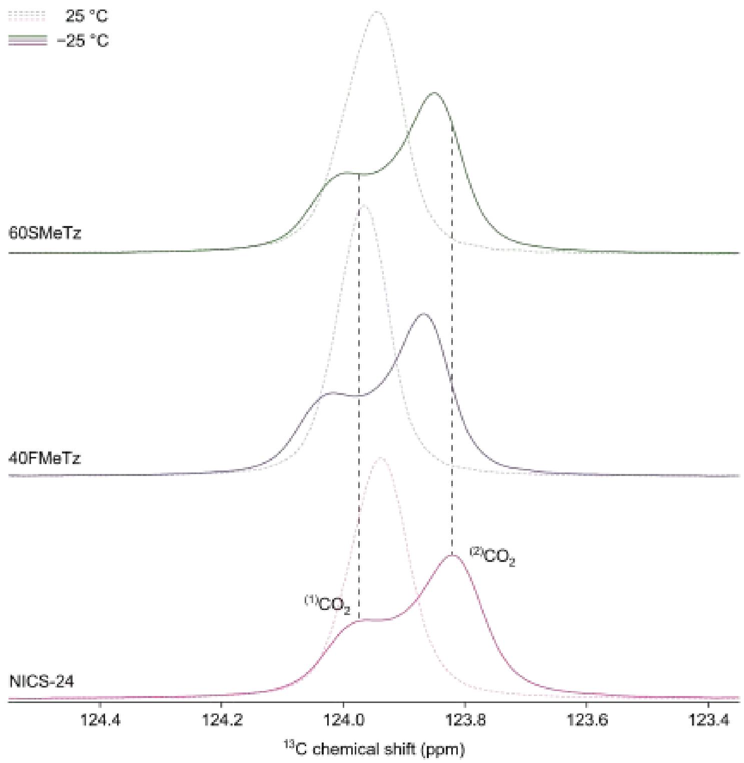

Figure S45. ^{1}H–^{13}C CP-MAS spectra of CO_2-loaded samples (physisorbed CO_2 region) recorded at room temperature and −25 °C.

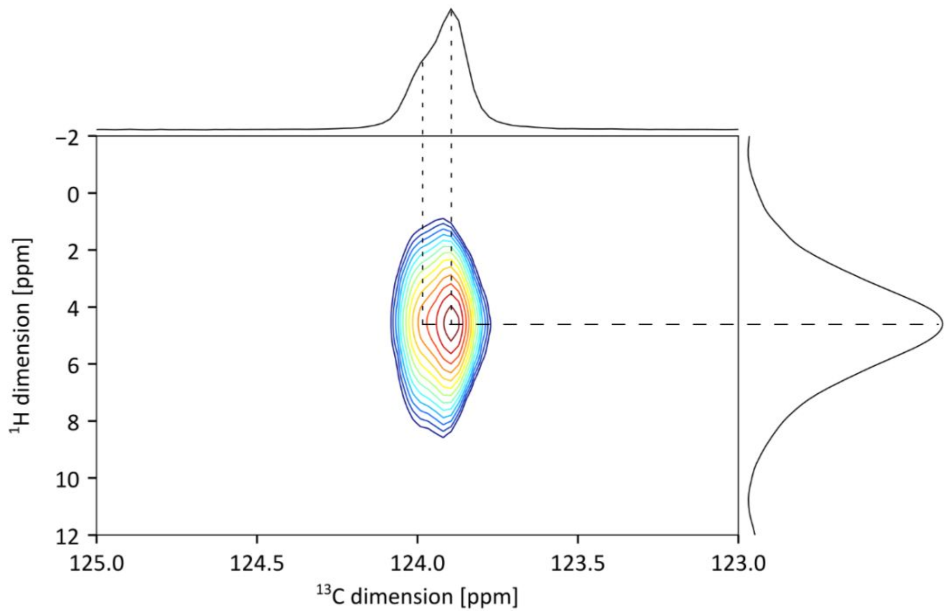

Figure S46. ^{1}H–^{13}C CP-HETCOR NMR spectrum of CO_2-loaded 60SMeTz, recorded at −25 °C.

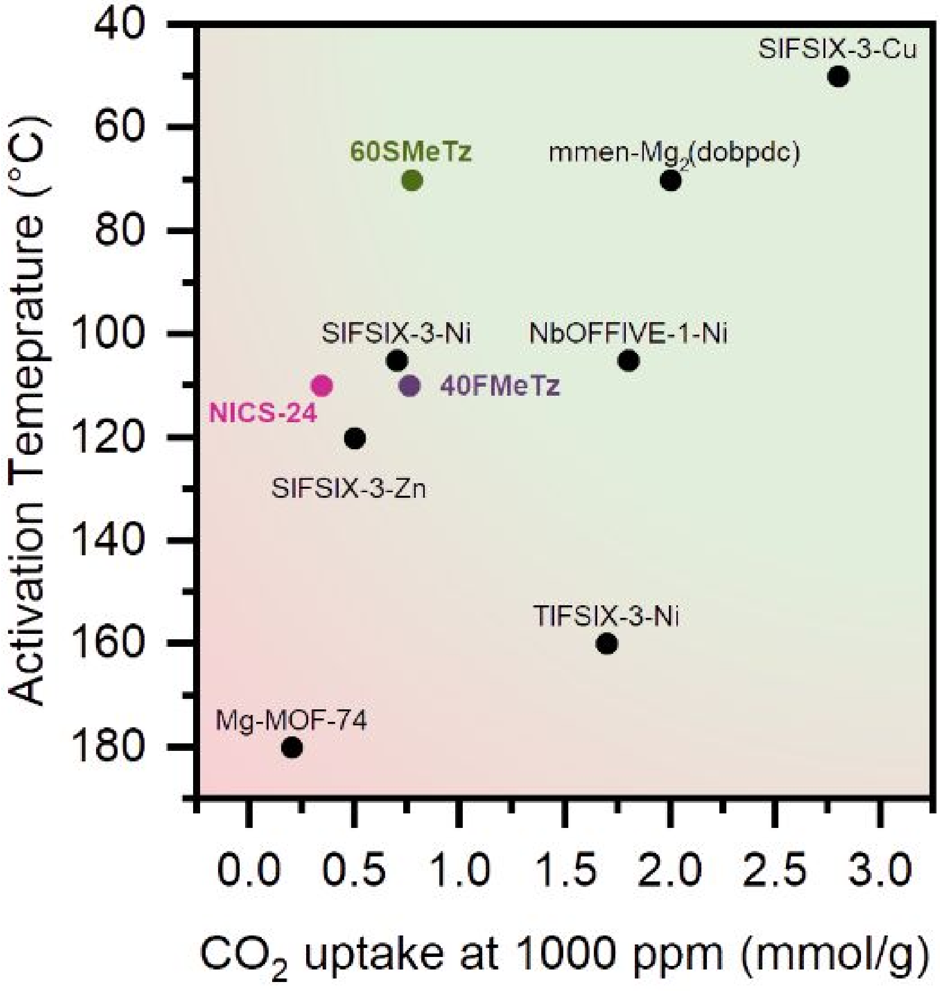

Figure S47. Comparison of trade-offs between modified NICS-24 samples (60SMeTz and 40FMeTz) with the state-of-the art MOFs used for low-concentration CO_2 capture, highlighting activation temperature versus CO_2 capacities at 1000 ppm.

Table S7. Comparison of reported CO_2 uptakes at low concentrations and the corresponding physical properties (heat of adsorption (Q_{st}) and activation temperature (T_{act})) of various MOFs tested for low-concentration CO_2 capture.

Material	CO_2 uptake at 25˚C (mmol/g)		Q_{st} (-kJ/mol)	T_{act}	Ref.
	400 ppm	*1000 ppm*			
**60SMeTz (This work)**	0.36	0.77	64^{b}	≥ 70 ˚C	/
**40FMeTz (This work)**	0.35	0.76	-	≥ 110 ˚C	/
**NICS-24**	0.13	0.34	47^{b}	≥ 110 ˚C	[1]
**NbOFFIVE-1-Ni**	1.30	1.8	50^{c}	105 ˚C	[18]
**SIFSIX-3-Ni**	0.29	0.7	50^{c}	105 ˚C	[19]
**SIFSIX-3-Cu**	1.24	2.8^{a}	54^{d}	50 ˚C	[20]
**SIFSIX-3-Zn**	0.13	0.5^{a}	45^{e}	120 ˚C	[20,21]
**TIFSIX-3-Ni**	1.15^{a}	1.7	53^{e}	160 ˚C	[18,22]
**Mg-MOF-74**	0.14^{a}	0.2	42^{b}	180 ˚C	[18]
**mmen-Mg_2(dobpdc)**	1.5^{a}	2.0^{a}	70^{c}	70 ˚C	[23,24]

[a]Data were extracted from the original publication and may be approximate. [b]At 0.1 mmol/g surface coverage. [c]At 1 mmol/g surface coverage. [d]At 0.25 mmol/g surface coverage. [e]*Near*-zero surface coverage.

REFERENCES

(1) Klemenčič, K.; Krajnc, A.; Puškarić, A.; Huš, M.; Marinič, D.; Likozar, B.; Logar, N. Z.; Mazaj, M. Amine-Functionalized Triazolate-Based Metal–Organic Frameworks for Enhanced Diluted CO_2 Capture Performance. *Angew. Chem. Int. Ed.* **2025**, *64* (14), e202424747.

(2) Lin, J. Bin; Nguyen, T. T. T.; Vaidhyanathan, R.; Burner, J.; Taylor, J. M.; Durekova, H.; Akhtar, F.; Mah, R. K.; Ghaffari-Nik, O.; Marx, S.; Fylstra, N.; Iremonger, S. S.; Dawson, K. W.; Sarkar, P.; Hovington, P.; Rajendran, A.; Woo, T. K.; Shimizu, G. K. H. A Scalable Metal-Organic Framework as a Durable Physisorbent for Carbon Dioxide Capture. *Science* **2021**, *374* (6574), 1464–1469.

(3) Kansy, J. Microcomputer Program for Analysis of Positron Annihilation Lifetime Spectra. *Nucl. Instrum. Methods Phys. Res. A* **1996**, *374* (2), 235–244.

(4) Tao, S. J. Positronium Annihilation in Molecular Substances. *J. Chem. Phys.* **1972**, *56* (11), 5499–5510.

(5) Eldrup, M.; Lightbody, D.; Sherwood, J. N. The Temperature Dependence of Positron Lifetimes in Solid Pivalic Acid. *Chem. Phys.* **1981**, *63* (1–2), 51–58.

(6) Bahmanzadegan, F.; Ghaemi, A. Mechanistic Mass Transfer Analysis of CO_2 Diffusion in Zeolite 13X@ZIF-8 Core–Shell Adsorbent. *Chem. Eng. Sci.* **2026**, *322*, 123086.

(7) Kim, D. H. Linear Driving Force Formulas for Unsteady-State Diffusion and Reaction in Slab, Cylinder and Sphere Catalyst. *AIChE J.* **2009**, *55* (3), 834–839.

(8) Hoffman, R. Solid-State Chemical-Shift Referencing with Adamantane. *J. Magn. Reson.* **2022**, *340*, 107231.

(9) van Meerten, S. G. J.; Franssen, W. M. J.; Kentgens, A. P. M. SsNake: A Cross-Platform Open-Source NMR Data Processing and Fitting Application. *J. Magn. Reson.* **2019**, *301*, 56–66.

(10) Haw, J. F.; Nicholas, J. B.; Xu, T.; Beck, L. W.; Ferguson, D. B. Physical Organic Chemistry of Solid Acids: Lessons from in Situ NMR and Theoretical Chemistry. *Acc. Chem. Res.* **1996**, *29* (6), 259–267.

(11) Zhang, W.; Ma, D.; Liu, X.; Liu, X.; Bao, X. Perfluorotributylamine as a Probe Molecule for Distinguishing Internal and External Acidic Sites in Zeolites by High-Resolution ^{1}H MAS NMR Spectroscopy. *Chem. Commun.* **1999**, No. 12, 1091–1092.

(12) Mafra, L.; Čendak, T.; Schneider, S.; Wiper, P. V.; Pires, J.; Gomes, J. R. B.; Pinto, M. L. Structure of Chemisorbed CO_2 Species in Amine-Functionalized Mesoporous Silicas Studied by Solid-State NMR and Computer Modeling. *J. Am. Chem. Soc.* **2017**, *139* (1), 389–408.

(13) Forse, A. C.; Milner, P. J.; Lee, J. H.; Redfearn, H. N.; Oktawiec, J.; Siegelman, R. L.; Martell, J. D.; Dinakar, B.; Porter-Zasada, L. B.; Gonzalez, M. I.; Neaton, J. B.; Long, J. R.; Reimer, J. A. Elucidating CO_2 Chemisorption in Diamine-Appended Metal-Organic Frameworks. *J. Am. Chem. Soc.* **2018**, *140* (51), 18016–18031.

(14) Pascual-Izarra, C.; Dong, A. W.; Pas, S. J.; Hill, A. J.; Boyd, B. J.; Drummond, C. J. Advanced Fitting Algorithms for Analysing Positron Annihilation Lifetime Spectra. *Nucl. Instrum. Methods Phys. Res. A* **2009**, *603* (3), 456–466.

(15) Dolzhenko, A. V.; Dolzhenko, A. V.; Chui, W. K. Synthesis of 5,7-Diamino[1,2,4] Triazolo[1,2-a][1,3,5]Triazines via Annulation of 1,3,5-Triazine Ring onto 3(5)-Amino-1,2,4-Triazoles. *Heterocycles* **2007**, *71* (2), 429–436.

(16) Ba, Y. Q.; Wang, Y. S.; Li, T. Y.; Zheng, Z.; Hao, G. P.; Lu, A. H. Fine Tuning CO_2 Adsorption and Diffusion Behaviors in Ultra-Microporous Carbons for Favorable CO_2 Capture at Moderate Temperature. *Sust. Chem. Clim. Act.* **2023**, *2*, 100015.

(17) Niu, J.; Li, H.; Tao, L.; Fan, Q.; Liu, W.; Tan, M. C. Defect Engineering of Low-Coordinated Metal-Organic Frameworks (MOFs) for Improved CO_2 Access and Capture. *ACS Appl. Mater. Interfaces* **2023**, *15* (26), 31664–31674.

(18) Kumar, A.; Hua, C.; Madden, D. G.; O'Nolan, D.; Chen, K. J.; Keane, L. A. J.; Perry, J. J.; Zaworotko, M. J. Hybrid Ultramicroporous Materials (HUMs) with Enhanced Stability and Trace Carbon Capture Performance. *Chem. Commun.* **2017**, *53* (44), 5946–5949.

(19) Bhatt, P. M.; Belmabkhout, Y.; Cadiau, A.; Adil, K.; Shekhah, O.; Shkurenko, A.; Barbour, L. J.; Eddaoudi, M. A Fine-Tuned Fluorinated MOF Addresses the Needs for Trace CO_2 Removal and Air Capture Using Physisorption. *J. Am. Chem. Soc.* **2016**, *138* (29), 9301–9307.

(20) Shekhah, O.; Belmabkhout, Y.; Chen, Z.; Guillerm, V.; Cairns, A.; Adil, K.; Eddaoudi, M. Made-to-Order Metal-Organic Frameworks for Trace Carbon Dioxide Removal and Air Capture. *Nat. Commun.* **2014**, *5* (1), 1–7.

(21) Uemura, K.; Maeda, A.; Maji, T. K.; Kanoo, P.; Kita, H. Syntheses, Crystal Structures and Adsorption Properties of Ultramicroporous Coordination Polymers Constructed from Hexafluorosilicate Ions and Pyrazine. *Eur. J. Inorg. Chem.* **2009**, *2009* (16), 2329–2337.

(22) Mukherjee, S.; Sikdar, N.; O'Nolan, D.; Franz, D. M.; Gascón, V.; Kumar, A.; Kumar, N.; Scott, H. S.; Madden, D. G.; Kruger, P. E.; Space, B.; Zaworotko, M. J. Trace CO_2 Capture by an Ultramicroporous Physisorbent with Low Water Affinity. *Sci. Adv.* **2019**, *5* (11), 9171–9200.

(23) Park, J.; Park, J. R.; Choe, J. H.; Kim, S.; Kang, M.; Kang, D. W.; Kim, J. Y.; Jeong, Y. W.; Hong, C. S. Metal-Organic Framework Adsorbent for Practical Capture of Trace Carbon Dioxide. *ACS Appl. Mater. Interfaces* **2020**, *12* (45), 50534–50540.

(24) Sinha, A.; Darunte, L. A.; Jones, C. W.; Realff, M. J.; Kawajiri, Y. Systems Design and Economic Analysis of Direct Air Capture of CO_2 through Temperature Vacuum Swing Adsorption Using MIL-101(Cr)-PEI-800 and

mmen-Mg_2(Dobpdc) MOF Adsorbents. *Ind. Eng. Chem. Res.* **2017**, *56* (3), 750–764.
